# MAP4K4 expression in cardiomyocytes: multiple isoforms, multiple phosphorylations and interactions with striatins

**DOI:** 10.1042/BCJ20210003

**Published:** 2021-06-11

**Authors:** Stephen J. Fuller, Nick S. Edmunds, Liam J. McGuffin, Michelle A. Hardyman, Joshua J. Cull, Hajed O. Alharbi, Daniel N. Meijles, Peter H. Sugden, Angela Clerk

**Affiliations:** 1 School of Biological Sciences, University of Reading, Whiteknights Campus, Reading RG6 2AS, U.K.; 2 Molecular and Clinical Sciences Institute, St George's University of London, London SW17 0RE, U.K.

**Keywords:** cardiac myocytes, protein phosphatases, protein-serine-threonine kinases

## Abstract

The Ser/Thr kinase MAP4K4, like other GCKIV kinases, has N-terminal kinase and C-terminal citron homology (CNH) domains. MAP4K4 can activate c-Jun N-terminal kinases (JNKs), and studies in the heart suggest it links oxidative stress to JNKs and heart failure. In other systems, MAP4K4 is regulated in striatin-interacting phosphatase and kinase (STRIPAK) complexes, in which one of three striatins tethers PP2A adjacent to a kinase to keep it dephosphorylated and inactive. Our aim was to understand how MAP4K4 is regulated in cardiomyocytes. The rat MAP4K4 gene was not properly defined. We identified the first coding exon of the rat gene using 5′-RACE, we cloned the full-length sequence and confirmed alternative-splicing of MAP4K4 in rat cardiomyocytes. We identified an additional α-helix C-terminal to the kinase domain important for kinase activity. In further studies, FLAG-MAP4K4 was expressed in HEK293 cells or cardiomyocytes. The Ser/Thr protein phosphatase inhibitor calyculin A (CalA) induced MAP4K4 hyperphosphorylation, with phosphorylation of the activation loop and extensive phosphorylation of the linker between the kinase and CNH domains. This required kinase activity. MAP4K4 associated with myosin in untreated cardiomyocytes, and this was lost with CalA-treatment. FLAG-MAP4K4 associated with all three striatins in cardiomyocytes, indicative of regulation within STRIPAK complexes and consistent with activation by CalA. Computational analysis suggested the interaction was direct and mediated via coiled-coil domains. Surprisingly, FLAG-MAP4K4 inhibited JNK activation by H_2_O_2_ in cardiomyocytes and increased myofibrillar organisation. Our data identify MAP4K4 as a STRIPAK-regulated kinase in cardiomyocytes, and suggest it regulates the cytoskeleton rather than activates JNKs.

## Introduction

MAP4K4 was first cloned from mouse cells in 1997 as NIK (Nck-interacting kinase) [1] and later from human cells as HGK (HPK/GCK-like kinase) [2]. MAP4K4 is a STE20 Ser/Thr protein kinase, belonging to the Germinal Center Kinase (GCK) family, with a conserved N-terminal catalytic domain and C-terminal regulatory domain. MAP4K4, TNIK (MAP4K7) and MINK (MAP4K6) constitute the GCKIV subfamily, having a conserved citron homology (CNH) domain towards the C terminus and a large unstructured region between the kinase and the CNH [3]. MAP4K4 is highly expressed in the heart, with potentially the highest mRNA levels across all normal adult tissues studied [1,2]. Perhaps unsurprisingly, MAP4K4 activity is therefore linked to cardiovascular disease and heart failure, but it is also associated with other major diseases including cancer [4,5]. There is, therefore, considerable focus on identifying selective small molecule inhibitors for the kinase.

MAP4K4 was identified as an upstream activator of the c-Jun N-terminal kinase (JNK) family of the mitogen-activated protein kinases (MAPKs), interacting with MEKK1 or TAK1, and potentially mediating the signal from TNFα [1,2]. These studies were based on co-expression of each of the various components of the pathway, rather than the investigation of endogenous proteins, but activation of JNKs by MAP4K4 promotes apoptosis in motor neurons and a small molecule inhibitor of MAP4K4 kinase (MAP4K4i 29) protects against cell death [6]. MAP4K4 is reported to be activated by oxidative stresses in cardiomyocytes and associated with activation of JNKs in the heart [7]. A small molecule inhibitor that targets MAP4K4 (DMX5804) protects stem-cell derived ‘cardiomyocytes' from apoptosis [7,8]. However, the inhibitors so far developed for MAP4K4 are not entirely specific and those used inhibit the other GCKIV kinases with similar potencies, along with affecting the activities of some non-GCK kinases albeit at lower potencies [6–8].

Apart from its role in JNK signalling, MAP4K4 is implicated in regulating cytoskeletal structures, a role that can impact on cell adhesion and migration. Overall, MAP4K4 appears to destabilise focal adhesions such that loss or inhibition of the kinase results in stabilisation of adhesion complexes and reduced migration in cell lines and endothelial cells [9,10]. The effect may be mediated via MAP4K4 phosphorylation of moesin [10], or other cytoskeletal proteins (e.g. FARP1) [11]. This influence of MAP4K4 on cell adhesion and migration has clear implications for cancer and blood vessel permeability.

The regulation of MAP4K4 is not fully understood, but there is increasing evidence for regulation via a striatin-interacting phosphatase and kinase (STRIPAK) multiprotein complex [12]. Here, a striatin molecule (Strn, Strn3 or Strn4) serves as a B′′′ scaffolding subunit for protein phosphatase 2A (PP2A) that maintains striatin-associated MAP4K4 in a dephosphorylated and inactive form. Inhibition of PP2A or removal of the kinase from the complex results in MAP4K4 autophosphorylation and activation. The precise mechanisms involved *in vivo* remain unclear, but potentially depend on other constituents of the complex. In cancer cells, MAP4K4 interacts directly with Strn4 and disruption of the complex results in oncogenic transformation [13–15]. It is not clear if the interaction is only with Strn4, rather than the other striatins, or if the interaction is direct or mediated via another protein. Whilst STRIPAK complexes are linked to the regulation of cell adhesion and migration [12], it is not clear how they relate to the activation of JNKs. However, JNKs are a stress-responsive signal and so may be activated consequent to modulation of cell adhesion or migration.

The kinase domain of MAP4K4 forms a focus for inhibitor development and the crystal structure is well-defined, but this domain (residues 26–290) is a relatively small part of the full-length protein (>1200 residues) [1,2]. GCKIVs have a conserved CNH domain and a coiled-coil region towards the N-terminus of the linker between the kinase and the CNH domain [16]. The function of these is not fully established although the CNH domain can interact with the small G protein Rap2 in its GTP-bound (i.e. activated) form [17,18]. Studies in glioblastoma cell lines identified multiple isoforms of MAP4K4 resulting from alternative splicing in the disordered part of the linker region [16], but the significance of these is unknown and the splice variants are not fully defined. Here, we investigated MAP4K4 expressed in cardiomyocytes. Cardiac research relies on studies of rat cardiomyocytes and the heart, so we first defined the rat MAP4K4 gene, confirming the start codon, exon structure and alternative splicing in primary cells. We determined that MAP4K4 interacted with each of the striatins in cardiomyocytes, was maintained in an inactive form by Ser/Thr protein phosphatases, and activation (by phosphatase inhibition) resulted in hyperphosphorylation of the linker region, not just phosphorylation of the activation loop in the kinase domain. These and other data indicated that cardiomyocyte MAP4K4 is involved with STRIPAK regulation of the cytoskeleton, rather than playing a direct role in JNK signalling and apoptosis.

## Experimental

### Neonatal rat cardiomyocytes

Studies were performed in accordance with European Parliament Directive 2010/63/EU on the protection of animals used for scientific purposes, with local institutional animal care committee procedures (University of Reading) and the U.K. Animals (Scientific Procedures) Act 1986. Sprague-Dawley female rats plus 2–3 d neonates (Charles River U.K.) were housed overnight at the BioResource Unit at University of Reading (UK registered with a Home Office certificate of designation) in open top NKP cages (total area 1632 cm^2^). Cages were supplied with aspen sawdust bedding, sizzle nesting, cardboard tunnels and housing. Additional enrichment included chew sticks and millet to encourage foraging behaviour. Animals were provided with water and food (SDS RM3 expanded pelleted food for rats) *ad libitum*, with a 12 : 12 light/dark cycle and room temperature of 21°C. Neonatal rats were killed by cervical dislocation followed by decapitation, and ventricular myocytes prepared essentially as previously described [19]. Cardiomyocytes were resuspended in plating medium [Dulbecco's modified Eagle's medium (DMEM)/medium 199 [4 : 1 (v/v)] containing 15% (v/v) foetal calf serum (FCS; Life Technologies) and 100 U/ml penicillin and streptomycin (Gibco)]. For biochemical studies, viable cardiomyocytes were plated at a density of 4 × 10^6^ cells/dish on 60 mm Primaria dishes (BD BioSciences) or 2 × 10^6^ cells/dish on 35 mm Primaria dishes (for kinase assays), pre-coated with sterile 1% (w/v) gelatin (Sigma–Aldrich). For immunostaining experiments, cardiomyocytes were plated at a density of 1 × 10^6^ cells/dish on 35 mm Primaria dishes containing glass coverslips pre-coated with 1% (w/v) gelatin (Sigma–Aldrich) followed by laminin (20 µg/ml, Sigma–Aldrich).

For experiments with uninfected cardiomyocytes, the plating medium was withdrawn after 18 h and cells were incubated in serum-free maintenance medium (DMEM/medium 199 [4 : 1 (v/v)] containing 100 U/ml penicillin and streptomycin) for a further 24 h prior to experimentation. For biochemical studies, cells were confluent and beating spontaneously. For immunostaining experiments, cells were beating spontaneously and ∼50% confluent. In experiments with overexpressed MAP4K4, cells were infected with adenovirus at the time of plating and cells were incubated for 42 h before the medium was changed to serum-free maintenance medium. Unless otherwise stated, cardiomyocytes were treated with 200 nM final concentration of CalA (Enzo Life Sciences) dissolved in DMSO (1/2000 dilution). Controls were conducted with 1/2000 dilution of DMSO alone.

### 5′ RACE

5′ RACE was performed using a Clontech SMARTer^TM^ RACE cDNA amplification kit largely according to the manufacturer's instructions. Primers were from Eurofins Genomics. RACE-ready cDNA was generated from rat heart mRNA using the SMARTer II A oligonucleotide (5′-AAGCAGTGGTATCAACGCAGAGTACXXXXX-3′; X = undisclosed proprietary sequence), 5′-CDS primer A [5′-(T)_25_V N-3′; V = A, G or C; N = A, C, G or T] using Smartscribe Reverse Transcriptase. In brief, mRNA was incubated with the primers (72°C, 3 min, then 42°C, 2 min) before the addition of RNase inhibitor and Smartscribe Reverse Transcriptase. Samples were incubated at 42°C (90 min) then 70°C (10 min), and products diluted with 200 µl Tricine-EDTA buffer. Products were amplified using Taq polymerase (Invitrogen) with Short RACE forward primer (5′-CTAATACGACTCACTATAGGGC-3′) and a MAP4K4-specific reverse primer (5′-CCGCAGGGACGACAGGTCGATGTCCACC-3′) using five cycles of 94°C (30 s) and 72°C (3 min), followed by five cycles of 94°C (30 s), 70°C (30 s) and 72°C (3 min), then 40 cycles of 94°C (30 s), 68°C (30 s) and 72°C (3 min). The product was purified and ligated into a pDrive cloning vector (Qiagen) used to transform DH5α cells. Clones were screened for MAP4K4 using a forward primer from the predicted exon containing the start codon (5′-ATGGCGAACGACTCTCC-3′) and a vector-specific reverse primer (M13Rev: 5′-CAGGAAACAGCTATGAC-3′). A clone with a product of the correct size (∼150 bp) was selected for amplification and sequencing using M13Rev primers. DNA sequencing was performed by Source Bioscience. The .ab1 file was exported to 4Peaks software (Nucleobytes) for presentation.

### Rat MAP4K4 cloning and vector construction

Primers used for MAP4K4 cloning and mutations (Supplemental Table S1) were from Eurofins Genomics. Restriction enzymes were from New England Biolabs. The presence of Kpn1 and HindIII restriction sites in the rat MAP4K4 coding sequence necessitated a complex cloning strategy as these restriction enzymes are used for insertion into the multiple cloning site (MCS) of the cloning vector, pShuttle-CMV (Stratagene). The 3′ segment was generated by PCR from two smaller PCR products amplified using *Pfu* polymerase (Promega) with primer pairs F1/R1 and F2/R2 and rat neonatal cardiac myocyte cDNA as template. Secondary PCR reactions were then carried out with primers R1/F2 and a mix of the two products. This produced PCR products with 5′-flanking Xho1 and 3′-flanking HindIII sites subsequently used for subcloning into the corresponding sites in the MCS of FLAG-shuttle (a modified pShuttle-CMV vector with a FLAG sequence inserted immediately 5′ to the Kpn1 site in the MCS [20]). The 5′ segment of MAP4K4 coding sequence was generated from two smaller fragments amplified using *Pfu* polymerase with primer pair F3/R3 and F4/R4 and rat neonatal cardiac myocyte cDNA as template. Secondary PCR reactions were then carried out using primers R3/F4 and a mix of the two products. The product of this reaction contained a Kpn1 site engineered immediately 5′ to the ATG start site and an endogenous Kpn1 site towards the 3′ end as part of the MAP4K4 coding sequence. These Kpn1 sites were used to insert the product upstream of the 3′ segment in the FLAG-shuttle vector. Samples of ligation reactions were used to transform DH5α cells and individual clones screened for the insertion and correct orientation of the 5′ fragment. Positive clones were expanded and sequenced fully (Source Bioscience). The MAP4K4 sequence has 100% identity with the predicted variant X22 (XM_008767015.2) and is homologous with the murine sequence first identified and termed NIK [1].

### Assessment of alternative splicing and cloning of endogenous rat MAP4K4 (variant X16: XM_017596342.1)

Clones were identified with and without exon 21 (encoding the GEV sequence). This was verified by PCR using a forward primer in exon 14 (5′-AGATCCTGCAGCAGCAGCTG-3′) and reverse primers across the exon boundary with or without exon 21 (GEV+: 5′-AACGCAGTCAAGTCCACTTCTCC-3′; GEV−: 5′-AACGCAGTCAAGTCAGCAGGTTT-3′), using Taq polymerase. A positive control used a reverse primer in exon 22 (5′-CTCGAAGCTCTTTGGCCAAC-3′). PCR conditions used 94°C (3 min), followed by 35 cycles of 94°C (30 s), 58°C (30 s) 72°C (30 s) then 72°C (2 min). To assess the potential for alternative splicing of MAP4K4 in cardiomyocytes, PCR products were generated from the RACE products of cDNA derived from neonatal rat cardiomyocytes, using Taq polymerase with a forward primer in exon 14 (5′-AGATCCTGCAGCAGCAGCTG-3′) and reverse primer in exon 19 (5′-CTCACTCTGAAGCGTTCACC-3′), and with a forward primer in exon 22 (5′ AGTCTGGGACCACGGATGAG-3′) and reverse primer across the boundary of exons 26–27 (5′-TCACTCCCCATAAGGCAGC-3′). To identify a *bona fide* MAP4K4 transcript expressed in rat cardiomyocytes from the alternatively spliced region, a PCR product was generated using Taq polymerase and rat cardiomyocyte cDNA using primers F9 (exon 13) and R8 (exon 31). The products were T/A cloned into pDrive (Qiagen) and the ligation mixture used to transform XL10Gold cells. A positive clone (c25) was identified and sequenced. This corresponded to predicted variant X16 (XM_017596342.1).

### Preparation of MAP4K4 mutants

Plasmid constructs harbouring mutations of specific amino acids were prepared by site-directed mutagenesis. For MAP4K4(K54R) (mutated in the ATP binding site), overlapping PCR products were generated using primer pairs F5 with R5, and F6 with R6. These products were used for subsequent PCR with primers F5 with R6. The product of this reaction had Sal1 restriction enzyme sites towards each end which were cut and used to insert the cassette into the equivalent sites in the wild-type (WT) MAP4K4 construct. The T187A activation loop phosphorylation site mutant was made by using primer R7 instead of R5, and primer F7 instead of F6. The T187D mutant was made by replacing primer R5 with R12, and primer F6 with F13. For the T319A/S324A/S326A triple mutant, overlapping PCR products were generated using primer pairs F10 with R14, and F15 with R3. These products were used for a subsequent PCR with primers F10 with R3. Products were cut with Xho1 and AflII enzymes for insertion into the equivalent sites in the WT MAP4K4 construct. For the MAP4K4(T309A) mutant, overlapping PCR products were generated using primer pairs F10 with R15, and F16 with R3. These products were used for a subsequent PCR with primers F10 with R3. Products were cut with Xho1 and AflII enzymes for insertion into the equivalent sites in WT MAP4K4 construct. For the T309A/T319A/S324A/S326A quadruple mutant, the MAP4K4(T309A) construct was used as a template for PCR products produced with F10 and R14, and using F15 with R3. These products were used for a subsequent PCR with primers F10 with R3. Products were cut with Xho1 and AflII enzymes for insertion into the equivalent sites in WT MAP4K4 construct.

For the MAP4K4Δ(304–326) deletion construct, a PCR product was made using primers F8/R3 and WT-MAP4K4 as a template, and then using the unique AflII and XhoI sites to replace the homologous region in the WT-MAP4K4 construct. The MAP4K4Δ(924–1233) deletion construct was made using F4 and R13 primers to amplify the region from the start codon to residue 924 using WT-MAP4K4 as a template, and then using Xho1/EcoRV sites to replace the sequence from the Xho1 site to the stop codon in the WT-MAP4K4 construct. For the MAP4K4Δ(303–552) deletion construct, the WT-MAP4K4 construct was cut using AflII and Xho1 nucleases and the products treated with mung bean nuclease (1 µl, 30 min) to maintain the reading frame before re-ligation. For the MAP4K4Δ(552–924) deletion construct, the CNH domain was amplified, inserting an Xho1 site at the 5′ end using primers F14 and R11 (with an EcoRV at the 3′ end). Following digestion with Xho1/EcoRV, the product was cloned into the same sites of the WT-MAP4K4 construct.

### MAP4K4 adenovirus production

Adenoviruses harbouring MAP4K4 expression cassettes were generated using the AdEasy™ XL Adenoviral Vector System (Stratagene). Briefly, MAP4K4 constructs prepared in the pShuttle vector were digested with Pme1 to separate the left and right homology arms and the linearised vector used to transfect BJ5183-AD-1 cells. These contain the adenoviral vector and permit recombination with the shuttle vector. Recombination positive clones were identified by screening, and miniprep DNA prepared and used to transform XL10Gold cells. These were expanded for bulk isolation of the adenoviral plasmid which was subsequently cut with Pac1 to release the kanamycin selection cassette and generate the final, linear recombinant adenoviral vector which was purified. Human Embryonic Kidney 293 (HEK293) cell cultures were cultured in DMEM containing 10% (v/v) FCS, 2 mM l-glutamine and 50 U/ml penicillin and streptomycin. DNA was transfected into HEK293 cells using FuGENE® HD (Promega) to produce the final recombinant adenovirus. This was then subjected to several rounds of amplification to increase the titre.

### HEK293 cell cultures

To generate HEK293 cells with stable expression of MAP4K4 (WT-MAP4K4-HEK293), sub-confluent HEK293 cells in 60 mm dishes were transformed using FuGENE® HD and subjected to selection with 400 μg/ml G418 through multiple rounds of passaging until a constant level of expression of the transgene (monitored with an anti-FLAG® western blot) was observed. Cells were subsequently expanded, aliquoted and frozen for later use. Cells from frozen stocks were plated on 100 mm dishes in 4 ml DMEM containing 10% (v/v) FCS, 2 mM l-glutamine, 100 U/ml penicillin and streptomycin and 400 μg/ml G418. Cells were subsequently passaged into 60 mm dishes and cultured for 16 h prior to experimentation.

For transfection experiments, HEK293 cells were grown to 80% confluence in 60 mm dishes using DMEM containing 10% (v/v) FCS, 2 mM l-glutamine, 100 U/ml penicillin and streptomycin. FuGENE® HD transfection reagent (10 µl, Promega) was mixed with 10 µl plasmid (0.5 mg/ml) plus 200 µl DMEM, allowed to stand for 10 min, then added drop-wise to the dishes. Cells were cultured for 16 h prior to experimentation.

### Sample preparation for immunoblotting

Incubation of HEK293 cells with CalA for longer than 10 min caused cells to detach from the tissue culture dish, so for these experiments, cells were transferred to tubes in PBS, collected by centrifugation (1 min, 500 g), washed in ice-cold PBS and resuspended in buffers indicated below. Cardiomyocytes remained adherent for the duration of the experiments conducted.

Total cell extracts were prepared largely as previously described [19]. Briefly, cardiomyocytes in a 60 mm dish were collected into glycerophosphate buffer [20 mM β-glycerophosphate pH 7.5, 50 mM NaF, 2 mM EDTA, 1% (v/v) Triton X-100, 0.2  mM Na_3_VO_4_ 5 mM dithiothreitol] containing protease and phosphatase inhibitors (200 µM leupeptin, 10 µM trans-epoxy succinyl-l-leucylamido-(4-guanidino)butane, 300 µM phenylmethylsulphonylfluoride, 2 µM microcystin). Extracts were incubated on ice (10 min), then centrifuged (10 000×***g***, 5 min, 4°C). A sample was taken for protein assay and the remaining supernatants boiled with 0.33 vol sample buffer [0.33 M Tris–HCl pH 6.8, 10% (w/v) SDS, 13% (v/v) glycerol, 133 mM dithiothreitol, 0.2 mg/ml bromophenol blue].

Cytosolic and nuclear-enriched fractions from cardiomyocytes were prepared according to Dignam et al. [21] and as previously described [22]. In brief, 4 × 10^6^ cardiomyocytes were collected into 150 µl hypotonic buffer containing protease and phosphatase inhibitors and incubated on ice (10 min). Extracts were centrifuged (10 000×***g***, 5 min, 4°C) and supernatants (soluble cytosolic proteins) were boiled with 0.33 vol sample buffer. The pellets (nuclear-enriched) were extracted on ice with 50 µl high salt buffer (60 min), samples were centrifuged (10 000×***g***, 5 min, 4°C) and the extracts boiled with 25 µl sample buffer.

For immunoprecipitation of FLAG-tagged MAP4K4 for immunoblotting, cells in a 60 mm dish were collected into 150 µl immunoprecipitation buffer [20 mM Tris pH 7.5, 1 mM EDTA, 10% (v/v) glycerol, 1% (v/v) Triton X-100, 100 mM KCl, 5 mM NaF, 0.2 mM Na_3_VO_4_, 5 mM MgCl_2_, 0.05% (v/v) 2-mercaptoethanol] containing protease and phosphatase inhibitors. Extracts were homogenised briefly, then kept on ice (15 min) before centrifugation (12 000×***g***, 5 min). A sample of the supernatant was boiled with an equal volume of sample buffer for assessment of proteins in the total extract. The remaining supernatants were incubated with 30 µl of a 1 : 1 slurry of EZ-view FLAG resin (Sigma–Aldrich) equilibrated with immunoprecipitation buffer (4°C, overnight with rotation). The samples were centrifuged (12 000×***g***, 2 min) and the pellets washed three times with immunoprecipitation buffer. Pellets were boiled with an equal volume of sample buffer. Protein concentrations were determined using Bio-Rad Bradford protein assays using bovine serum albumin (BSA) protein standards.

### Assessment of MAP4K4 by mass spectrometry

Twelve 60 mm dishes of cardiomyocytes were infected with adenoviruses for expression of WT-MAP4K4 at the time of plating. After 48 h, cells (24 × 10^6^ cells per condition) were treated with/without CalA (100 nM, 20 min), then washed with 3 × 2 mls PBS and scraped into 100 µl immunoprecipitation buffer containing protease and phosphatase inhibitors. Extracts were homogenised briefly, then kept on ice (15 min) before centrifugation (12 000×***g***, 5 min). The supernatants from each treatment were pooled and 45 µl boiled with 15 µl of sample buffer. The remainder was incubated with 30 µl of a 1 : 1 slurry of EZ-view FLAG resin (Sigma) equilibrated with immunoprecipitation buffer (4°C, overnight with rotation). The samples were centrifuged (12 000×***g***, 2 min) and the pellets washed five times with immunoprecipitation buffer. Pellets were boiled with 80 µl of sample buffer. Proteins were separated by SDS–PAGE using 8% (w/v) polyacrylamide gels (200 V, 90 min). The gel was stained with Coomassie blue stain (1 h, room temperature), destained overnight, and stored in 45% (v/v) methanol. The major bands were excised from the gel and sent to the Fingerprints Proteomics Facility, College of Life Sciences, University of Dundee for phosphoprotein analysis and protein identification. The data summary and primary output data are in Supplemental Spreadsheets S1–7.

Proteins were subjected to in-gel reduction with dithiothreitol and alkylation with iodoacetamide, prior to overnight (16 h) trypsin digestion (Modified Sequencing Grade, Roche). Peptides were extracted from the gel and dried in a SpeedVac (Thermo Scientific) before resuspending in 50 µl 1% (v/v) formic acid. Samples were centrifuged and the supernatants transferred to HPLC vials. Peptides were analysed by nLC–MS/MS using an LTQ Velos Pro Orbitrap (15 µl injection volume). Data analysis used Mascot for protein identification and phospho-site localisation.

### Immunoblotting and immunostaining

Proteins were separated by SDS–PAGE on 8% (w/v) polyacrylamide resolving gels with 6% stacking gels, and transferred electrophoretically to nitrocellulose as previously described [19]. For some immunoblots of MAP4K4, electrophoresis was performed for 90 min to increase separation of higher molecular mass proteins. Proteins were detected as previously described [19]. Primary antibodies for the FLAG epitope were from Sigma–Aldrich (F7425; rabbit polyclonal antibodies, 1/1000 dilution) or Santa Cruz Biotechnology Inc. (sc-166355; mouse monoclonal antibodies, 1/400 dilution). MAP4K4 antibodies (HGK) were from Cell Signaling Technologies Inc. (1/750 dilution): HGK(D19H10) rabbit monoclonal antibodies (Cat. No.: 5146) were used to assess MAP4K4 associated with striatins and HGK rabbit polyclonal antibodies (Cat. No.: 3485) were used for other immunoblots. Antibodies to phosphorylated and total JNKs (Cat. Nos.: 9251 and 9252, respectively), Gapdh (Cat. No.: 2118) and CREB (Cat. No.: 9197) were from Cell Signaling Technologies Inc. and used at 1/1000 dilution. Horseradish peroxidase-conjugated secondary antibodies (1/5000 dilution) were from Dako (supplied by Agilent). Bands were detected by enhanced chemiluminescence using ECL Prime Western Blotting detection reagents (GE Healthcare) with visualisation using an ImageQuant LAS4000 system (GE Healthcare). ImageQuant TL 8.1 software (GE Healthcare) was used for densitometric analysis. Values for all samples were normalised to the mean of the controls.

For immunostaining, cardiomyocytes were washed and fixed in 3.7% (v/v) formaldehyde in PBS and immunostained with antibodies to troponin T or with antibodies to the FLAG epitope as previously described [19,23]. Cells were viewed with a Zeiss Axioskop fluorescence microscope using a 40× objective. Digital images were captured using a Canon PowerShot G3 camera. Images were cropped for presentation using Adobe Photoshop CC 2019. Cell surface areas were measured using ImageJ, measuring a minimum of 50 cells per condition for each experiment from four independent cardiomyocyte preparations.

### MAP4K4 dephosphorylation and protein kinase assays

MAP4K4 dephosphorylation studies with PP2A were conducted as previously described [23]. Briefly, WT-MAP4K4-HEK293 were cultured in 100 mm dishes and MAP4K4 was immunoprecipitated as described above. Pellets were washed in immunoprecipitation buffer and then with PP2A buffer (50 mM TrisHCl pH 7.0, 0.15 mM ethylene glycol-bis(β-aminoethyl ether)-N,N,N′,N′-tetraacetic acid, 0.1% (v/v) 2-mercaptoethanol, 1 mg/ml BSA) before resuspending in PP2A buffer without or with 10 U/ml PP2A (catalytic subunit; Cayman chemicals) and without or with 5 µM okadaic acid. Samples were incubated for up to 30 min at 30°C. The reaction was stopped by the addition of okadaic acid (5 µM final concentration). Immunoprecipitates were washed with PP2A buffer then extracted in sample buffer diluted 1 : 1 with PP2A buffer.

For protein kinase assays, MAP4K4 was immunoprecipitated as described above and assays conducted as previously described [23], using myelin basic protein (MBP) as substrate.

### Model of the structure of the kinase domain

The crystal structure for MAP4K4 was accessed from the protein database (PDB: 4ZK5) and assessed for key structural regions using CCP4mg [24]. Images were rendered in the CCP4mg program and annotated using Adobe Illustrator.

### Modelling the coiled-coil domains in MAP4K4 with striatins

Initial tertiary structure predictions were generated for each sequence using the IntFOLD server [25] with integrated model quality scores from ModFOLD [26]. Quaternary structure predictions were generated by free docking methods, combining the output of MEGADOCK [27], FRODOCK [28], PatchDock [29] and ZDOCK [30]. The final structures were ranked for submission using our newly developed ModFOLDdock method. This protocol uses a hybrid consensus approach for producing global and local (interface residue) scores for predicted quaternary structures. The ModFOLDdock global score was taken as the mean score from five individual methods: ProQDock [31], QSscoreJury, DockQJury, VoroMQA [32] and ModFOLDIA. For each interacting pair of chains in a modelled complex, the ProQDock scores were averaged to produce a global score for the complete assembly. For the QSscoreJury and DockQJury methods, pairwise comparisons were made for each quaternary structure model to every other model made for the target and then the mean QS [33] and DockQ [34] scores were calculated. The ModFOLDIA method also carries out structure-based comparisons of alternative oligomer models and can produce both global and local/per-residue interface scores. The first stage of the ModFOLDIA method was to identify the interface residues in the model to be scored (defined as ≤5 Å between the heavy atoms in different chains). and then obtain the minimum contact distance (*Dmin*) for each contacting residue. The second stage was to locate the equivalent residues in all other models and then obtain the mean minimum distances of those residues in all other models (*MeanDmin*). The final IA score for each of the interface residues in the model was the absolute difference in the *Si* from the mean *Si* : *IA = 1−|Si-MeanSi|*, where *Si = 1/(1+(Dmin/20)2)* and *MeanSi = 1/(1+(MeanDmin/20)2)*. The global ModFOLDIA score for a model was then taken as the total interface score (sum of residue scores) normalised by the maximum of either the number of residues in the interface or the mean number of interface residues across all models for the same target.

### Statistical analysis

Data analysis used Microsoft Excel and GraphPad Prism 9. Statistical analysis was performed using GraphPad Prism 9 with two-tailed unpaired or paired/repeated measures *t*-tests, one-way or two-way ANOVA as indicated in the Figure Legends. A multiple comparison test was used in combination with ANOVA as indicated in the Figure Legends. Graphs were plotted with GraphPad Prism 9. Specific *P* values are provided with significance levels of *P* < 0.05 in bold type.

## Results

### Cloning of rat MAP4K4 and expression in rat cardiomyocytes

**Figure 1.**
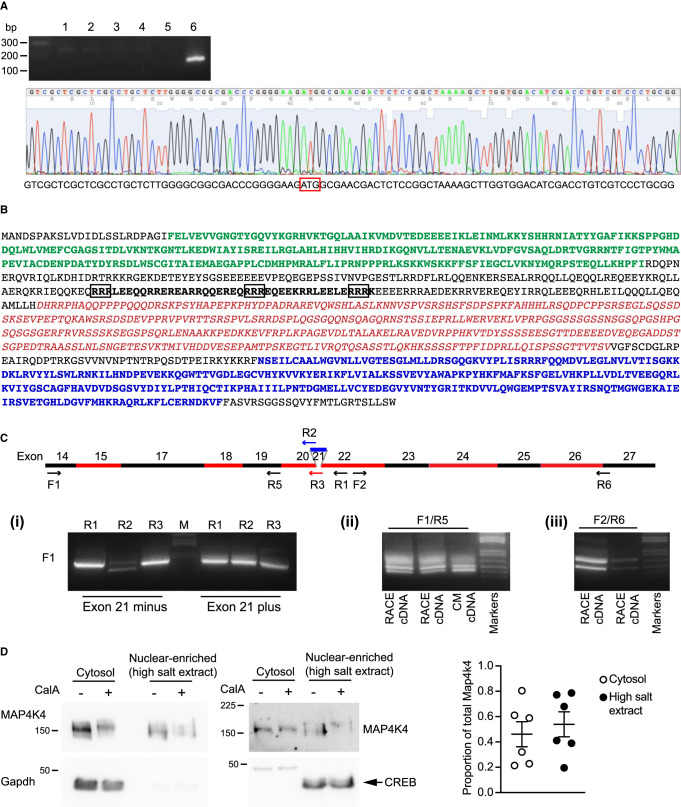
Characterisation of rat MAP4K4. (**A**) The predicted first exon and start codon for the rat MAP4K4 gene were identified by 5′-RACE using rat heart mRNA. The amplified product was cloned into a pDRIVE vector used to transform DH5α cells. Clones were screened by PCR using a primer from the 3′ end of the first exon (*upper panel*; individual clones numbered 1–6). Clone 6 was sequenced (*lower panel*) and contained the start codon (red box) plus upstream non-coding sequence. (**B**) The predicted protein-coding sequence of rat MAP4K4 cloned from neonatal rat cardiomyocytes is shown, identifying the kinase domain (green), the citron homology domain (blue), the region subject to alternative splicing (exons 15–25; red italics) and the region containing a predicted nuclear-localisation signal (black, bold type; key residues identified by NucPred outlined in black). (**C**) Evidence for alternatively spliced MAP4K4 mRNAs in rat cardiomyocytes with a schematic of the spliced exons and positions of PCR primers (*upper panel*; F, forward primer; R, reverse primer). (**i**) cDNAs were cloned from myocytes with and without exon 21 (identified by DNA sequencing). This was confirmed using primers spanning the exon boundary. Primers F1 and R5 were used for PCR to amplify MAP4K4 sequences between exons 14 and 19 (**ii**) and primers F2 and R6 were used to amplify MAP4K4 sequences between exons 22 and 26 (**iii**). Multiple bands were detected in cDNAs prepared for RACE and from cardiomyocyte (CM) RNA, consistent with alternative splicing across the region. (**D**) Cardiomyocyte MAP4K4 is expressed in the cytosol and in high salt extracts, enriched for nuclear proteins. Cardiomyocyte proteins (cytosol: 0.2 × 10^6^ cells; nuclear-enriched high salt extracts: 0.4 × 10^6^ cells) were immunoblotted for MAP4K4 (8% (w/v) polyacrylamide gels, electrophoresis: 60 min, 200 V; upper immunoblots), Gapdh (10% (w/v) polyacrylamide gels; lower left immunoblot) or CREB (10% (w/v) polyacrylamide gels; lower right immunoblot). The assessment was conducted on 6 occasions with independent cardiomyocyte preparations. Although MAP4K4 was routinely detected in the high salt extracts, there was variation and blots show the experiment with the least amount of MAP4K4 detected in this fraction (*left panels)*. Densitometric analysis of each of the experiments is provided (*right panel*). The proportion of MAP4K4 was calculated on a per cell basis, allowing for the volume of extracts loaded onto the gel (10 µl for each lane for the blots shown) relative to the total volumes of each of the cytosol (200 µl) and nuclear-enriched high salt extracts (70 µl) containing proteins from 4 × 10^6^ cells. Individual data points are shown with means ± SEM (*n* = 6).

Cardiac research relies on the rat as an experimental model. Human and mouse MAP4K4 genes are well-defined, but the gene structure for rat has been provisional with a predicted mRNA sequence (NM_001106904.1) differing from human and mouse at the 5′ end. The predicted protein (NP_001100374.1) initiated within that of the human and mouse proteins, lacking part of the kinase domain (Supplemental Figure S1). We aligned the provisional rat gene with the longest potential isoforms for human and mouse to identify a putative 5′ exon for the rat gene containing the start codon. To confirm this, we used 5′-RACE with adult rat heart mRNA, cloning the products and sequencing the DNA from the 3′ end of the predicted first coding exon (Figure 1A). Full-length rat MAP4K4 was cloned from neonatal rat cardiomyocyte RNA, sequenced and aligned with the rat genome (Supplemental Figure S2). The sequence is identical with predicted transcript variant X22 (XM_008767015) (Supplemental Figure S3), encoding a predicted 1233 amino acid protein (Figure 1B; Supplemental Figure S4) homologous to the originally identified mouse sequence [1]. Compared with the longest potential isoform (predicted molecular mass 158.6 kDa), the isoform cloned lacks exons 16 and 34, along with 5′ and 3′ extensions for exons 15 and 30, respectively (predicted molecular mass 140.8 kDa).

Wright et al.[16] identified multiple alternatively spliced transcripts of MAP4K4 in human tumour cells, with nine modules (M1–M9) between exons 14 and 30 subject to alternative splicing. We cloned rat cardiomyocyte MAP4K4 with and without exon 21 (9 bp) encoding a tripeptide, GEV (M6 in Wright et al. [16]). This was confirmed by PCR using primers across the exon boundary (Figure 1C(i)). In further experiments, we assessed splicing across exons 14–22, identifying multiple PCR products indicative of alternative splicing (Figure 1C(ii)). PCR amplication across exons 22 to 26 also identified multiple transcripts (Figure 1C(iii)). PCR amplification across exons 13 and 31 identified a novel sequence (KM217582) corresponding to a different isoform lacking exons 17 and 21, but containing the 5′ extension of exon 15. Overall, we conclude that, as in tumour cells [16], cardiac MAP4K4 is expressed as multiple alternatively spliced transcripts with alternative splicing concentrated between exons 14 and 26.

**Figure 2.**
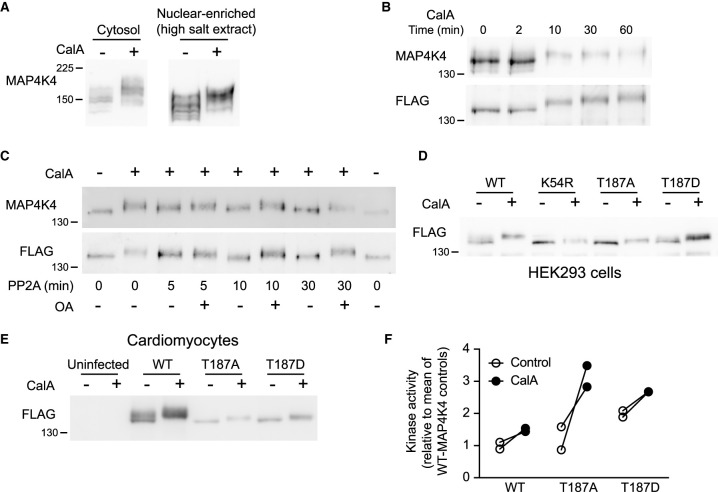
Inhibition of Ser/Thr protein phosphatase(s) with calyculin A activates and promotes hyperphosphorylation of cardiomyocyte MAP4K4. (**A**) Cardiomyocytes were treated without/with calyculin A (CalA; 200 nM, 10 min) and proteins (cytosol: 0.4 × 10^6^ cells; nuclear-enriched high salt extracts: 1 × 10^6^ cells) were immunoblotted for endogenous MAP4K4 (8% (w/v) polyacrylamide gels; electrophoresis: 90 min, 200 V). Immunoblots are representative of at least three independent preparations of cardiomyocytes. (**B**) HEK293 cells with stable expression of FLAG-tagged wild-type (WT) MAP4K4 (WT-MAP4K4-HEK293 cells) were treated without or with 200 nM CalA for the times shown and samples (50 µg) were immunoblotted for MAP4K4 (*upper image*) or the FLAG epitope (*lower image*). (**C**) WT-MAP4K4-HEK293 cells were treated with CalA (200 nM, 10 min) and MAP4K4 immunoprecipitated using FLAG antibodies. Immunoprecipitates were incubated with PP2A for the indicated times, in the absence or presence of 5 µM okadaic acid (OA). The dephosphorylation reaction was stopped by addition of OA, and samples immunoblotted for MAP4K4 (*upper image*) or the FLAG epitope (*lower image*). (**D**,**E**) HEK293 cells were transfected (**D**) or cardiomyocytes infected with adenoviruses (**E**) for expression of FLAG-tagged WT-MAP4K4 or MAP4K4 containing mutations or deletions as indicated. Cell extracts were immunoblotted (50 µg for HEK293 cells; 25 µg for cardiomyocytes) for the FLAG epitope (**D** and **E**). (**F**) Cardiomyocytes were infected with adenoviruses for expression of FLAG-tagged WT-MAP4K4, MAP4K4(T187A) or MAP4K4(T187D). Following immunoprecipitation using anti-FLAG antibodies, kinase activities were assayed using myelin basic protein as substrate and γ[^32^P]-ATP. Kinase activities were calculated by subtracting the cpm for uninfected cells from cpm for infected cells, and adjusting the MAP4K4(T187A) and MAP4K4(T187D) activities to account for the lower relative level of expression compared with WT-MAP4K4. Results are expressed relative to the means of control WT-MAPK4 with individual values shown for each of two independent cardiomyocyte preparations. For **E** and **F**, 2 × 10^6^ cardiomyocytes were infected with 1 × 10^7^ infectious units/ml of each of the adenoviruses (i.e. multiplicity of infection = 5).

Analysis of MAP4K4 protein sequence using NLStradamus [35] predicted a nuclear localisation signal in residues 395–460 (Viterbi Path analysis) or 413–454 (Posterior analysis with 0.9 confidence). NucPred [36] also predicted a split nuclear localisation signal in residues 401–450 with high confidence (0.98 score; Figure 1B). Analysis by ProtComp 9.0 (Softberry Inc.) suggested MAP4K4 is located in both cytoplasmic and nuclear compartments. We could not detect MAP4K4 in cardiomyocytes by immunostaining so, to gain insight into MAP4K4 localisation, we used subcellular fractionation followed by immunoblotting. Biochemical separation of cytoplasmic and nuclear proteins generally relies on hypotonic cell lysis to release cytosolic proteins followed by extraction with high salt to enrich nuclear proteins [21]. Cardiomyocyte MAP4K4 was detected as multiple bands (consistent with expression of multiple alternatively spliced isoforms) with an apparent relative molecular mass >150 kDa, and was routinely detected in both cytosolic and high-salt extracts (Figures 1D and 2A). The relative proportion in the high-salt extracts was variable (Figure 1D, *right panel*). This may reflect the precise stage of post-natal development of the neonates used for the cardiomyocyte preparations (2–4 days) given the radical remodelling that occurs in the post-natal heart to accommodate the increase in workload [37].

### Inhibition of protein phosphatase activity with calyculin A activates and promotes hyperphosphorylation of cardiomyocyte MAP4K4

In other cells, MAP4K4 is regulated via STRIPAK complexes in which PP2A maintains the kinase in a dephosphorylated, inactive form [13–15]. Inhibition of Ser/Thr protein phosphatase activities with the cell-permeable inhibitor calyculin A [38] (CalA; 200 nM, 10 min) induced the appearance of bands with reduced mobility (i.e. band-shift) on immunoblots of cytosolic or nuclear-enriched cardiomyocyte extracts (Figure 2A).

To assess the degree of the band-shift, we generated HEK293 cells with stable expression of the 1233 amino acid form of wild-type (WT) FLAG-tagged MAP4K4 cloned from rat cardiomyocyte cDNA (WT-MAP4K4-HEK293 cells). These cells expressed a band of ∼150 kDa detected with antibodies to either MAP4K4 or the FLAG tag (Figure 2B). In contrast with cardiomyocytes, treatment of HEK293 cells with CalA caused the cells to detach from the tissue culture dish from ∼10 min, so cells treated with CalA were collected by centrifugation prior to protein extraction. CalA induced an increase in apparent molecular mass of ∼11 kDa. To confirm the band shift resulted from increased phosphorylation, extracts from WT-MAP4K4-HEK293 cells treated with CalA were incubated with PP2A catalytic subunit *in vitro*. This eliminated the band-shift, an effect which was negated by co-incubation with okadaic acid (an alternative PP2A inhibitor) (Figure 2C). We concluded that the MAP4K4 band-shift resulted from increased Ser/Thr phosphorylation rather than reflecting any increase in phosphorylation of Tyr residues or another post-translational modification.

To determine if CalA-induced phosphorylation of MAP4K4 required MAP4K4 kinase activity, we generated constructs for expression of kinase-dead forms of MAP4K4, with mutations of Thr187 (in the activation loop) to Ala [MAP4K4(T187A)], and Lys54 to Arg [MAP4K4(K54R)] as in [2]. We also mutated Thr187 to Asp [MAP4K4(T187D)] to generate a form predicted to have increased basal activity. HEK293 cells were transfected with these constructs or with constructs for WT-MAP4K4 and samples immunoblotted for the FLAG epitope. MAP4K4(T187D) migrated with a slightly higher relative molecular mass than MAP4K4(T187A) or MAP4K4(K54R) (Figure 2D). CalA induced a very minor retardation in migration of MAP4K4(T187A), MAP4K4(T187D) or MAP4K4(K54R), but not the substantial hyperphosphorylation detected with WT-MAP4K4 (Figure 2D).

To assess how MAP4K4 is regulated in cardiomyocytes and since cardiomyocytes are resistant to transfection protocols, we generated adenoviruses for expression of WT-MAP4K4 and the different mutants. Following infection and treatment, cardiomyocyte extracts were immunoblotted for the FLAG epitope (Figure 2E) or MAP4K4 immunoprecipitated for kinase activity assays (Figure 2F). Adenoviral infections of cardiomyocytes did not produce uniform expression of the different forms of MAP4K4 despite using the same multiplicity of infection (Figure 2E). The reason is not clear. However, as in HEK293 cells, CalA promoted hyperphosphorylation of WT-MAP4K4 in cardiomyocytes but only a small shift with MAP4K4(T187A) or MAP4K4(T187D) (Figure 2E). The activity of immunoprecipitated WT-MAP4K4 was increased on treatment with CalA (Figure 2F). Immunoprecipitated MAP4K4(T187A) also showed an increase in activity with CalA, possibly greater than that of WT-MAP4K4, despite being a kinase-dead mutation [2]. MAP4K4(T187D) immunoprecipitates had a higher basal activity than WT-MAP4K4 as expected, but exhibited a similar increase in activity induced by CalA. The reason(s) for these anomalies with respect to kinase activities remain to be investigated, but may relate to other activities in the protein complexes.

### MAP4K4 is phosphorylated on multiple sites in the region subject to alternative-splicing

**Figure 3.**
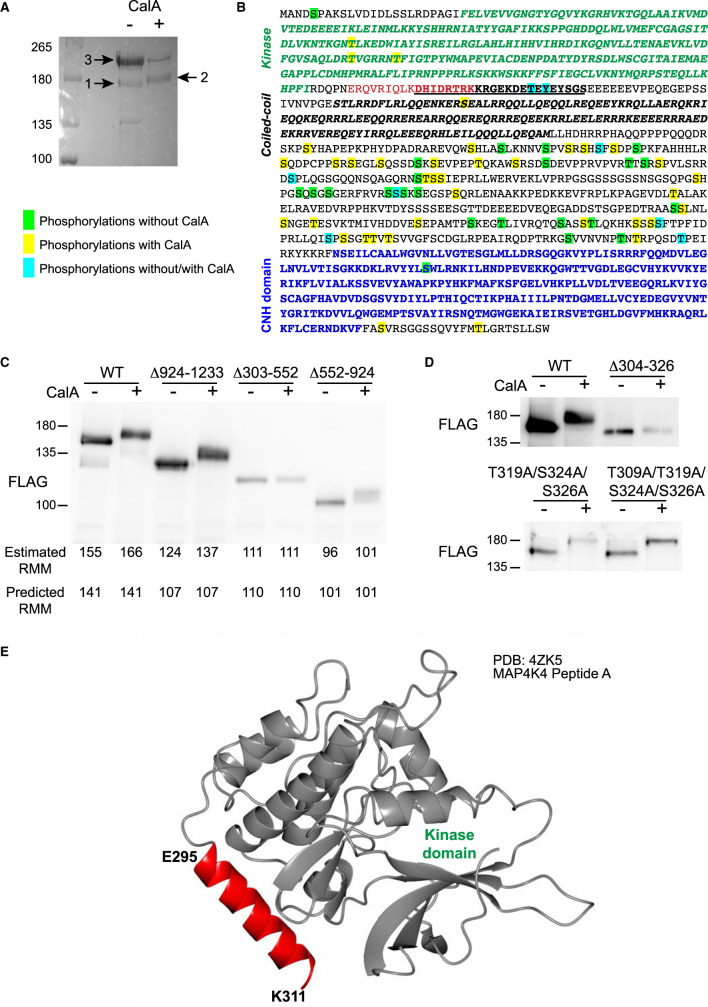
MAP4K4 phosphorylation in cardiomyocytes and deletion analysis. (**A**,**B**) Cardiomyocytes (12 × 10^6^ cells) were infected with adenoviruses for expression of wild-type MAP4K4 and treated without or with calyculin A (CalA; 200 nM, 10 min). MAP4K4 was immunoprecipitated and separated by SDS–PAGE. Gels were stained with Coomassie Brilliant Blue R (**A**). Bands 1 and 2 were excised and taken for phosphoproteomics analysis. Band 3 was taken for identification using proteomics. Similar results were obtained with at least three separate cardiomyocyte preparations. (**B**) MAP4K4 protein sequence showing the positions of phosphorylation sites in untreated cardiomyocytes (green), in cells with CalA treatment (yellow) or in untreated and treated cells (cyan). The kinase domain (green), citron homology domain (CNH, blue), the helix C-terminal to the kinase domain (E295–K311; red) and the predicted coiled-coil region (black, bold type in italics) are shown. The Δ23 region (deletion of which eliminates kinase activity) is underlined. (**C**,**D**) HEK293 cells were transfected with constructs for FLAG-tagged wild-type (WT) MAP4K4, MAP4K4 with the indicated deletions [924–1233, Δ303–552, Δ552–924, Δ304–326 (i.e. Δ23)] or point mutations (T319A/S324A/S326A, T309A/T319A/S324A/S326A). MAP4K4 was immunoprecipitated using antibodies to the FLAG epitope and immunoblotted for FLAG. (**E**) Ribbon representation of the MAP4K4 kinase region (PDB:4ZK5 peptide A), including the kinase domain (silver, residues 1–294) and additional C-terminal αhelix (red, residues 295–311).

The band-shift detected on immunoblots of MAP4K4 following CalA treatment (Figure 2) could reflect phosphorylation of a critical residue causing a major conformational change or multiple phosphorylations. We took a phosphoproteomics approach to gain insight into MAP4K4 phosphorylations. Cardiomyocytes were infected with adenoviruses encoding WT-MAP4K4 and treated without/with CalA. MAP4K4 was immunoprecipitated (Figure 3A) using antibodies to the FLAG tag, and phosphorylation sites identified by mass spectrometry (Supplemental Table S2; Supplemental Spreadsheets S1–5). Many phosphorylation sites were identified in unstimulated cells, but the overall level of phosphorylation increased with CalA treatment (Figure 3B; Supplemental Spreadsheets S1–5). CalA increased phosphorylation of the activation loop T187 consistent with activation of MAP4K4. An additional two Thr residues in the kinase domain were phosphorylated (T124 and T181) following CalA treatment, plus a Ser and Thr residue at the C terminus, but the remainder of the numerous phosphorylation sites were detected in the third quadrant of the protein (residues 513–919), coincident with the region subject to alternative splicing (Figure 1B).

Further studies were conducted in HEK293 cells transfected with constructs for MAP4K4 with various deletions and mutations. Loss of the C-terminus (Δ924–1233) had a negligible effect on the band shift induced by CalA (equivalent to an apparent increase in molecular mass of ∼13 kDa compared with ∼11 kDa) (Figure 3C). Deletion of the third quadrant (Δ552–924) substantially reduced the apparent increase in size, consistent with the identification of numerous phosphorylation sites using phosphoproteomics (Figure 3B,C; Supplemental Table S1). Mutational analysis of individual Ser/Thr residues within this region did not identify any key sites responsible for the observed band-shift following CalA treatment (data not shown). Deletion of the second quadrant (Δ304–552) immediately C-terminal to the kinase domain almost completely eliminated the band shift resulting from CalA treatment (Figure 3C). A critical 23 amino acid sequence (residues 304–326) was required for the CalA-induced band-shift (Figure 3D). Mutational analysis of all possible Ser/Thr or Tyr residues in this region did not prevent the band-shift (Figure 3D) so, although the Δ23 region is required for hyperphosphorylation, it does not account for the band shift itself. Crystal structures of MAP4K4 indicated that part of the Δ23 region lies within a helical structure C-terminal to the kinase domain, and is closely associated with it (Figure 3B,E). Presumably, this region is necessary for kinase activity.

### The coiled-coil region in MAP4K4 C-terminal to the kinase domain potentially interacts with the coiled-coil domain in striatins

**Figure 4.**
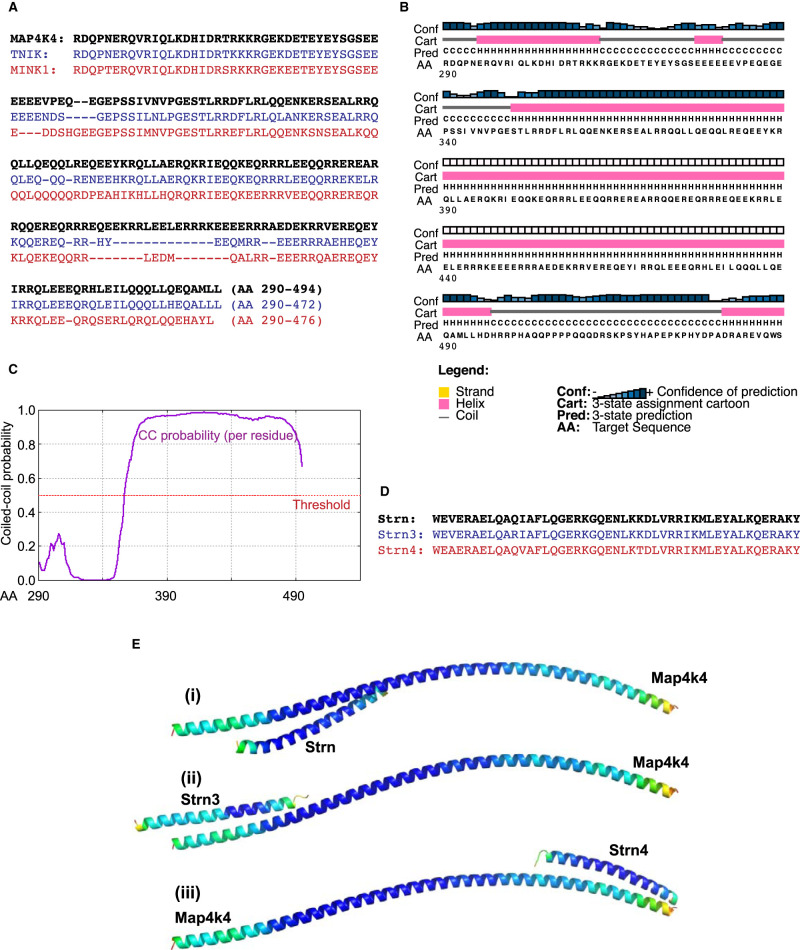
Prediction of a coiled-coil domain in MAP4K4. (**A**) Alignment of the amino acid (AA) sequences for the MAP4K4 region C-terminal to the kinase domain (AA 290–494; black, bold type) with other GCKIV family members, TNIK (blue) and MINK1 (red). (**B**) PsiPred prediction of secondary structure for MAP4K4 (290–539) indicates the presence of an extensive 147 residue sequence with helical structure between amino acids 351 and 497 (pink block). (**C**) Prediction of coiled-coil (CC) region within MAP4K4 (290–539) using DeepCoil analysis indicates high probability of CC between amino acids 351 and 490 (purple trace). (**D**) Alignment of coiled-coil region of human Strn (NP_003153.2), Strn3 (NP_001077362.1) and Strn4 (NP_037535.2). (**E**) Free docking models showing MAP4K4 association with striatins. Models are coloured by accuracy self-estimates of local quality using the temperature coloured scheme, from blue (indicating residues predicted to be close to the native structure) to red (indicating unstructured areas or residues distant from the native structure). Images were rendered using PyMOL (http://www.pymol.org/). (**i**) Strn: The association between chains covers the length of the Strn CC domain and is situated towards the N-terminus of the larger MAP4K4 helix. (**ii**) Strn3: The association covers only part of the Strn3 CC domain which is sited at the N-terminus of the larger MAP4K4 helix. (**iii**) Strn4: A much closer association covering the whole of the Strn4 CC domain but sited at the C-terminus of the MAP4K4 helix.

The GCKIV family are classified on the basis of having an N-terminal kinase domain and a C-terminal CNH domain, although a coiled-coil region has been identified [16] C-terminal to the kinase domain and the Δ23 region we identified as being critical for hyperphosphorylation (indicative of kinase activity). Residues 290–494 of MAP4K4 are highly conserved between all three GCKIV family members (Figure 4A). We used PsiPred 4.0 [39] to predict helical regions, and identified the helix corresponding to that immediately C-terminal to the kinase domain and encompassing part of the Δ23 region (Figure 3B), in addition to a more extensive region corresponding to residues 350–496 (Figure 4B) that corresponded to the coiled-coil region identified in Wright et al. [16]. This was confirmed using DeepCoil [40], indicating high probability of a coiled-coil structure (Figure 4C). Coiled-coil regions often form protein interaction domains. In non-cardiac systems, MAP4K4 interacts with Strn4 [13–15], and striatins also contain a conserved coiled-coil domain [12] (Figure 4D) so we hypothesised that the coiled-coil in MAP4K4 may be the point of interaction with one or more striatin(s). Molecular modelling indicated that the each of the striatins (Strn, Strn3 and Strn4) could potentially interact with the coiled-coil region of MAP4K4 (Figure 4E). All models were of high confidence although, despite the very high degree of amino acid identity in the coiled-coil region for the striatins (Figure 4D), Strn and Strn3 showed a higher predicted interaction with the N-terminus of the MAP4K4 coiled-coil region whereas Strn4 demonstrated a preference for the C-terminus (Figure 4E).

### Role of MAP4K4 in cardiomyocytes

**Figure 5.**
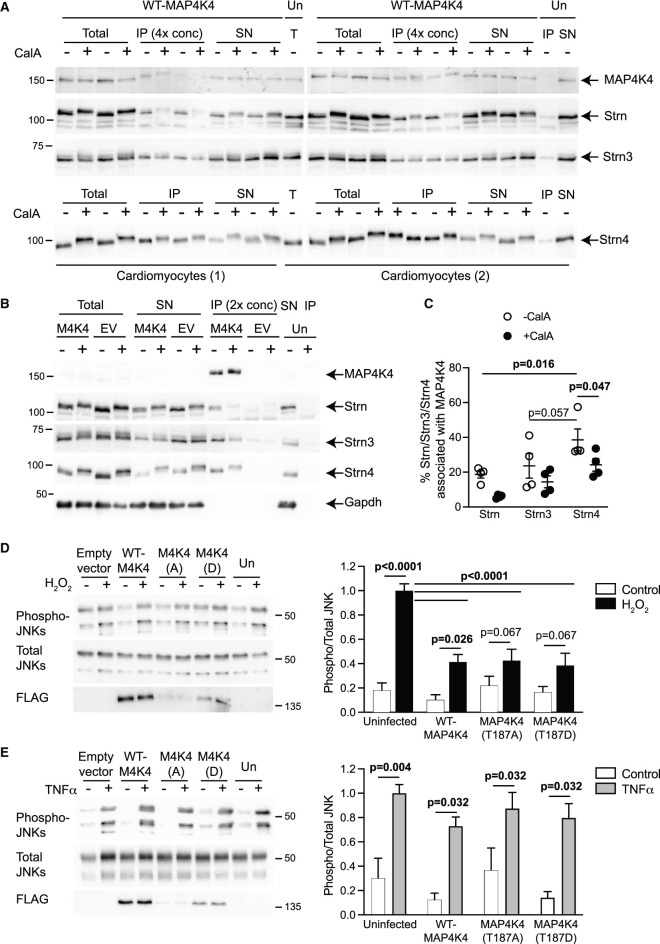
MAP4K4 associates with striatins and inhibits activation of JNKs by H_2_O_2_ in cardiomyocytes. (**A**–**C**) Cardiomyocytes were uninfected (Un), infected with empty virus (EV) for expression of FLAG alone, or infected with adenoviruses for expression of wild-type (WT) MAP4K4 (M4K4). Cells were treated with calyculin A (CalA; 200 nM, 10 min) or DMSO (1/2000 dilution) and extracts were prepared. Proteins were immunoprecipitated using anti-FLAG antibodies, and equal volumes of the total extract, immunoprecipitated proteins (IP) and supernatants (SN) were immunoblotted with antibodies to MAP4K4, Strn, Strn3 or Strn4. The concentration of proteins in the IP relative to the SN for the same cell extracts is indicated. (**A**) Data for two independent cardiomyocyte preparations (1 and 2) are shown. (**B**) One of two similar experiments is shown. (**C**) Densitometric analysis of the blots in **A** and **B** to show the percentage of each striatin associating with WT-MAP4K4. The raw densitometric values were adjusted for final volumes of the SN and the IP, and the percentage in the IP calculated relative to the total amount (SN + IP). (**D**,**E**) Cardiomyocytes were uninfected, or infected with adenoviruses containing empty vector or constructs for WT-MAP4K4 (WT-M4K4), MAP4K4(T187A) [M4K4(A)] or MAP4K4(T187D) [M4K4(D)]. Cells were treated with H_2_O_2_ (1 mM, 15 min; **D**) or TNFα (25 ng/ml, 15 min; **E**) and extracts (25 µg) immunoblotted with antibodies for phosphorylated (phospho-) or total JNKs, or the FLAG epitope. Representative blots are on the left (positions of relative molecular mass markers are indicated on the right of each blot) with densitometric analysis on the right. Results are means ± S.E.M (*n* = 5 independent cardiomyocyte preparations). Individual *P* values are given (repeated measures two-way ANOVA with Holm–Sidak post-test).

Since MAP4K4 interacts with Strn4 in non-cardiac cells and our data predict that MAP4K4 can interact with each of the striatins (Figure 4), we assessed whether MAP4K4 interacts with any or all of the striatins in cardiomyocytes. Adenoviruses were used to overexpress WT-MAP4K4 in cardiomyocytes treated without/with CalA, and association with each of the striatins was assessed following immunoprecipitation of MAP4K4 with anti-FLAG antibodies. Endogenous Strn, Strn3 and Strn4 were all detected in MAP4K4 immunoprecipitates, although there was significantly higher association of Strn4 than Strn itself and reduced association on CalA-treatment (Figure 5A–C). CalA promoted a clear band-shift in Strn and Strn4, suggesting these proteins are subject to regulation by reversible phosphorylation.

**Figure 6.**
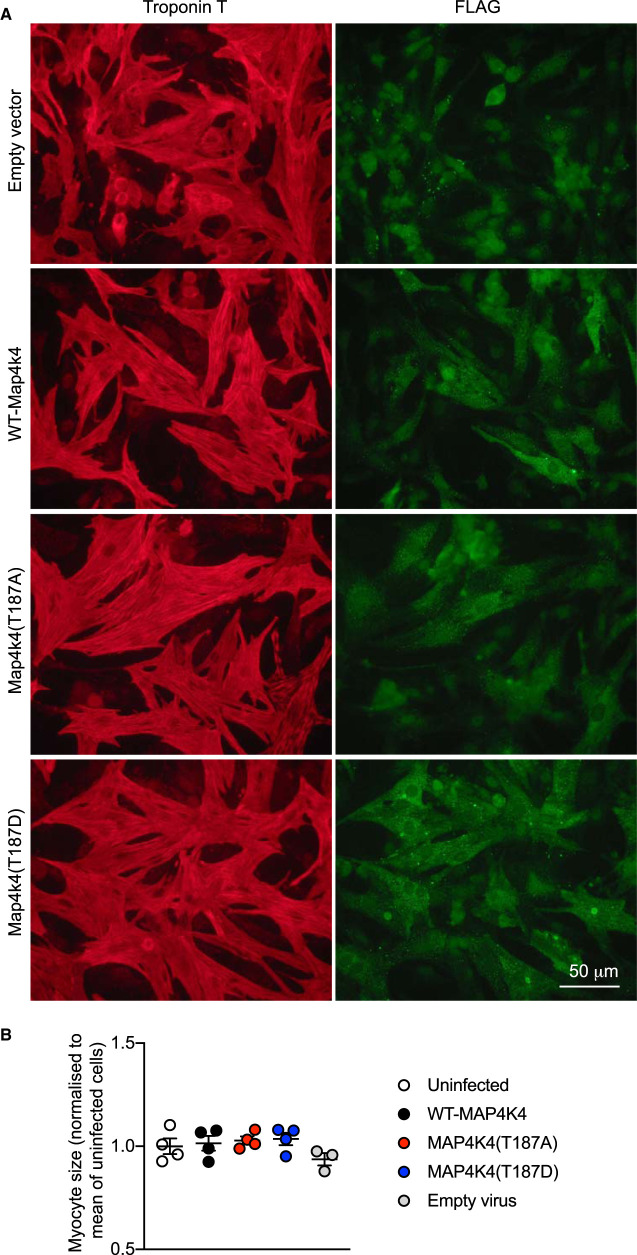
Overexpression of MAP4K4 enhances cardiomyocyte myofibrillar organisation. Cardiomyocytes were uninfected, or infected with adenoviruses containing constructs for WT-MAP4K4, MAP4K4(T187A) or MAP4K4(T187D). Serum was withdrawn and cells were incubated for 48 h in serum-free medium. (**A**) Cells were immunostained with antibodies to the FLAG epitope (*right panels*, green) or troponin T (*left panels*, red). (**B**) Cardiomyocyte surface area was measured using ImageJ. Results are shown for four independent cardiomyocyte preparations. Mean values for each experiment (calculated from a minimum of 50 cells per condition) are plotted, together with the overall means ± S.E.M.

Other studies have indicated that MAP4K4 is activated in the heart by oxidative stresses and this activates JNKs to promote stem-cell derived ‘cardiomyocyte' apoptosis [7], whilst studies in other cells implicate it in activation of JNKs by TNFα [41]. However, activation of JNKs by MAP4K4 is not a universal finding [42–44]. We therefore expressed WT-MAP4K4, MAP4K4(T187A) and MAP4K4(T187D) in cardiomyocytes and assessed activation of JNKs by immunoblotting for the phosphorylated (i.e. activated) enzymes. In our hands, overexpression of MAP4K4 did not affect the basal phosphorylation of JNK and, rather than activating JNKs, MAP4K4 overexpression inhibited JNK phosphorylation in cardiomyocytes treated with H_2_O_2_ (as a physiologically relevant oxidative stress) (Figure 5D). Activation of JNKs by TNFα may be reduced to some degree by WT-MAP4K4 or MAP4K4(T187D), but to a lesser extent than with H_2_O_2_ (Figure 5E). In other studies (data not shown), we failed to detect hyperphosphorylation of either endogenous or overexpressed MAP4K4 in cardiomyocytes treated with H_2_O_2_ or TNFα, nor did we see any significant increase in activity following immunoprecipitation of overexpressed WT-MAP4K4. We next determined the effects of overexpressing MAP4K4 in cardiomyocytes on morphology. Immunostaining for the FLAG epitope demonstrated punctate staining for MAP4K4 throughout the cytoplasmic compartment with apparently less in the nucleus of some cells (Figure 6, *right panels*, green). Overexpression of WT-MAP4K4 did not increase cardiomyocyte death, but increased myofibrillar organisation as shown by troponin T immunostaining (Figure 6A, *left panels*, red). Overexpression of MAP4K4(T187A) and MAP4K4(T187D) (Figure 2F) appeared to enhance myofibrillar organisation (Figure 6A, *left panels*), suggesting that the effect on myofibrillar organisation may be kinase independent. There was no significant change in average cardiomyocyte cell surface area (Figure 6B).

The punctate staining for FLAG-MAP4K4 (Figure 6, *right panels*) suggested that it may be associated with other proteins in discrete regions. Our data indicate that MAP4K4 associates with striatins which potentially tethers the kinase, but we also detected a high-molecular mass MAP4K4-interacting protein in MAP4K4 immunoprecipitated from untreated cardiomyocytes (Band 3; Figure 3A). Proteomics analysis of this band identified it as containing myosin heavy chain isoforms, including cardiac muscle Myh6 and Myh7, the non-muscle myosin Myh9, and smooth muscle myosin Myh11 (Supplemental Table S3; Supplemental Spreadsheets S1, S6 and S7). Fragments of MAP4K4 (particularly corresponding to the unstructured linker region between the predicted coiled-coil domain and the CNH domain plus the CNH domain itself) were also identified in this band (Supplemental Table S4), further supporting the concept of a specific interaction. Interaction with myosin heavy chains could account for the punctate staining observed. Association of MAP4K4 with myosin heavy chain was lost following treatment of cardiomyocytes with CalA (Figure 3A), possibly a consequence of phosphorylation of MAP4K4 and/or myosin.

**Figure 7.**
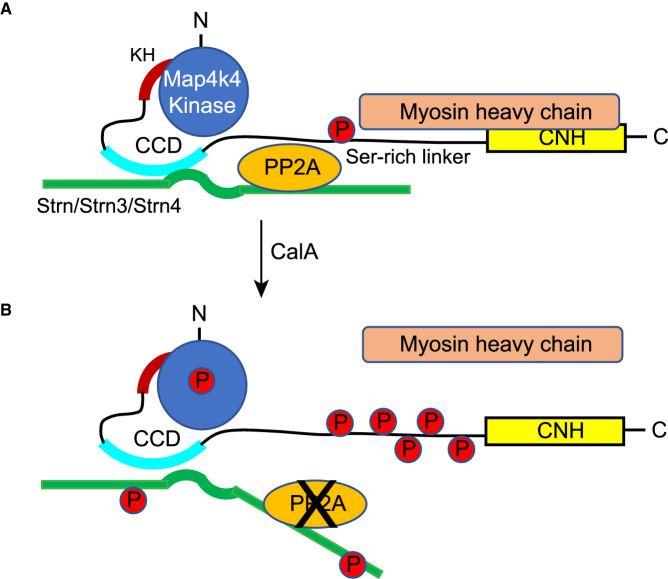
Schematic diagram of the conclusions from this study. MAP4K4 associated with one or more striatins in cardiomyocytes, potentially through a coiled-coil domain (CCD) in MAP4K4(351–495). We identified an additional helix structure (KH), C-terminal to the kinase domain, deletion of which eliminated the hyperphosphorylation associated with kinase activity. In unstimulated cells, MAP4K4 associates with myosin heavy chain, and the STRIPAK complex maintains the kinase in an inactive state (**A**). Inhibition of PP2A (e.g. with CalA) results in activation of MAP4K4 (**B**), with phosphorylation of the activation loop residue (T187) and hyperphosphorylation of the unstructured linker region between the CCD and the C-terminal citron homology (CNH) domain. Strn and Strn4 probably also become phosphorylated. Following treatment with CalA, the association of MAP4K4 with myosin heavy chain is lost.

Overall, as shown in Figure 7, our studies indicated that MAP4K4 associates with one or more of the three striatins in cardiomyocytes (Figure 5A–C), potentially through the coiled-coil domain in MAP4K4(351–495) (Figure 4). We also identified an additional helix structure C-terminal to the N-terminal kinase required for kinase activity (Figure 3D,E). In unstimulated cardiomyocytes, MAP4K4 associates with myosin heavy chain (Figure 3A), and the STRIPAK complex maintains the kinase in an inactive state (Figure 7A). Inhibition of PP2A (e.g. with CalA) results in activation of MAP4K4, with phosphorylation of the activation loop residue (T187) and hyperphosphorylation of the unstructured linker region between the coiled-coil domain and the CNH domain. Strn and Strn4 probably also become phosphorylated. On activation and hyperphosphorylation of MAP4K4, the association with myosin heavy chain is lost (Figure 7B).

## Discussion

MAP4K4 is emerging as a key regulatory kinase involved in the development of major diseases in society including cardiovascular disease and cancer [4,5]. However, details of its regulation and specific function remain to be fully elucidated. With respect to its regulation, despite reports suggesting MAP4K4 acts as an intermediate signalling component [2,7], it now seems much more probable that it is regulated in the context of STRIPAK complexes where a striatin tethers the kinase in the vicinity of PP2A which maintains it in a dephosphorylated and inactive state [13–15]. Our data consolidate this latter view, demonstrating that MAP4K4 interacts with striatins in cardiomyocytes (Figure 5A–C), becoming activated and hyperphosphorylated when cells are treated with CalA (Figures 2 and 3). CalA inhibits PP1 (and other protein phosphatases) in addition to PP2A [38] but, in the context of MAP4K4 association with striatins, its effects seem highly likely to be mediated via inhibition of PP2A. Previous studies indicated that MAP4K4 associates specifically with Strn4 [13–15]. Our data suggest it can interact with any of the three striatins, although there may be a preference for Strn4 (Figure 5A–C). Like others, we used an overexpression approach to express MAP4K4 with an epitope-tag in order to facilitate immunoprecipitation and this does have drawbacks in that the stoichiometry of interacting proteins is altered. However, the level of overexpression was not excessive, and we infected the cells at the time of plating to facilitate interaction with endogenous proteins, so it seems likely that MAP4K4 can indeed interact with all three striatins in cardiomyocytes.

STRIPAKs are multimeric complexes containing many different proteins [12,45]. The interaction of MAP4K4 with striatins therefore raises the question of whether this is direct or mediated via another protein in the complex. *In vitro* assays previously indicated that MAP4K4, MINK1 and TNIK all interact directly with Strn4, with MINK1 (and potentially the other GCKIVs) association being mediated via its kinase and/or CNH domains, and little interaction of the intermediate linker [15]. However, it is not absolutely clear which sections were assessed in these experiments and the disordered nature of the linker region may have reduced the potential for interaction. We hypothesised that the point of interaction was most likely to be via the coiled-coil domains conserved in the GCKIVs and in the striatins, and computational modelling of these structures supports this view (Figure 4E). Other GCKs are regulated in STRIPAK complexes including the GCKIIs (e.g. MST1/MST2 of the Hippo signalling pathway) and GCKIIIs (e.g. MST3) [12,45], and it remains to be established if all GCK family members are regulated in a similar way. The interaction of MST3 with striatin appears to be indirect and mediated via CCM3 [46], whereas the interaction of MST1/MST2 with striatin requires another protein, SLMAP (sarcolemmal membrane–associated protein) [47]. It is therefore possible that other proteins are required for interaction of MAP4K4 with striatins, whether as a direct mediator or in a facilitatory capacity. Clearly, further experiments are necessary to determine if other proteins are required for MAP4K4 interaction with striatins and establish if there are differences in association between the three GCKIVs and the three striatins.

In the course of this work, a cryo-EM structure of a STRIPAK complex was published [48]. This was generated from expressed proteins with Strn3 at its core and containing PP2AA, PP2AC, STRIP1 and MOB1. The complex contained four copies of Strn3 and a single copy of each of the other proteins, with the coiled-coil region forming the scaffold. The complex did not contain a kinase or many of the other proteins known to be found in STRIPAK complexes [12,45] and, although the structure interacts with a complex of STK25 (a GCKIII) and CCM3, the points of association were not established. There could be direct interaction of kinases such as MAP4K4 with the coiled-coil domains at the heart of the structure. However, it should perhaps also be considered that different STRIPAK complexes are likely to be variations on a theme, with the three different striatins interacting with different proteins with various structural constraints.

Reversible protein phosphorylation is a key regulatory mechanism that can alter enzyme activities or modulate protein-protein interactions. In MAP4K4, inhibition of PP2A with CalA activates MAP4K4 with phosphorylation of the activation loop residue, T187 (Figure 3B), as identified previously [2]. MAP4K4(T187D) had a higher basal activity than WT-MAP4K4 but, surprisingly, the T187A mutation produced a kinase with potentially higher activity in immunoprecipitation kinase assays *in vitro* following CalA-treatment, whilst MAP4K4(T187D) showed a similar increase in activity as seen with WT-MAP4K4 (Figure 2F). The reasons are not clear. However, the expressed proteins are clearly integrated in kinase signalling complexes in cardiomyocytes, as seen by the interaction of WT-MAP4K4 with striatins (Figure 5A–C). It is likely that other proteins associated with the STRIPAK complexes are present in the immunoprecipitates and these may influence the activities in the type of assay we used. Further studies with different types of assay are clearly necessary to assess this. In addition to activation loop phosphorylation, MAP4K4 became hyperphosphorylated following treatment with CalA (Figures 2 and 3). MAP4K4 was already phosphorylated in untreated cells, with phosphorylations concentrated in the linker domain between the coiled-coil region and the CNH domain (Figure 3B), and this potentially contributed to the anomalous migration of the protein with SDS–PAGE (∼14 kDa greater than the predicted relative molecular mass). CalA increased phosphorylation of the linker region with a further increase in apparent molecular mass (∼11 kDa). MAP4K4 kinase activity was required for hyperphosphorylation, since MAP4K4(T187A) and MAP4K4(T187D) did not become hyperphosphorylated in response to CalA (Figure 2D,E). Further studies with deletion mutants and mutation of each of the various Ser/Thr residues (Figure 3B,C; data not shown) did not identify any other residues as being crucial for the band shift to occur. Phosphorylation of one or more of the Ser/Thr residues in the linker region may modulate specific interactions with other proteins (as in Mst3 [23]). Alternatively, such global phosphorylation with the increased negative charge may serve to extend the linker forming a more rod-shaped structure. There appear to be fewer constraints on the primary sequence in this region, with a high degree of alternative splicing (Figure 1B,C) and poor conservation between the three GCKIVs, also suggesting the overall length/structure may be more important than the specific sequence and phosphorylation sites.

With respect to function, MAP4K4 was originally identified as an upstream kinase of the JNK cascade, interacting with MEKK1 or TAK1 to signal via MKK4/7 to JNKs [1,2]. Although others have reported that MAP4K4 is activated by oxidative stresses in the heart and signals to JNKs [7], our data are not consistent with this: overexpression of WT-MAP4K4 did not increase basal JNK activity in cardiomyocytes, and inhibited activation of JNKs by H_2_O_2_ (Figure 5D,E). MAP4K4(T187A) and MAP4K4(T187D) also inhibited activation of JNKs by H_2_O_2_, suggesting the effect was kinase-independent. Our data are more consistent with other studies in skeletal muscle that showed no role for JNKs in MAP4K4-dependent suppression of differentiation [43], that do not show activation of JNKs by overexpression of MAP4K4 [42], and which do not implicate MAP4K4 in activation of JNKs by TNFα [42,44]. A second functional theme that has developed for MAP4K4 is the regulation of cell adhesion and migration [9,10]. The regulation of MAP4K4 within STRIPAK complexes is more consistent with this, given that striatins are similarly implicated in regulating cell migration, albeit in association with other GCKs [12,49]. Our data also suggest that MAP4K4 regulates the cardiomyocyte cytoskeleton. Cardiomyocyte hypertrophy is associated with an increase in cell size, together with increased myofibrillar content and organisation. Expression of WT-MAP4K4 in cardiomyocytes increased the myofibrillar organisation compared with cells infected with the empty vector, although there was no obvious increase in average cell surface area (Figure 6). MAP4K4(T187A) and MAP4K4(T187D) also enhanced myofibrillar organisation and cell–cell interactions, suggesting the effect is kinase-independent and potentially a result of a scaffolding effect.

Further evidence that MAP4K4 regulates the cardiomyocyte cytoskeleton derives from the observation that myosin heavy chain immunoprecipitated with WT-MAP4K4 expressed in cardiomyocytes (Figure 3A). An initial thought was that this could be a non-specific interaction, particularly since cardiomyocytes contain such a large amount of myosin in the contractile apparatus (Myh6 and Myh7). However, although immunostaining showed a punctate distribution of MAP4K4 in the cytoplasm (Figure 6), this did not appear as striations as might be expected for general non-specific co-localisation. Furthermore, Myh9, a non-muscle myosin heavy chain was also detected in Band 3 (Figure 3A). The association of myosin heavy chain with MAP4K4 was lost following treatment with CalA and hyperphosphorylation of the kinase (Figure 3A), suggesting the interaction was specific. Our proteomics data indicated that fragments of MAP4K4 were present in the myosin heavy chain band. These fragments gave greater coverage of the unstructured linker and CNH domain (i.e. C-terminal to the coiled-coil domain) suggesting that this may be the region for interaction and the phosphorylation of the unstructured linker may be sufficient to reduce the interaction. It is possible that phosphorylation of myosin could displace MAP4K4. Alternatively or additionally, the interaction could be regulated by one or more other proteins in the complex. Here, it is interesting to note that the small G protein, Rap2 associates with the CNH domain of MAP4K4 in its activated (i.e. GTP-bound) form [17,18] and, as a molecular switch, it could influence MAP4K4 association with other proteins in the complex. Further studies will be necessary to define the MAP4K4 interactome in cardiomyocytes and establish how these interactions are modulated.

MAP4K4 is implicated in major diseases including cardiovascular/metabolic diseases, cancer, and diseases associated with neuronal degeneration [4–6,50]. Consequently, MAP4K4 kinase is viewed as a good therapeutic target. Although the emphasis has been to inhibit a potential upstream kinase in the JNK pathway to switch off pro-apoptotic signalling, perhaps equally (or more) importantly, inhibiting MAP4K4 may stabilise cell–cell and cell–matrix interactions, reducing permeability of endothelial cell barriers. However, as one of three highly conserved GCKIV kinases, it is difficult to develop a specific small molecule inhibitor for the MAP4K4 kinase domain. For example, GNE220, GNE495, DMX5804 and PF-6260933 (probably the most selective inhibitors so far identified) have IC_50_ values for MAP4K4 in the low nanomolar range, but inhibit other kinases in the submicromolar range and all inhibit the other GCKIV kinases with similar potency [7,10,51]. However, the kinase domain constitutes only ∼25% of the total protein itself. Further studies are needed of the additional α helix required for kinase activity, the coiled-coil domain plus the phosphorylation linker region and the CNH domain to increase understanding of the various protein-protein interactions and mechanisms of regulation. This may facilitate the identification of other potential points for therapeutic intervention.

## Data Availability

The crystal structure for MAP4K4 was accessed from the protein database (PDB: 4ZK5) and assessed for key structural regions using CCP4mg [22]. The novel sequence identified for rat MAP4K4 (accession no. KM217582) is available at https://www.ncbi.nlm.nih.gov/nuccore/KM217582?report=GenBank. Other sequence information including the 5′-RACE product are provided. The proteomics data as supplied by the Fingerprints Proteomics Facility (College of Life Sciences, University of Dundee) are in Supplemental Spreadsheets S2–3 (Band S1), S4–5 (Band S2) and S6–7 (Band S3). The computational models of the coiled-coil domains were generated using IntFOLD and MultiFOLD. The IntFOLD server produces data on the atomic 3D coordinates for protein tertiary structures, as well as providing both global and local model quality assessment scores. The MultiFOLD method produces atomic 3D coordinates for quaternary structures of proteins and evaluates the quality of the modelled complexes. The IntFOLD output files conform to the CASP data format standards for machine readable prediction files. All of the atomic coordinate data files conform to the Protein Data Bank (PDB) format. The use of these standards allow the data to be easily visualised and interpreted by other researchers. All of the data files are open, non-proprietary and freely available via the links below. **Tertiary structure predictions from IntFOLD** MAP4K4_351-494: https://www.reading.ac.uk/bioinf/servlets/nFOLD/IntFOLD6results.jsp?time=16_2_11_790_7-10-2020_All&md5=ppale3tmdia19gdp&targetname=MAP4K4_351-494 Human striatin, striatin 3 (SG2NA) and striatin 4 (zinedin) coiled coil fragments: https://www.reading.ac.uk/bioinf/servlets/nFOLD/IntFOLD6results.jsp?time=16_4_7_444_7-10-2020_All&md5=d18ueit2vvbluio5&targetname=striatin https://www.reading.ac.uk/bioinf/servlets/nFOLD/IntFOLD6results.jsp?time=16_4_56_323_7-10-2020_All&md5=pprblrcpmhjrbrbl&targetname=striatin-3 https://www.reading.ac.uk/bioinf/servlets/nFOLD/IntFOLD6results.jsp?time=16_5_30_665_7-10-2020_All&md5=295b5b0r3v0mlj1k&targetname=striatin-4 **Quaternary structure predictions from MultiFOLD** Striatin-MAP4K4 complex: https://drive.google.com/file/d/1skHPFYgR-nrqArJBpDPosm0cY3o4RRnA/view?usp=sharing Striatin3-MAP4K4 complex: https://drive.google.com/file/d/1IJ_9-3s-qbGB2GEthwIVZX65kZTux2U5/view?usp=sharing Striatin4-MAP4K4 complex: https://drive.google.com/file/d/1z33bBwu7dEb5PNp9CUSAiSera881hUPP/view?usp=sharing All other primary data are available from the corresponding author upon reasonable request.

## Open Access

Open access for this article was enabled by the participation of University of Reading in an all-inclusive *Read & Publish* pilot with Portland Press and the Biochemical Society under a transformative agreement with JISC.

## Abbreviations

CalA: calyculin A
CNH: C-terminal citron homology
DMEM: Dulbecco's modified Eagle's medium
FCS: foetal calf serum
GCK: germinal center kinase
HEK293: human embryonic kidney 293
JNK: c-Jun N-terminal kinase
MCS: multiple cloning site
NIK: Nck-interacting kinase
PP2A: protein phosphatase 2A
STRIPAK: striatin-interacting phosphatase and kinase
WT: wild-type

## Supplementary Material

Supplementary Figures S1-S4 and Tables S1-S4

Supplementary Spreadsheet 1

Supplementary Spreadsheet 2

Supplementary Spreadsheet 3

Supplementary Spreadsheet 4

Supplementary Spreadsheet 5

Supplementary Spreadsheet 6

Supplementary Spreadsheet 7
