# Supplementary material for: MAP4K4 expression in cardiomyocytes: multiple isoforms, multiple phosphorylations and interactions with striatins
Source: Biochem J. 2021 Jun 11;478(11):2121–43. doi: 10.1042/BCJ20210003 (PMC8203206; doi:10.1042/BCJ20210003)
Supplement: Supplementary Figures S1-S4 and Tables S1-S4 [file BCJ-478-2121-s1.pdf]

Supplemental Figures and Tables

Supplemental Figure S1. Alignment of N-terminal kinase domain of the predicted protein sequence (September 2020) for rat MAP4K4 with mouse and human sequences. The kinase domain is in bold type.

Human MAP4K4 (NP_001371419.1)
Mouse MAP4K4 (NP_001239129.1)
Rat MAP4K4 (NP_001100374.1)

MANDSPAKSLVDIDLSSLRDPAGI**FELVEVVGNGTYGQVYKGRHVKTGQLAAIKVMDVTEDEEEEIKLEI**
MANDSPAKSLVDIDLSSLRDPAGI**FELVEVVGNGTYGQVYKGRHVKTGQLAAIKVMDVTEDEEEEIKLEI**
---MDVTEDEEEEIKLEI

NMLKKYSHHRNIATYYGAFIKKSPPGHDDQLWLVMEFCGAGSITDLVKNTKGNTLKEDWIAYISREILRG
NMLKKYSHHRNIATYYGAFIKKSPPGHDDQLWLVMEFCGAGSITDLVKNTKGNTLKEDWIAYISREILRG
NMLKKYSHHRNIATYYGAFIKKSPPGHDDQLWLVMEFCGAGSITDLVKNTKGNTLKEDWIAYISREILRG

LAHLHIHHVIHRDIKGQNVLLTENAEVKLVDFGVSAQLDRTVGRRNTFIGTPYWMAPEVIACDENPDATY
LAHLHIHHVIHRDIKGQNVLLTENAEVKLVDFGVSAQLDRTVGRRNTFIGTPYWMAPEVIACDENPDATY
LAHLHIHHVIHRDIKGQNVLLTENAEVKLVDFGVSAQLDRTVGRRNTFIGTPYWMAPEVIACDENPDATY

DYRSDLWSCGITAIEMAEGAPPLCDMHPMRALFLIPRNPPPRLKSKKWSKKFFSFIEGCLVKNYMQRPST
DYRSDLWSCGITAIEMAEGAPPLCDMHPMRALFLIPRNPPPRLKSKKWSKKFFSFIEGCLVKNYMQRPST
DYRSDLWSCGITAIEMAEGAPPLCDMHPMRALFLIPRNPPPRLKSKKWSKKFFSFIEGCLVKNYMQRPST

EQLLKHPFIRDQPNERQVRIQLKDHIDRTRKK-------
EQLLKHPFIRDQPNERQVRIQLKDHIDRTRKK-------
EQLLKHPFIRDQPNERQVRIQLKDHIDRTRKK-------

Supplemental Figure S2. Exon structure and sequence of the rat MAP4K4 gene. The full-length sequence for the longest potential MAP4K4 isoform was determined by a combination of bioinformatics alignment of the rat gene with mouse and human genes, 5'-RACE and PCR cloning/sequencing from neonatal rat cardiomyocyte cDNA. This is aligned against the rat genomic sequence (NC_005108.4:46657454-46782545 Rattus norvegicus strain mixed chromosome 9, Rnor_6.0). Protein-coding exons are highlighted in cyan (exons identified in rat cardiomyocyte cDNA) and yellow (bioinformatics), with 5' and 3' UTRs in green. Initiation and termination codons are in red. The predicted polyadenylation signal is in bold italic type. The position of the 5' RACE primer used for cloning and sequencing is in bold type underlined. Exons M1-M9 subject to alternative splicing identified in Wright et al. [16] are indicated.

CCCGGGGGGTGGGTGGGGGCCGAGGGAGGGCCGCCGCGCCATGGTGTGAGCCCCGTGGCCCGCGCTCGCCGCGCTCCGCTCAGACCAGACGACA
CGCCGCGCCGGCGGCGGCCTGCTTGTGCGGCCCGAGCCCCGGCGCCGCCGAGCCCCTGCCCGTCGCTCGCTCGCTCGCCTGCTCTTGGGGCGGC **Exon 1**
GACCCGGGGAAGATGGCGAACGACTCTCCGGCTAAAAGCTT**GGTGGACATCGACCTGTCGTCCCTGCGG**GTGAGTGCGGCCGCGCCCTGCGCGC
CGCGGTGGACACGGACACCCGCGGGCGCGGCGGCCCCTTTGTCTGCTGGGCGAGCGGGCGGGCGGCTTCCGCGGCGCTCCCGGGGACCCGGCGG
GGCGAGCCTTTGTCACCGCCCTGCATCCCTCCCTCCGCCCGCTCGTCCTCCTCGGTCGGGGTCCCCCTTTTGAGATGATAACCTCCCCCTCCTC
TCTCCCCAGGACCCTGCTGGGATTTTTGAGCTGGTGGAAGTGGTTGGAAATGGCACCTATGGACAAGTCTATAAGGTCGGTGTGGGGCGCCTTT **Exon 2**
GTTCCCCGTGGCGTGGAGAAACTTCTTAGGGGACGAGGGAAAGGGTGACGGAACACTGCTGACTGCAGGCCGTGCTGCCTGGCCAAAGTTGAGA
AAGGGGTTTTCGCCTGGCTTTTCTTGTTTCTTGTTTCTTTTTGTTTTTCGCCTGTGTTCAGACTGGCCTTAAAAGACCTCATTTCCTGGCTTTG
AGATAAAGGAAATAAGCTACACTGTTAGGCATGCACTTAGCTTCAGCAAAATAGAAAAGGAATTTTGTGTTCGAGGGCGACCGAAGGGTCCCCC
CCCCCATCCCCCTTTCCTCCAAATCTGGCCTCCATGCGTTTGCATTTGTATGTGAATTAAGATTCAGTGTAGACAGGCCTTGCCTATGATTACA
CTCAACCACTGCCTTGGGATTCTTGAGAATTGTCATCATGCCTTGGGAGTGCTTGCCAAAAGGATTTAACTTGGGGAGATTGGCTTTACCTGGA
ACTGCTCTTACCCTCTTCTGGTGGACAGTATTTTGGGGAAGCCGAGAGGAGTGTGCTTTTGGGCCACAGGAGCAGAAGGAATTACAGTGTGGGT
ATTATAAAATGTGAATTCCAGGAATAACCTGTGTGACAGCTGTGTAATTGAGATGGCTAGGAGGAGAGAGAGCTAAGGTGTAGACAGGATGCCT
GCTTCACACCCACCACCCCTTCTCTTTCTTTAAATGTACCCTCCTCTCCAGGCTGCAAAGGAAACTAAGGGAGCTGTGAGTTCGTGCTGAGTAG
CTGGCCCCTGATGTTGCTAACTGTTGTTGTTCATTCCGTTTTGCTCGTTCTAGAACTAGAAGTGGGGCTCAGGCTCCTAAGTGTTTAAAAACAC
GAACAAAGCAAAAAAAAAAAACACAAAACAAACAAAGCAAAAAACCCTTTTAAGTTCTCTTTCCTGGTTTCTTTCTTGTTAGCAAATGGTTTTC
TGGAATTATCTTTGCTTGGGGAGTTTTCTAGTTACTGGGACTGGGTCAAGGTTGAGCTACAAAGGAAAAAAAAATATCTTGTGAAGGAGGGCTT
TGTGACTCGCCATGAAGAATTGTCATTGTTTCCTTAAAAATGGGGGTGGTGGTTGAAGCTGACTGTAGACATTTTCTGAGGCCAAATTAAGTGT
GGATTCTATTGTCCCTTTTCTGTTTTATGAATAGGGTTTATGTGCTTCTGATTGCTTCTCCCAGTTCCAGCTTCTCCCTTCCCCCACCACAGGG
CCTGGATGGTGGTGTGGACCGGCCTGTGGTCTGAGCCTGAGTCAGTGCTCTTAGGTGATCTCAAGTGAATTAGCATGTTTTGTTGGTTAACTGT
TGGGGCCTGGGCCAAATTTTTGAATTTGAAAGGGAGCCCCGTTAACTGCCTGCTGGGTCCAACTGAATTTGGAAGGCTTTGGAAAATTTTGGAA
AAAGACTGTTGCAACTCACTGAGCAGCTGGCTGGTAACTGGCTTAAAGACACTCTTCCCAGCTCCAGAGTTTGTTTAAGCACCGGTTGGTTTTT
TTCTAACCTGTTTTTTCTGGCCAGATCTAGTTGTGAGTAGACTCTTCCAGTGAGTATAGCCAGCGCTTGTTTTTGACCTGATTACACAGTACTT
GGATTGTTTGTGTCTTCCCTGGGCAGCCTGGAAACTGTGGGTAGAGAAGGAAAGCCAGAAAGCCCTAGTGCCTTTTAAAAGGGCCAGTGCGGGT
TTTAGTTGGTTTTAAGAGGGGGAACACTTTGTGGTGTCAATCGGGCGGGTTCTTTGTCCTTCCCACCTCGCCCCCTTTTGAAGTAGATTTGCTA
AATAATTGCTTTTGGTGTGTGTGGGGGGGGTACCAGGGATGTGCTAACTACACCAGTGCAGTGAGAATCGTCTGTGCAGATCTACTGAGGACAC
TGATGTAAAAGTTAAGTGAAAACCTACGAAGTAGTGCTTGGTTTTACTCACAGTAGTCCACACCCCCCCCCCCCCCCCCCATATTAAGGACATTC
TCAAGCATGCAAACAGAGTTTTTATAGCTTAGTTTAATAGCCCACCATTGGTTTTTGGTGTGTATATCCTTTCTTTCCTAAGTCTTGAACTTAA
GGTAACATTTCATTGATAAGTACCCCAGGTTTGTTTTTCTTCTTTGTAAACATTGTCACGATGTTATTCCACACATCAGACTTCCCCCAGTGTG
CTGTGCCTGAATTGGTAGCAGATTAGTAGGGGCTACTACATTTATGAAGCGGCACAGGAAGAACTAGAAATGGCATCTGTTTCCCTTTTCTGTT
GTGCAGGTTGGTTTAGTTTCCATTTTTTTTTTTTTTTTTTCCGGAGCTGGGGACCGAACCCAGGGCCTTGCGCTTGCTAGGCAAGTGCTCTACCA
CTGAGCTAAATCCCCAACCCCTAGTTTCCATTTTTATTGGTCGACATGGGGTTAGGTTATTAATAGTAGAGTTTATTAACAGCTATAAAGACAA
ATTTTAGGAAAAGGGTTTTCAGGTAAACCATTAATTGAACAACCTAGAGTCCTAAATTATTTCAGCATGCACGCGTGCGCGCGCACATAGGCAT
GTATATACACATACATATACACATGTATGTGGTATGAGTATCCCCGTGTATGTGTTTGTACGTGGGTGTGCACATGTATGTGGAGGCCTAGGTT
TGATGTCTGGTGTCTTCCCTTGATTGCTTTCTACCTTGTGAACTGTGGTAGGGTCTCTCATTGAAAACTAGGGCCTGTCAGTTTAGCTGGTCTAG
TTCCAGGCCCCCTATGTCTACCTCCCACATGCTGGGATTACTGAGGGTCCCCATATCCACCTGGGTCATGGAAATCTGAGTTCCCATGCTCACG
CTTGCTCTGCAAGTGCCCTGCCTCCCGAGCCGCCCTCCCTGTCCCTCTGCTCGGTAGTGGTATCTAGTGGGGCAGGACTGGGAGCGGACCTTAT
TTTGAGGGGTGTATTTAATACCGTGAGGAGACTGGAAGTGAAAGCGAAGTCTCTATGAAGATGACTTTTTGAGGCTACATTCTGGTTTTTGAAA
TATTTTTAACACGAGTAGGCTAGTGTGGTTGAATCCTGTCTGCCGTCTTTTCTTTCAAAGAAGAGTTAAGGTGCTAGGGAGAAGGCCCAGCAGC
CAAGAGTGCATGCTGTGCTGGCATGAGGACTTGAGTTCAAATCCCCAGCACCCACATAAAAAGCTGGACCAGTAATGTAGCACTGTGAGGTTGG
AGTCAGGAGGACCCCTGGGACTTGTCGTCCACTCTAGCTCCAGATTCAATCCAGGGAATAGGGCAGGGGTGGGGTGGGGTGGGGGCCATGGGGT
GGGAGTGTTGACAGAGGAACATGTCCTTTGTTGGCTGGGTGGTAAATATAGGCATGTGTATACACCCTTCCCCCCCCACACCTGCACATATGGT
TACATGCAGGTAATATACCCCCCAAAGGTGAAATTAAAAAAAAAAAAATTGAAAGACAATGTATTTGTTTAGAGAAAAGTATTCCTAGCCTTAGG
TATTTGAAGGATCACGTGGAAAGATACTCATAGATTCAAGCTTAGAAAAAATAATTTTGGGGGGGAGTCCTTATGTTCAGTGAGCAGGTGCTTG
CCTGCTCTGAGCTGGACCTTCAGTTCTGTGTCTGGAAACCTGCTGTGCCTGGAGTTGTGTTTATAAGGTTATATTCAGAACACTGTATGCACAC
GAGAAATGTCTGTAGCGGTGGCCGGTCTGCTTACTGCTTGTCCGGGTTGAATCAATGGTGAATTTTAATTATCATCTCTGAGGACAATTATAGT
GTGGAAGACTGAGGCGGGTAAAAAGCTTACTGCCGTGTTACTTTGGGTTTAACTTTTCTTTTGAATATTTGAGAATTTATGTTTCTTTTTCCCT
TGGGTAACTACCAGTATTTTATATTTTGCGAGCGTATTGCCATCCCAAGAAGGAGAATTCTGGTTTGAAGAGATTGTGGTGTGTCAGAGAAGAC
TTTAGTGATAGTCATTGCTGGGGTGAGGCGGGGGCCTGTGGGTCTGAAATCTGGGTGCATTTAGGGAGGCACGGAGGACAGGCCAGATGTGCTC
TGTTTTTCTGTCCTTGGAGGAGGGGGCTCGTGTGAACTGGGAGGTTAAGCAGGAGGCAGGTTTAGGCCCAAAGGCCTTGCTTGACAGGGCAGTT
AACATCGAGTTACTTAAGAAAGGCAGAGAGGGATGAGGGTGTGTGGAGGATGAAGGACCAAGAGCGGCCATCGGGAATGGCCAGAATGGTAGTG
TCAGGAAGGCAGTCTGGGCGGGAGCTGAGGAGTGACATCATTGACTTTCTGTAAGCACTGAGGATCTGTTAATAGGTATGATTGGGCTTGGGGA
TCTCTAGGAGTAGGGAACCTCATCAGATGATACTCTGGGAAGAACAGTTAAGACGGTCGGCCCAGGACAGAGGTTCTGAACCTAGGGGCTGCAG
AATGACCCTTTCACAGGAGTCTCTCACTGAAGACCATGGGAAAAACACAGATATTTACATTGCGATTCATAGCAGTAGCAGAACTACAGTTATG
AAGTAGCAACAAAGGTAATTTTATGGTTGAGGGTCACCCTTAAAGGGATGCAGCATTGGGAAGGTTGAGGATCACTGACCTAGGACATAAGTCT
AGAGGGGAGTGGTGCACGTCTGGGCAAGGGGCTGCCGGATGGAGACGAAGAGGAAGCAAGGTTGAGGCAGATAATCGGGGGTTACTCTATGTCT
CTTAACTTGGGGGATGCGTGTACAATTAATTGTAGGCGTTGGTTCTCCCTTTTAAGTGGCGTTTTTTCCACTAGATAGGGTGGAACTTCTGTGG
ACAGGATAGGAGTGTTACACGGTTGGTTCTGATCGCTGCTCAGGGGTAAAGTGCCTCAGCTGTTGTCTGGCAAGCAGAGTATCTCTTGTTCCTG
GGGGAAGTGAAGCCATCCAGCGGGCCCTGACAATTCTGAGCGAGATGGTCCGTCCTGAGGAGTGTCAGCTGACCTGCAGTTCAGAGGCATGTTC
AGACTTGTTCTGAGACTTGGGTGTCCTTAATGCTGGAAGGATCCGGACGTGGGTAAGGTGGGAGGAAGCCAGCCTGTTGAACGAGAAGTACTGC
TGTGTAGCTCTGCAGACTATCAGTCCCAAACACTTACCTGCTGCTTCCGGAAAGCAGGGACAGTAAAGCCAGACAGAAATCCACCCTTCTCTGG
GGCCTGCTGCTGGGGTTTTAACTCTCTGAAACATTGTGACAAGTGTGTACTCATTTGAACTGCGGCAGACTTGTATTTTGCCTCTTTGCTTGGC
CAGCATTGGAGATGTGTGTGTTTCTGTTATTATCACAATCCAAGGAAGTTATCCGTGCCTTTCTGATAGCACTTGTGTGTGTTCACTGCTCAGT
CTGGATAAACAAAGGAACATTAAAAAGGAATTGAGCTCCTTTTGGAGTAATAATATGCCACAGGAAAGATGTGCTGGGCTAAAGAATGGTCATC
CAAGGAGCTTCACTCCCAGGGAGTGAAGGTCCCGTGACTAAGAACCTGAAAGGGAGATGCTTTAACATCTGAGTCTGTGTGTGCTGTTTTGTTC
ATCTCTTTGCTAGTGTTGGTAAGTACTTGCCCACTGGTATGAGCAGCTTTGTGAGCCAAGCACTGTCCACACTTAGTGTCAGACAGTTCTCCTT
TTCACCCTCTTGTAAGAAGTTCACAGTGCCCTGTGTGATCATTGCTTGCATTTTCCCAGTTATTCATTTCAGTTATTTAAAAAAAAAAATAGCAG

GTTATGTAAATAGTTGCAGAAATTAACACTCTTTGGACATAGAAGGTCAAGGTGCTGAGACAACTCTGGTGAGAGGTGACATGTGACCTCAGTG
AAAGAGATTTTATGGACAGCAAGTGCACAAAGGCATATTAGAGTCAAGGACTTAGTCTGCAGAGACTTCCCCGCGTCCCATATTTATTAGAGTG
CACAGTGGTTTTAGGGGTTTGGATTAGTGTTTACAGTGTTTCTCTGGCAGAGTAGATAGATACCTAGTAGCCTATGTTATACAAAGAATCTTCA
GGTAGTTGCTTTGAAATTTTAAATAGAGTTTTCACTGTTTGGTTTCTCATCTGGATGGTCAGGGTTCCCAATTACTCATTTAACTGTAGAAGAT
GATTAGTTTGCTTTGCTATAGGATAGGTAGCAGGAGGGTTGAATCAGGGTAAACACTTTTGGGAACGATGGCTAGGAAGAAGGGCAAGGGATAG
TATATTATGAGCAGCCATAAATATGTTTTTGTGTTTAATAATTTTCCACCAGTTTGCCAATAGAGTGTGATCTTCTGCTGTGAGACCGTGTGAG
GGCTACAGACTGCTGGTGTTGTTTGGGTTAAAGCAACGGCATTTTCGGACTTTTCATGTAAGACCGAGACATTAGGATGTTTTATATATGGGAC
CACTAAGAGGTATTTAGAACTAAATTGGTGAGTCAACAGGAGCAGGTACAGCCTTGGGTGTTTTCGGGTACCCCCTGTACTATTTCCTGCATAG
AGATTCTGTGTTAGTGCATTGGGTATGTGTGGGGAGTATTGCTTTCTTCAATGAATACAGGATAGAGGGACACACATACACACACAAACCACAC
CACAGTGTGCGAGAGCACACACACACACACACACACACACACAGAGACATGCGCCCCCCCCCCCCACTCATGCACACACACATGCACGCACTGA
CAGATATCAGGGTATATTCTGTAATCATTCCCAGGTCCTCAGCCCATGTCTCTGCACCCAAACACATGGACCCAGCACAGCTCCCTGCTTGTTT
TTATGAGACAGTTCCTCACTATGTAGCCTTGTCTGGTTTGGAGCTCCCTGTGTAGACTATGATGGCCTTGAGCTTTTCGATCCACCTGCCTCTG
CTTTCAGTGCTAGTATTAAAGGCCTGTGCCACTACCACCAGCCAACTGTTGCTTTTAAAATGAGGCAGGGTCTCCTCAGTTTATCTCTGTAGCT
CAAAAAACTCAGTCTGGTCTTGAACTCAAGGTTCTCGTGCCTCCATCTCCAGAACGGCTGGGACTGTAGTAATTAATGCCTTTTATCTGGCTGC
AGTGTTCCTTTTTAAAGTTTCGGTGGCTCAGAAACACTTAGGTAAAAGCAGGTGACCAAAAATTGAAATGGAAGGTGGATGTCCCATAGAGTGC
CAGTGTGTAGTTTGCTGTGTCAGTTTGCCGTAGGTGTGCTGTGCTGTTTCTGAGACGGTCTTCTTTGCACCTGCGCTATGAACTGTGCTTTAAG
AAGCAAGGCCCTGGCTAGCAGAACTGATTTGGAGCAGGCTCTCAGCTCACCTTACTTGGCACGTGAGCAGGCTGTTGTCAGTGTCTAGCAGTCC
ACTTCCTGCCATTGAGAAACAGAGCCAAGAAACCTGTGCTTCCAGATTTGTGTGTACACATTCTATATGTGGGTTACATACCTCTCTATCACAG
AAGTCTCTTGTGTTTTAGGGTAGGCATTGTGTCTCAGATGTGGCTATGTGTGGACACGTTATCCTATTACGTTCAAGTAACTAACAGAAGAACC
TTACCTTTGGTTTATAACTCAGAACACCCCTGTGTTGTTTGTTAATAGAGAGTTTAGAACGTAGTAAGGTCACTGGTGAGTGTCGCTGGGGTTA
GCAATCCTTAGATGCTTATTTCAGAACTTGGAGGTACCCTGCCCTTTGGAGCATCAGTTTTCAGCACTGTCTGCTCTGTGATTTTTTTTTTTTA
AGCTTATTTTTAATGATGGTTCCTAAATGCTTTAACTTTTCTTTTAGCCCACCTCCTCCTACCAGAGGTAGTGGGAAAGGAAGGATACAGGGGA
AGTGGACCTGTTTAGAAAGGTTTAGAAAGCAACTCCCGTCTGCGTTGTCTGGAAAGCAGCAGTTCAGTTCACAGGTCAGCAGCCACTGCTCGAT
CTATTCGTAAACACTTCAAGGATACACCAGCAGTCTGGTTCAGCAGAGTCAGGATAACACGAATTAGCAGTGGTGGCATGACCTAGCAGAGACA
GCTAGGCCTCAGCCTTCACACGAGCCATCAGGAGGGACCAGGGCCAAGAAGAGCTCAATGTTCTCGACTGTGCCTCTCTCTCTCAGGGAAGCGA
AGATCTCAAGAGCCGCACAGCTGGCTCTATTAGCATGTCTAGCTCACGCCCCTTCACTGTCCTTCCAGTTCTTTTTATTTGTCCTCCAAACATC
ACGTGTCCTCCATGTGCCTTGTCCTAGCACATGTGTCTCTCTCAGCTGACATCACTCTGCCAATCAGCCCAAGTCCTCGGAAGTGGCAAGAAAC
TGCAGCACACCCCTGATAGTTTTTTGGTTTGTTTCTTTCTGTGGAGTCCTGACAGATGCGGCTTAACTATGCAATGTAAGGCGGACCAGTACAT
GCATGTCGTTAGCAAAGAATCTTTCATCACATGTCCTTTCACAGGCTCGCTTTAGCAGGACATCCTCCCTCCACTCCCTTCTCTCTCCCTCCTG
TCTGCTTCAGCTAAATGTTCCTTCACGAGTCTGCCTTAGTCTTTCACCTGTGTCCACTTTAACAGAACACCATCCCACATAACCGACTCTCCAA
AGAACCCTTAAGTACTTCAGTCTGCTGGAGCGCTGTATATTTACTGTCCTGTCTCATCTCCCTAGTGCATGCTGAGGCCCGCCTTTCTCAGTTG
ATTGGCACTGACTACATCCTGTGATAGCTGCTACTGCTGCTGGGCTACGGTAGTTCTAGAGCAGTTGACTGTTTGTCTCCTATTCTTGCTCCTC
CCACAAATGAGGTCATCAACCGGGGGCTAAAATGATAGTTATTTATGTTTTCATGACTGTGTGAATAATCTTTATCTGAGCCAACTGGAATGTG
ATATTTTCTTGTTAAAACAACACCCTCTTAAACTGCTTAGCTCTTTTAGGCATGGTTTTCAGTGTCACATGATTGCTTTCCCCCTCACGCACCC
TCAAAGACAAGACAAGCACCTAGCAGCGTTCAGCCTCTCACCCATTTTCCCTAATGCTGAATGTGTATTATTAATTGTTCTGGATAGCCTGTTG
ACTTCACTTTGGAGCGTTTCACTTTTGAGAGCTGCGCAGAGCAGACTGCCCCTAGACCCAGTGTCCAGCTGTGTCAGGCGGCATCCCTGTTTCT
CCTGGTCTTTGGCTTGCTGTTTCATTGGATGAAATGCATCCATCCTCCAGTGGCTTTCGGGGAAAAATGTACAGGGTGTGAAGTCGCTGCATGG
CCTTAAGCTCATCTGTTCCGTCAGCGTCTGGAGCAGCCCTTCCTCTAGGGACAGATTTCTGGTTTGCCGTGAGATGGTCGCCCAGTTGTGTGAG
GCAAGGAAGGGGCTGCAAACTTAGATACATTCATTCTCTTGATTCTACTTCTGTGCTTACTTGGTATCCGTTGCTTTCAAGCAATGACGCTTTC
TGGCCTCTAGAGGGCAGGCTGCCTGGCTTGCTTTCGTGCCTGGCTGGCGCCAGTTAACTGCTCTTCTGTCCCCTTTCAGTTTTTAAAAATTGGT
GTTTAAGACAATGAGGAGTTTGGAAGCTAGGGGGTCCTTGGAGCTTTTTCCTATTTCCTGTAAAGTTTACCCTGTTGAAGCTTATATTGCAGTG
ATTTTTTTAAAATGCATTTATAGGCCAGGGTAGCCTTTAGTTACCCACAGCAGACTTGGGCACTGTGGTTATCTGGGTAAGATGTGGATACTCA
CTTATAATCACTCCCTATTCCAGCTCACAGCACCAGGCAAGTACTAATGGTCCTGTTTGTGCGAGTTTGCCTTTTTATTTTTTATTTGAATTAA
CTCAGTGCGCAGCACCTGATTGATGACCTTCACTTCCCGTTTCCGAGTTCGTCCATGTGGCGGCCTGGCGGTTGGTTTCTTGTTAGTTCATAGT
AGTGCAGCATACGGATGGGCCCCACCTTTTCCTAGATGTCTACCGGTTGGTAGACACTGAAATTGTCTCTCCCGTTGGCCCTTTTGAGTGATGC
ACTCAGGAGGTGTGTTTTCCTGAAGAACACCGGGCGGAATTGCCTGTAGGAGAGAAGAGGAAGCAGGAGTTTGACTGCGTGGGGCTTTCCATTT
CTGCCAGTCTAAGCAAACTCCTCGAGTGTGCCTGATTTCCGACTTACAGCTCAGCTGTGGATTGGGGAGAGCACTTGAAAAGTGCACTTGCCTG
AATCTTTACCTTCCTCACTCTTGCTCTAAGGTTAAGTCGTATACTGACCCACCAAGACCAACTGTTTACTGCCACTGAATCAGCTAACACCAGA
AACCAGGCTTTTTCCCTGTAGATTTTGTGCCGTGGCACTGAACACTAGCATCTCCCTTTATCCCAAGTAGGAGCTGTGGACCTGACCAGGACCC
AGTCCAGTGACTAGAGGGGCAGGCCTGAGAGGGAAGTCCTGCGTTCCACCTGGAGTTCACTCAGGGCAGCTGGAGAGGACTGACTCCTGTCGCC
CGCCGGGGGCATCTGTGTTGCACGCTTAAACATCTGTGATACTTGGGCAGAAGAAACAAGTGTATTTCAAGTTAAATAAGTGATTTTCTCTGTC
ATGTGTGAGGGGGATAATTTTACCCATCTGAATTTGCTTCAAGGGTTTCTTTAGAATGTTTTATGGTAGGGGTAGTATTATGAGAAACTTGACT
AACTACACACACACACACACACACACACACACACACACAATGTATATATAATGTATATGTATATATGTATATATAATTTTATCAGACTTGAGGTAT
TCAGCTCAATTCTTACTAACTGTAATAATTGTGCACATTTTGAAAACTCTTGACTTGGGAGTGTTCCTATCCATGCCAGTCAGACTTCTTTACC
CTCCTCAGTTTTTCTGATGGTCCCTGTGTGATCCCTGTGTAGCTTTTGTTATTGCGCTGTTGGGATCTCGATTGTTATTTGAGTAAAGACAGCG
AGGACAGATCACTGCATAGGGATTCTGAGCCTCTGTAATACTGGGTGTGGTGAGGGCAAGCCTTCCAGTGTCAATGGTGTTGGGCGTGTGTAGC
CCCTCCAGCCTCTGAGTCTGTGGGTGCTCATTCTTTCCGTTCCTGCTTCTCTCTCCTTTGGTTTTTCCAAGAAAAAAAAAAATTGTGGCTGCTCA
GCCCTTTCCAGTGATTTAAGGGCTTAATATTTATTTATTTATTTATTTTTTGGTCCACTGGCTGCAAATATTTTGAGCCTCGTTGGCTGGGTGA
GATCTCTTCTTTATTACTGAAATACTTGGTATGTGTATAATGCGTGCCAGGCACCGTGTGAACAACGGACAAGAAAGCCGACTAGCCTGTGCCT
CCGTGTAGTTTCTAACGCTGGGCGGGAAGTGAACAGGGCAAGAGAGTGATGTTACAGATAGAGCCCTACACGGGCTGTGAGAGCCGTGAGACTG
GAGTAGAACTGGATTGCCAGGTAGAAACAGTGATGTCCACAGTGGAGATTCTTTGCAGGAGGCACAGCTATTCGAGGAGAAACAGATAGTACTT
AGATTTAAAAGAAGCCATTAAGCCTGCTGTGACTTATTGTCTTGATTAATACCGGAGAGAGATTTGAATAAAGAGAAAGGGCGAGCGAGAAAGG
GCAGGGAGGGTTCTACATTTACCAAGGTGACTGTCTCTTTCATTTTTCCCTGAGCTAGCCGAGTCTTGGGAGGCCTTGCTGCCAACATCTGCTA
CTCTTAGACATTCATTGTTTTGAATTTCCTGTACCAGTCTATCCATGCAGCAACCTGGACAGACATCTCTTCAGAGGTAGGGTCTCATTAGGCT
GGAGTGTTGTTTGAGGAGTATTTAGGATAGTACTCGGAGAATGAAGATGAAGCAGCTCCCGTGTTCCCTGTGTACGGAGAGGAGGCTACTGTCT
TGGGCACCAGTCCCTATGACCTTAGCGCTGGAAAGGGGTACCCCTGTAAGACGGTTACTTTGGTTACAGATTTTTGGGGTAAACTCATGCACAG
GTTTGAACGTTTCTATAGTGCTGCTTCTAGAAGTAAAAAGATGATGGGGGTGCATTATTACTGACTGCCCTTTGTGGTGTTTTACCTTCCAGCT
GTGTAGCATCAGTGTATGTCAGTGAATACAGGGGTCACTACTCAGGTTGCATCAATCGTGGGGCCTGTTACTCAGCACGTAATGGTTCGCTTTT
CAATAAAGTGGACAAACAGTCTTATTTTTCTGAGCTAGCTACGTAAAATGGCTTTGTTGGGTTTTGGAAAGAAAAAAATATGAATTGAGCATTC
CTATTTTAGTTTTGAGAATTACATATTTGATGCATGTATGTACATATGGATATTGTCATGTATGTACATATGGATATTGGCATGCTGATGTCTC
CAGGCAGCAGGGAAAGAGAAGTAGCTGAGTTCTACATCTGGATATACAGGCAGAAGGAGGAGAGACACTGGGCCTAGCCTGGGCTTCTGAAACC
TGAAAGGCTGCCCTCCCTGACATGCTCTCTCAAGCAAGGCCACGCCTACCAACAAGGTCACACCTTCTAATCCTTCTAAGTAGTTCCACTCACT
CCCTGGCGACTTCAGCAATCAAATATATGAACCTGTGGGTGCCATTCTTACTCAACTGCCACATTCCACTCACAGGGCTCCATAGGCCATATCA
CAATGCAAAAATGCATCCAGTCCAACTTCAAAAGTCCTCAGGTCTGTTACAGTCATCAAAGAACCATTTAAATGCCCATTAAAGTTCAAAGTCT
CTTTTGAGACTCAAGACAATCTCTTAACTGTAACCCCCTGTAAAATCAAAATCAAAAAGCAGATCATATATTTCCAACATACAGTGGCACAGAC
TATACATCTGCAGCCCCAAAGGGAGGAAGGGAGCATCGTGAGGAAACACTCGACCAAAACAAGACTGAAGACCAGCAAGGCAGACGCCAAACTC
TGCATCTCCACGTCTGATGTCAGAGCTCTTCAGATCTCCAACTCCTTTCAGCGTTGTTGACTGCAGCACACGGCTATCTCTTGGGTTGTTTCTG
TTCCTGGTTCTCAGTTGTTCTTGGCAGGGATCCCACATCTCTGCATCACCAACGTCTCTGCATCACCAACGTCTCGGGGTCTTTAATGAAGTCA
GGCTTCCTTCACAGTCACAGGTTCACACAGTAGCCTCTGGGCCGCCGGCGTACAGGATCGCCCCTTATATATACGTGCTTCTTGTATCTGTGGG
TTTGTACTGGTTTCAGACAACTGGGTTTGTCCTGTTTCCCTCCCTAGTTCCTAGGCTTGGGATTCCTGTTGTGGATATTAGCCTTAGATCTGCC

CCGAAGCAGCTCGCTCACCCACCCACTCCATGCTGTTTCCTCAGACTCTCTTCAGGGCTTCATTTGGGGTGGTGACCGTAGCTCAGGCTTCATG
GCCACTCCTCCCCCCTTCACCCTTAGGACTTGTTTATTAAACCCTGTGTATGTATGTATTGTATGTATGTATTTGTGGATATATGCATTGAGTT
TGGTGCCTTCCGAGACCACCGAGCGCTGGGAACCAAACTCAGATCCTCTGCAAGGGCCATATGTGTGCACTTCTGAGCCACTGAGGCCTCTCTC
TGGCGCCTGCCTTGTCTTCTTGTCCATGGGTCTGTTTGCTAGTAGTCCCATCCACTTTTGTTTTTTTTAAATCTCAGACATTAGGGCGCGCGCG
CGCACACACACACACACACACACACACACACACGCACACGCGCGCGCGTACACATTTAGATCTTTTTGGTATTTTACTGCTTTTTCAGAGGTCCTG
AGTTCAAATCCCAGCAACCCCATGGCGGCTCACAACCATCTGTAATGAGATCAGATGCCCTCTTCTGGTGTGTCTGAAGTCAGCAACAGTGTAC
TTATATATAATAAATAAATCTTAAATAAAAAAAATAGCCGTATTGTTTGCTTTAACAATGTCAATGTCATCTCTAACCTGTCAGTCTTGCAGTC
TTCAGTGGACTTGTTGTTCTCTGTGGGTTATGACTCCCAGAATGTTAATCTTTGAGTGGATGCCACTTGCCATGAATTTTACTGGGTTTTAGTG
CTGTGGTTACTACTACTACTACTACTATATTATTATTATTATTATTATTATTATTATTATTATTATTATTATTATTATTATTATTCCTGTATTG
AGGCTCATCTGAGAGACAGTTCTGTTATGTAGGGGAAGGTCAGTTTATCTTTGAGGACCCATTCCTCTGGTTTTTAAGGAGGACTTAGCAGTGG
TGAGTCTCTGGCACTAGCTTAGTTTCCTGAGTTCTGCAGTCAGCCGTCTCTATGCGAGTACTGGCTCTGTCCCTTCAGCTCTTCTCCGTGCATG
TCTCTTCTCTGCCCTAGGTAGTTTCCTTATATACGTGTGTTGATTTGTACTTGGCTGAACTCAGCCAGGCAGGCCCCCCTTTTAGACCTTGGGG
TTCGTTATGTCTGGGCAGCCTCTGGTATTCCAGTTTTACCTCTGGTTTCTCAGTCTAACCCAGACTCTCCGCTGCTCCTCACTGAGGGAGAGAG
CAGGCTGGTGGTCCTTGCTGTGGTTTCCTCTTCTTGAGTCTAGTTTTGAAGGGCGTCAGTAGATGTAGCAGTCAGAGATCAGGGTCACTCTGCG
TTTCACTGTGTTTCCCCTTCTGCCCCTTTTCCTTCCTTCCAGTTGAAGACAGTTTGCTCTGTGTGTGTGGTTTATGTGCACATGCAGATGTGTC
GGGCATGTTCTCAGCATGTCAGAGCTCCTGCACAATGCTGGTGTGGAGGAGAGTAGAGCATCCAGTGTCTTCCTCTCTAACTCTCTTCCTGTTC
TTTTGAGGCATAGTCTCCACTGAATCAAGCTCCAGAGCTGAGGTGGCGTTGATTCCATGGGCGCTGTGATCTGAACTTAGGTCTGTGTGATTGC
ATGCCAGTGCTCTTAACCGAGCCGTTTCTTCAGCCCTTCTCCTGAAGTCAGATCTTGCTGCGTAACTAATTAGCTGCCTTGAGATCTTCCTCTT
TCAGCCTCCTGAGTGTTGGGGTGTGAGGTGTGTACCGCCACATCTGGTCTAATGTCCAGGTCTCACTGTTACCTGGCTAGTCTGAAAATTGATA
TGTACTGGGATTAAATGTGTTCACCAACACCTAGCTCCTCCCCTTTTTTGATGGTAGTACTGGTGTGTAGAATGAACAACGTTAGAAAGAGCTT
TGTTGCCATTTTTTGTGTTTGTTTGTTGTTTTGAATCAGTGTTTCTCTGTGTAGCCCTGTCTGTCTTGTAACTCACTTGGTAGACCTGGCTGGG
TTTTTACTGCATTTAGAGTTACACAGCCCTTCAGTGTGATGACAACTTCACATAGACCCATGTCCTAGTCCTTCTAGTGTCTGACAGGCTGCAC
CTCAGCCCTCTGTCTTTTGTAGCTGTAGGTACTGTAGGCATTTCATGTAAGTGGCCGCACACAGTGACTGTCTTTGTTCAGCCTGATGTCTTCA
AGGTTCATGCATGTTGTGGTGGGTGTCAGTACTCCGTGTCCCCCTTGGTCTAGTCATGTTCTATGACTACACACAGTGCACTGGTCCATCAGTT
GATGGTGCACACAGGCTTTCCATTCTGAGCTCCTAGGGGTGAGGCTGCTGTGGGCATGTGTGTGCAGTTCCTGTGTAGCCCTTCCGGACTGTAG
GAATCTATGCTAACTGACTTTTTGAGGAACTATGGAGCGTGTAGTTTCTGTGACGTTCCCACCAGCAGTTTAGGAATGTAGGTATATTTTTTTA
CATGCTTGCCAATGTGTGTCATATCTGTCATTCTTCAGAATGGGCATGGATGTTTATCTCATTTGTGGTTTTAATTTGCATTTCTCAGATGACT
AATGATGCCGAGCGGGTTTCTACTTGCTTTTGGCCTTTTGAGTATTTTTACGGGAATGTTTTTCAAGTCTTTCAGCTGTTGCTTAAGTTGGGTG
ATATTATGATTTATGAATTTTCTGCCTAAGTGTTCTTTGTATGTCTGGGCTGCTGGGTATATAATGTGCAGCATCTTTCTGTGGTCTCTGATTG
CGTTTTCACTTTCTTAATGGTATATATATTTTTTCAAGTACATTTTCCATTTTGATGAAATTGGAAAGATGCTTTTTTTTTTTCTTGCTTAGGA
TGCCGGTATCCTATCTAAGAACCTTTCCCTAATCCAAGGTTACAAAGGTTTATTCCTGTATTTTCCTCTTATTTTTGTTGTTTTAGTTCTTAGA
TTCACTCCTAGCTAACTCGTGCATCTTTTAAGTTGGGTGTGTAGTCTCCCATTTACACATGACTGTCTCCTGGAGCACCGTTTCTGGAGAGAAC
TTAAAGGCCCCACTCAGTTATTTGACACACGTTAAGCTTGGTGACTTGACTGAGGGCTGCTGGGCTCCCGAGTGTCACTACAGTGACTGCTGT
GTCCGTCTGTCTCAGGCCGATATCACGGCATCCTGATTTCTATACCCGTCTGAAATTAGGAAGCAGGAGCCTTCTTCCAGAACAGTTCATCCAA
GAGCCTGTCCAATCTGCCCATTGTTTTACTGGCAGCGGACCTTGACACATGGCACAAACTTTTAAGGAACGTTACTTAGCAGTTGCTACGAACT
TTTCTATTCAGTAATTATTCCTCTCAAAGTTCTCCTTTTTTTGCAGACTAATGTGCTTTTCTACATCACACATTCGTTACATATCATGTATATA
ATAGACCACCATGTTTTTTTAAGGGGGGCATCTAGAAACGTTGCTAAGTCCTACTTGCCCTACATATACTCCAAAGAATAATTTGATAATGCCA
GTGATTTCGGGATCTTAACGATTATAAACTATGGGCCATACAGTACAAAAGAGGGCAAGATCTTTCGATTAATGGTAATATGCCAGTATCTAGA
TGTAAAATATCTTTAAAAATTATGCTGTAAAATATTTCCAGAATGTACAAATTCTGTATGTAGGTGCATACTTCTTAATATCACAAAAGTGTCC
TGTGATTTAGGGTAGGCTGGGGACTGTCCATGGACCTCTAAGTAATAGTTCGCTTTCATTTTCAAGAACCAGTTGGTCCAGAATTTCTGTTAAA
CCCAATTGTTAGGTTCGCTTAAAATATTTCCTGTATTAGGAGGACCTAATGTTTTGCTGTTTCAGTGTCCCAGTCTGGTTGTAACAAATGGGAC
AGTTCCTAATCCTCCAACCTCTGACCACCAAGAACATTCCAGGGTCTGTCCTTCTCATCGGCCACCCCAGTGCCTCATGGGAGAGTAACTGTTC
AATGCTCAGTGCTTCTCTCATTCAATATAGATCGACCTCCGCTCTGATCCAGAGCTCCAGAGAGCAGCGCTGGAGACCTGGATCCAACCGAGTA
GCCCTGGCCGTCCTGAAACTCACGGTATAGACCAGGTTAGCCTCAGACGCCCAGAGATAAAAGGTGTGGGTCACCATACCTGACTCTTTTCACC
TTTAAAGCATAGTTTCCACTCACTGTGTTCAACCGCTGGTCTATAAATCTTAGTAAAAGAGGAACTATCCAGAACACAGATTTCATCAAACAAC
CCATTGACTACACTACAGAGAAGGAAGCTTTTGCCAAAATGCATTTCCTTCACTTGTGAGGAAATGTTGTTGAGACATAAAACTTCAACTCTG
AGAAAATGGTGGCATTTCTGTGCAGCCTATAAGGAACATACTTTTCCAACATTTCAGTAGGGGTCTCTGAAGAAAGTATTTTCTCTCCATTACA
TAATAAGCACAAGAAACTTGTTGGACATTTATCCCAAGTGACCCAGAAGCAGTGAGGGTGAAGGTCAGCTCTGATACTGACTGCGCAGGACGAA
AAGTTAAAGCTCCTTTCTGTGCTGTTGTGCGGGCCATTCACGTTCACTTAAGGCTTAGGGACAGTCTGGCAGGACATTTTGCTTAAAGCCTGGT
CAAAGAATGGATCTTCTAGAGACTAGACCTTTTTCTGGATACAAAACCCACCGTCCTGGGACTGAAATTTACTTCCTGCTAGTTAAATGAAAAT
CAATTAAAAAGTGCTTGTCCCCTCAATTTCCATGAATTGCAAATTTATTTATTTATTTATTTATTTATTTATTTATTTATTTATTTATTTATTT
TGGAGTTGGGGACCGAACCCAGGGCCTTGCGCTTGCTAGGCAAGCGCTCTACCACTGAGCTAAATCCCCAACCCGGCAAATTTATTTTTTAATA
ATCCTTCCCAGCCCTACTCCTTTGGAACAGTGTCTTGTTATGTTGTCCTGTCCGGCCCTAGCTAGCTTTGAATTTTAGCAGTCTCCCACATGTT
GGGATTCTCTGAATGTGACATCATGCTTAGCCTGGTTCTCGGTTTTCACCAGAGAATTCGATAACCGTGTGAGACCTCGAGAACTAGAGGAACA
GAGGTGTGTGTGCGCGGTCTTTTGCTTGTGCCACCATAACTGATGCCACTGCCTAGGAGGCTGGACAGCAGCACTGGCTTCCTGGGTCCTAGAG
ACAGAAGCAAGAGGTTGTGGGAGGGACATTTCTGGTCTTCTGAGGCCCTCTCTTCTTCCTGCCCTGTGGAGGCTCTCTTTCTCTTGTGTTTCCC
ATGACTTTTCCTCTCATGCCTCCCTGGTGTTTCTCTTTGTGGTCAAATTTCCTACTCCTAAAGGATATTGGTCAGATTGGATTGGGACTCTTAA
GTTTGACTAATACGTTCCAAGTTATTGTAGGTTAAGACTTCCACTTAGGAAATTTTGAGAGAGACTCAGCTGGCCAACAACAACAATAACAACA
ACAACAACATCAACAACACACACATACATACAACAACAGCACACTAACAACAACAACACACATACATACAACAACACATCCACTCCCTATTTTT
AAGACAAAACCACTTGAGTACTAATTGAGTACTAACTGCTTTATTTACCGATCAATTTGGAGCCTTATTTCTTGAAATACAGTGGGCACTGCGA
TCAGGTAGGTAGCACGTTCATTTACTGTGGGAATGCCAGATGCTTATGTAGATGGTTGCAGATACAGAACTGGTGACAGAAAAATGCAGCTCTG
TGCTTTCTATGGTTTAAGACAATGGGGAGGAAGTAAATGTGCCTTTCTGGGGTGAGAGCCGCTCTCCAAGCTTACTGGCGAACCACATCTTGTT
CTCTCATCATCAGGCGTTGGGTTGGGTCCACGCTCGGCACAGTGGCTCTCCTCCTCTCTGCTCCAGCCCCCTCGGGCAGCTGCTGCATCACCCG
AAATCACTAGAAATGTTGTGGTGTAAGATAGTGTTTGACAGACGTTTAGAACACGAGTGTTTTTTTGATCACATTAGTTGCCTCTTCCCTTTCT
TACCTTTAAAAGTAATGTTAAAAAAGATACTGGGTGTTGTGGAAGAGTGGGTGTTGGGGGATTGAGGTGGGGATGTAGTACTGACCTTTAAAAA
CACCGTCATTCTAGCGAAGGGTCTAGTCACCATTAATTACAGTATTAGCAGATGAGGGTGAGGGGTTAGCATTTTCATCCATCACTTTGATAGG
ACAATAGCAGAGTCACCCAATGGCTTGCAGTCTTTGCTTTCATCTAGGAGCCTCAACTCATGCATGGCAACTTATGCTAGTTAGACTTGCTTTT
CTTAGCTCAACAGGAAATGAGAACAGGGCTCAGTAGAAGGAAGTCATATACACCCAGCTTTCAGGGTGCAGGCTCCTCATTGGTTGTCTAGGAG
AACAGTCAGGTTGGGCTCACTCTATATAATGCTGACTTTGGAAAGGAACTCAGGTACAGTTACAGTAGACTCTGAGTCAGTAGGTCTAGGTAAG
GCCTGGGACTCCTCTGTCAGAGTCAGCTCTTCTGTGATGTCAAAGTAATTCCACGCCTTGAATACTGAGGATACAGGACGCTGAAGTCTGCCCA
GGGTGTTGGGGGTTAGGCGTCTGCTGAGGGCTTTCACTGGAACATACCTAATCCAGTGCCAGATTTGGATAGGGATTGTGTCTCATCTGTTCAG
GGTGAAGTGGCTTGTGTAGCCACACAAACTGATGATGTGAATAGATTGTATGTAGCTTTTAGTGAGGATTCTAGGTTTTATGGTGCTTGGTAGA
GAACAGAATATTAGAGATGGCAATGGCTAAGCTTTGGGTTCAGAGGTCAGGGCGGGGGCTTTGTGGCTGCTCAGGTATCAGACTCCCCGAGGTC
GGAAGTCTCCCTCCCGGAAGTACAGAGACGCGTGCTGCTGCTTCCTCGCCCCCACACTCGGCATTTTTGGACAGCATTAGAAACTCGGGATGGG
CCTCAGATTCCATTTTGGTTGCTAATATGCTTCTAGCAGAGAACAGTGTTTGAGAGAGACCTGCCTTCCAAGTGAGGGCGTCACAGAGTTGGCC
CAGGGCAGAGGGTTTCTAGAAGAGACAGAAGAGGCCATTGTGGTGCTGTGGAAGAGCTGCCGAGCCGTCCAGTGACAGGCAGACTACAGCTGTG
TTCTGAGTCAGCAAGGGTGGGCAGGTAGAAACAGTTAACTGGGTAACTGAGGGAGACTCTTACGCACACCCTTCTTCACTTTGATTGTGCGCCC
TTTCTTGCATCTAGGAAAGCAGTCAGGAAAAGGTGCTGTTGTTAGAGGAAAAGCGTCAGACCAGGTCATGGGAATACAAGTGCCTTAGGAAGTG
GGTGGGAAGTAGGAAGAGTCAGATGAGTGGAAGGAGGCTGAGGAAGAAGCAGATTAGCCATTTTCTCGGCAAAGGTCTGCATGCCATCTAAGCG
TTTCTGTGGGGGATGGTGGGTAAGAATCTGTTTCCTTTACCTCTTAGAAATTCCTGTGAGGCAGAGGGCTCACATTCCACCCCTCCAAATGAAC

TATCTTTGTCTTCCCACTGATAGCATTATCTGAAGTAAATTATTCTCTGGAGTTTCCAAACTAGTGGCATGAAGTAAAGAAAGAGAAAAACTAC
TAAGCTATTTGAAGATACATTTCCTTTCTAAACATTCTGAAAGTTGCTGCAAGAATTCTTCAAGCCTGGCTATCCATTGACAAAGTGGCTCCAG
GACTAGGGTTTGCTGGGTTTCCCCTTGTTCATCTTTAAGGATGTGGCTCTGTCCTGCATGCCCTATGTAGCTGCAACCTCTGTTTAAAAAAATT
TTTTTTTAATTATTTTAGCTGTAGGCACTTTAATAAATGTTATTTTTTTATCAGTTTTATTTTTTTAAGACTTTGATTTCTTTTTTTTTTTTT
CTTTTCTTTTTTTCGGAGCTGGGGACCGAACCCAGGGCCTTGCCCTTGCTAGGCAAGTGCTCTACCACTGAGCTAAATCCCCAACCCCGATTTC
TTTTGTTTTTAAAGATTTGTTTATTTTATATATTGTCTGCATGTGTGGATATATATACCATTTATGTGCCTGGTTTCCCGGGTCCTGCTCCTAG
CACCTCCAGGTGTGACTCACAACCATCTGTAACTCCGATTGGAAGAGACTCCAAGGGTCCCTTCCAGTGAGCAAACTGCTGACCAAGCCAGTAA
GCAGCACCCCTCCATGGCCTCTGCATCAGCTCCTGCCTCCATGTTCCTGCCTTGTGTGAGTTCCTGTCTTGATTTCCTTCAATAATGAACAGTG
CTGGGGAAGTGCAGGCCAAATAAACCCTTTCCTCCCCAGCTTGCCTTTGGTCATGGAGTTTCATTGTAGCAATAGAAACCTCAGGTAAGAGACA
GCCTCTTCCTCCAGGACCGAGTAGGACGCGCGCTCTACTGTAAACAAGCACATATACAGAGTAAGAAAGTAAGTAGTACTCCATACCTTTCCAA
GCGGAGGACAGGACGATGCGCTCACATTCAACACAAGGAAGCAGTGTTTCCACAGTGGACCAAGTCAGTTTTCCACGCAGTTGAGTTACTATAA
ATGTCCGTAATCGGTTAGTGTTAGTGTCAGGTCAACACCTAGGAGGCAAGCCCTTCATGCCTCCGGGTCAGCCTCTGAGACACCTTAAGAGATG
GACTTGATTGTGTTAATTCCTGTGGGAAGTCCCATCTTCACTGTGGTTCGGACCATTTCTAGGGGCCCATGCTGGCCTATATACAACACAGGGG
CAAGCTGAGCACTATTCAGCTTACTGCTCTTCCTTTTGAGTGGAGATGTGCTGTGAGCAGCTCTCTCAGGCTCTTGCCACCATGGTGACTTCCT
TGCCAGGATGGACCACAACCTGGAATTGTGAGCCAAATAAACCCTTTCTCCCTCGGTTGCTTTTGAGATTTTACCACAACAGGAAGAGAAACTG
AGAGCCATCATTGACGACACCCCTCCCCCAAAGAAAAGTATGGAAGAAAAACTTGGCAAAGGATTTCCAACTTGGATGTCCTGTCAAGTGTCCC
ACCGACACTGGCTGCTTGGGTGCACTACATTGCAAGTGCTCAAGGAACAGCCAAGAATGCTCACCCTGTGGCTTTACCTGTGGCTGCAGCAATG
CTCACCCTGTGGCTTTACCTGTGGCTGCAGCACAGGCATTGATTTTATTCTGATCTTCCTGTGACTTCCTTGTGCGAGCAAGACGGACTTGCTG
CTCTCGGATACCGGGCAAGTGTTCGATTAGCTGTTACCACACGGCTATGACTGTACCACTGATCAGAGCTGCCGTTTGGCTTTGGGATAAAGCG
TCACAGCTCCCCAAGGTGATTTTGAGCACCCTGAGCAGTTCCGTCCGGATAGTCCTGCTTTTCCCCTGTAACCTATGGTAAACAGTCTCCAAAC
TCCGAAGGCCATGGATGTAGAAGATGCCCTGGGCAGGCGTAGCCGGAGTCAGGGACTACCTCCCTCCAGCTAACCTGAGCAGCGTTACTGGCTG
GGAGCAGGTCTGTGAGGTGGCTGAGTTGTTGCTGGGTTTGGGTTGTTTTTTGACTGAATACTGCTGGGAAAGTGGAGCTCTATGACAAGTCTCC
AGCTCAGTGTGAGTCACACTTCCTCTCATAGAGCTGTCAAAAGCTAGGGCTGTGCATCCTGCTGAGCTCTGCCAGGCTCCTCTAGTGTCTTCCC
TTGCCAGTCCTCCCGGCCATCCTCCTCAAGGCTTCCCTTGCCAGTGCTCCCCGCCATCCTCCTCAAGGCTGGCAGTCTCCCGGGAAGGGAGTCT
TACACAGGCCAGGACAGGAGAAGTAAATGTCCCTTTCTAAAGGTGGATTTTGGATTCTCAGGATGCTTGAGAGGATTACCTCTGTGTATAAACG
TGTCTTGCTGACAGTTCCCAATAGCCATCAAACTTTCTTTTAACTCTGAGCCTACTAACTCGATGAAGGGAAATCTTACCGAAGTCACATTTGT
GAAACCGGAATGTTAAATCTACACTCACAGATAACTAGACAGTCTAAATTACAAAAAGTGTTATGAATAATGCTAAGTGCAGTTTGGTGAGACT
ACGGTGCTTTGAAAGGTCAAAATGTGAAATCTGGTTTTCATTCCTATGAATCATAGGAAGTAGTCTTAAAGCTGTTACTCTCTGAAGACTGCCC
GCCTGATGCAACAGATGCAAAATCCGTATCACCTCTGCCCAGATTCAGCAAGAAATGCTTCCGCTTGTTTTTCCGAGTTATATAATGGCTATGA
GCAGATAGACTGGGTAATTTTTCTCCTATCAAGAAAGCATTGACAAAAGATTTCTTTTCCTGGGCAGGGACATTGAGACAGGGTCTCAGAAGCC
TAGTCTATTTGATATTGAATCCTTCTGTCTCAGAATTACAACCTAGTCTAACATGCTCAACTCAAAAGGTTTCTTCATTAAATTGCATGAGTAA
ATTTTCTGAAATCACAGCCCAGGGAACGAAAGAAAGATGCTCGGTAGTTAAGTGTTGCGGTGAGTTGAGCTATGCTGCTTTGTAATCCCAGCAC
ATCCTGAGTTTCCAGTCACCTGACCATCACAGACCCTTCATCTGTGAATGTAAGACAGACAGACAGACACACACACACATACACAAACATACAC
ACACAAACACATACACAAACACACACACAAACATACACAAATATACACACATACACATACACACACAAAATCTTAGTTTTGGCTAGATCAAAGC
ACACACATAATCTTATTTTTGGCTAGCTCAAAGGCGGGGCAGTTCTGCCACTCTTATAGATTTCCCTCCAGTACTCCTGACTCAACACTAAATC
TATGATCTGTCTTTGCTGCCCTGTTCTTCTGGGTGACCCCCTGTCTTTGCTGCCCAAGATGCTTCTGCTCTACAGCTTGATCTTATGGAGGTGT
TTTCTCAATGAAGGGTTCCCTCCTCTCAGATGACTTTATCTTGGGTCAGTTGATGTAAAACTAGCCAACTTGGGCTATCAACATTAGAACTGGA
AGGGTTTCTCTGTTTTATTTTTTCTTTTTCCTGTTTTGAGGGTTGAACAAGCTCTGCAGGCACCCTGTTTCTTACCAATACCCCAGCCAACGGG
ACTGTGTTTTTTATTCTTGCTCCGTGTGTTGTATATGGGCACATCATTAAAATGATGTTGTATACACATCGTTTACAAACTTTAACATTATAGT
AGGAGCATATACATATTTGAGGAGGTTCCTTCTCTCTCTCTCTCTCTCTCTCTCTCTCTCTCTCTCTCCCGCACATACACACACTCGTAATTTG
AGCAACTCATCTAAGTTAATGTTCTACTCATGGTTTCTTAAACATCGTAGTAACACAGGGCAGAGTGGCTCTGTGTTCTGGTTGTTTTTCAGGG
TAAAGGCAAGCAGAAGACTTCGGGTCAACAGGCAGCAGATTCATGAGTTGGCTATAATTTGTTCTCTAAGCAGTCCTTGCAGAACTATTAAATC
ACTGTTGGCATTAAGTTGTTTCCTTTCTTTTTTCAAAGATTTACTTATTTTATGTATACGAGTACACTGTCGCTGTCTTCAGACTGAAGAGGGC
GTGAGGTTTAAGAAAGCTCATCCCTGTTGCTTACCTGCCTTGAGGGGCAGCAGCTCAGTTCTTGGATTCAGGAAGCTGCAGACTTTTACCTTAA
GACATTTAGAACTGCTCTTCTGAAAGTTCTTTCAGAGCACCAAATACTTAACTTTTTGTTGCAAGTGTCTGTTTTCATTCTTGACATTAAAAGA
AACCCTAAACTCCCGTGACCGAGTTAAGCAAGCCTTGACCCTGGGAATTAGAGAATGGTTACTCCAGTCACCTGCTCAGGTGTAAAAAGCTTCC
TCAGCACTCCTAGTTAGTCCTTTACCCATGGTCCTTGCAAACCTGAGCTCACAGGCTGAGTGACTGTGATGTCTCTGATGCTTTCTCCTGGAAC
TTCTGCCCTCATGGTGTTAACTAGAACTCTGAGCATCTCCGCTCCTGTCTCTGTGTGTTCCAGTTTGATAACAACCTATGTAAAACCCAGGGTA
CTTGTGGGTGGACACAGTGTCCAGGCTTTCTGCTCAGGCTGTGTCCATACAGTGAAGTCTCTGAAATTGGAGCAGCCTGGGCTGATAGGAGCGG
CAGAGCGTCAGAAACCAGGACAGACCCCTCCCCGGATTCCTGACAAATTAATTATCTTGAGATTATGCCAAATCCTGTCTTGTCTTTGGGCCTC
TATTTCCTCCTCTACAGTAGAAGGTGTGGGCCTCCTCATCTCTCAAGTTTGGAGTGTTTTCAGAAGATGGTTGTTTTGACATCTAGTATGGAGT
CTCACTAGGAGGCAATCAAGGTCTGTAAGGTATTTGGCTCACTTCAGACAAACTAGAATGGGTGCTGAGATGCCTCTCCAGCCCTGCCCACTTC
AAGTATTTGCTTGCTTTAATCTTATGGAGTATAAGTGTTTTGCTCGCAGGTAAGATCACCACCTGCATGCAGTGCACACCAAGGTCAGAAGACA
AATCCTTTGAAACTGGAGCTATAGGTGGCTGTGAGCTGCCACGTGGGTGCTAAGAGACAAACTCTGGCCCTCCATTAGGACAGCCAGTGCTCTT
AACTGCTGAGCCATCTCTCCATCCCCCCTCCCCCTTTCTGTGCCTTCTTCCGGCAGTCGTCGTGACCATCCTTATTGTCATTCTTACAGCTACT
GCTATAACTGTGTGTATGCTTGCATGTGGAGGTCGGAGGATGTCAGGATCTGCTACGGACTCTAGAGCTTACCAAGTAGGGTAGACTGGCTGAC
AGGCCCCATGTCTGCTCTCCAGTGCTGGGACTGCAGCCACACCCCCACTGCCCCTCACTTCCTTTTCTTTTAAAAGCAAAAGGGATGGGTGCTG
GGTTCGAAACTCAGATCTTCACGCTTGATCTAGGGAATTCGGAAGAATTTACTGTGATGACTCTTTTGGATCAGAGCGGGCCTAAAAAGTAAAT
GTGCTGGTTCATTCAGACTTACAATCCCAAGAGGCTGAGGCAGGGGAATTAAGAGCTCAAGATCAGCCTGGGCCACATAGTAGCTTCCAGTGTA
GACTAGAAATATGTGATTGTTCTAGACTATATCTGGTTTATTGTGGCTTGGCAGGTACATATGGACGCTGTGCCATAAAGAATTGCCAGTAGCC
AGATAACTTAGGAGCTTTTCTCTGTTATTATATTTAGATTTTATTCTTATTTTATTTTTCATTTTATTTTATGTGTCTTGGTATTTGTATTTGC
ATGTGCGTCTGTGCAGTGTTTGAGAAGCCCTGATGAAGGTACTGGATCCTTTGTGGTTGTGTTATAGACGGTTGTGAGAGAAGGTGGAGAGGGG
GAGAGGGGGAGAGAGGGAGAGGAGGAGCCAGTGAGCAGTGAATCAAACCAGGATCCCCTGGAAGGGCTGACAATGCTTTTCATTGGGCTCTCTC
TCCAGCCCCTGGATCTTATTCTCAACTAAAAACCCACAGCCAGTGTTAACAGATCATGGAGATCACTGCAGGCCAGGAGGAGGTGATCTTAGGC
TTTTCTGGGGTGCAGGAAGAGTTGACTGAAGGCCTTTCTGGGGTGCAGGAGGTCATCTCTTTTCTTTTGCATTCCTGTAGCTTCCCAGGTGTGG
CTTTCTAGACTATCAGTTAGGTCTCAAGGTTAACTAAAAACAAACTATCTTTATTTGGGAGTTAAAAAAAACCCCACAATGGAATGGTTAGATA
GTGTGTCTCTTTCAGTATTTATGTAAGAACAACATGGTTCTGTGAGTGATTTGGCTGGATTGTTTTATGTGCGATAGAAAAATTTCATTTGACC
CTTGTGACTTTCTATTTGCAGGAAACATACTTAATTTGTTCACGAAGCAAACATCTTCCAGAAAACATCTTCATTTGATACTGATGTATCTTTT
TTTTTTTTTTTGGTTCTTTTTTTTTTTTTTTTTTTTTTTTCGGAGCTGGGGACCGAACCCAGGGCCTTGCGCTTCCTAGGCAAGCGCTCTAACA
CTGAGCTAAATCCCCAACCCGATACTGATGTATCTTAAAGGTCCCAAGATAACAGACTTTCCCTAGGGTATGCTGTGTCATAGAATCCAAGGAA
GATGTAGGACCTTGCACGAGCTTCTTCCTCCAGGATACATTGGAAACAAAATTGTTTCTGTTAGATACAGCTTAACAGATACAGCCCATGAAAC
TGTTTAAGACCCTAAACTGGTTTTTTCCAAGTACACATATAGAAGTGTATGTAAGGAAAGAAAAGTGGGTGGGGCACATTTGTTTCTCTATTTG
ACACTTAGCACTTTTGTTAAAATTTGTATAATCTGGTTGGCAGTATAGCTGTGTGGTAGAATGGGTTTGATTCCCAGCACTGCACATATATGTA
TGCACTCAGGCATACACCTGCACACACAAGAATAGAACTGTATCTGGAGCACAGTTAGAACTTCAGGTAGGAAGTATCTAGTGAACGAGTGAGC
TTCGAAGGTTGCCATGGTGCTGTTCTTCATAAATCAGTGTGTGAGGAGCCTGAGTAGGTGACATTAAATTCAGACCAAGTACCCTGCTGGAATT
AGATCCAGAGCTGCTCTCTGTGGAGAAGCCTCTCAGACCTTTCTTCAATGGCCCTTTCCCCTGAAGGTTTCTCGGACACAGCAGATTGCTGGAC
CCAGCTCTACAGTTTCCCATCCAGTAGACGTGAGATAGTCAAGCCTCTGCCTCTGTAAGCAGTGTGAGCACTGCTGTTGGCCATTGCTCGCCTG
GGAGCTGTGCTTGGAGGTTATGGAGGCTTCACTTCCCTGGGGCTTTCTTCTCAACATTTCATTGTGTGAGCGCCCAGGCTGTCACATTTTATAA
TGTTATTTTGCTTTCTTTCCTAGTGACCTATTTACCCATTAGTCTAGCATTTTGTTACTTCAAAATAAGTTACAGAATTTAGTATTTTTTGCTC

TGAAATGTTTCTGCTTAAGTAGCATTAAGTAGAATGCAATGTTTTTTGTTTGGGGCGGGGGGGGGTGTTTATGGGTGATGTCTTACCGTGTAGA
CCAGGCTGTCCTCGAACTCACAGAGGTCTACCTGCTCTTGCCTTCTGAGCACTGGGATTAAGGGCTTGAGTTATCATGCCTGCTAAATGGACAG
ATTATTAATGAGTTTGATAATATACGCATAATCTAAATTCCTTTCAAACTGCAGAACATCACCTGGGAATTAAAAAAAAACCTTAATCTGATTT
TATATCCCTCTTGATTCATCTGGGACAAGACCCACACTATGCTAAAGATATGAATGTGCAGCCAAATTAGGAGCCAGTACTGCAGCTGGCTTTG
CATCTTATAGTATCTGAAGGAAAATGATTTATACTCAAGAACGTGGCAACATTAAGAAACATATCGCGACCATAAGAGAAACTGGTGACTGTCT
CATGTTCATCTTTGACCAATACCAAGGTGCTAATTTTCTACATTGTGAATTGTGCAGAATGCAATTTAATATGAGGATCAGGTGGTTACTCTAA
AGATTGCCCTCAGCCTTCTATGCGAGCCAGAATTTTAATGCATTTATTCTCTAATTGAGCACCTCTCTATCCAGCCATGCCCATAGTTTTCAGA
CAGTGCTTGGTCCCTCCCAGTTACCCAGGCTTGCTTTGGAGAACTCATGCTTCTAGGTGCTACTCTGCAATGAGGCTCGTGTTCCCCACACCCC
TGACACTTAGGGCTTGTGAGCTGTAGCCTTCTGATGGTGCTTTGTAGGGAGAGCATTGAGGCAGCATGAAGACTAGGCCTGTGCCCTGAATGTA
GTGTCTGGGCAGCAGCGTGATCCTCAAATTGGGTTCGTCTGGATCTCTAGCCTGGGAAGAGAAAGCTCCTGAGAAAATGAGCTCAGATTTTCAC
AAGAGAGGATATTAGAGAAATAGAATTTTAGAGTTCAGCTTGACAGACCCAGGGGCTGGGAGTCAAAAGGTCTTACGGATAGGAGCCACAAGAG
GAAATAAACATGGAGGCACACTGAGGCACAAGTGCTAGAGACAAGACAGCCAGTGGGCCAGGTGTGGCTGGCACAGCAAACTTGTTAGTTTTGA
CACAGATTCCGAGTGGTTTTGAATGACTGCCCCCAGGACAGTTAATTGTTTACCTGCAACTCCACCTACTTTTCATGGCACTTACTCATGACAA
TGTGGCAGACTTGCATGCCAGTGCTGTTGCTGTCCTCAGGGTCCTTCCATTTGTGAATCCCACACTGCCCTCCCTCCCCACCCCGCCCCTGCCT
CTAACCTCATACATAACACATTTTCATGAAAGAGTTCAATTCTGTCTGTGCAGTTTCCCGATGTCACAGGCTGGGGCACCCGTTTTGGTTGACA
CTGGTGTGGAGTAAACCACCCAGCCTGCATCCCCCCATTGTTGCTGTCTCTCTAGCCTCTGTGGGGCCTGGACTCTTACCTCATTTTCACCTCT
CACTTAACACCTCTGCTGGCCTCTGCCTGATTCCCTCACTGTGCCCGGCTTATAAATGTAGGTTTGTCTGTCACCATAGCATTCCACAGTCTTT
GCTTAGCATTTGGGCCCCCACACCTCCTGGCTTATCACTGTTGCTGAGTTCAGCCCAGCCGGGCAGGTGTAGCTCATTCTCAGCCACCCTAAGA
AGTTCTGTTTGGCCTGTTCCCCTCCCCCGCTTCTCCTATCCACGTGACTAACCCCTTAGCGCTGTGCTTGCTCTGTAATGCCTGTTCTCCTTTC
ACAGCGCTCTGCACGTTTTAATGTCAGTGGTGGTCCCTGGAGATGTAGAATTCGTGCTCCTGACACTGAGTAGGTTTTGGCCCCCACAGGGCGC
TACATCCACCTTTGTCAGAAAGGTGTGTATAAGAGTTAGAGCCTGGGCAGCATTTTGACTTGACGGCTCCTCCTTGCTCGCCCTTCTCAGGTCC
TCCTTTCCCCGTTGGGGGAAAGTGTTCATCCTCTCCACTGTGTTATCCTGAGATGTCCACACACAGGAAAGGAAGGAGGTTGGGCAATTTGGGG
TGCTGGTCATGGAAGTTAGTCATTCTGACGAGTCCGTGAAACACAGGTGATAAAGCTGCTAATCCCCGAGAGTCTTTGTCATCATGAACAGCTG
GCATTCCGATAACTGTGGGGGTGGGGCTCTCGCGCTCTTGCCTTTTTGTATAGTAGTAGATGGTGCCTTATTTTGCTAAAGAGCTTGCAGTTTT
CTTCTTGACTCTGAGATGAAATCACATGGGCCACATGGTTCCATGAATATCTACAGTATCCACATTGCTTCAAGTGACTGACCAGTGAGGTCTA
AGGGCTAAGGCGAGGTGTTTTTCTTTCTGTCCCCTCCGCCTTTGCTGTTACTAACTGATGTCTCATTTGATGATCATGGTAAGATTGGGAAATA
CTAAGTAGTTACATTTGGATAAGAGACAGCTCTGAAGTAATCGGTGGCGGGCCTGCCCAGCGCCCCATTGAGCATGTCGATGAATGCACACAGG
AGATCCTGCGCTCTCTTTCCCAGCTGTCCCTCCTTTGAATCACGCAAGACCAGGGTCTGTGTTTGGGTGACTCAGATGAAAGAAAGTTTGGAAA
GTAGAAACTTGTCATCTCTGCCTGCTTGTTAGCATTCAGGGCCTAAACAATGGCTTTCCCTCCTGCTCTCTCTCTTCCCTCACTTAAATTCTTG
TTGCCTTTATGTCTCCCTACGCAGACTTAGTTTTCCAGGAATGTGTAGAAATATAGAAGAACGCCACAAATTAATATCTGCCAACCCTGCTGTG
TGGCAGGCTTTGGCTATAAGTACTTGAGGCATGGAGAGACTTGGTAATATGCACTCTTCATGTGCACCTCCTCAGGCCTGTGGTGGAGACTGTC
AGCAACAGTTGAGACTGTGTCCTCTCCAAGGGGAAGAGGAAGCCACTTCTTTCTGGAGTCTCTAAGCACCTTTCATTGAGAAGGGTGGGACGTG
CAAGGCTGAGGTGTCACAGAAACGCAAACCTGCAAGTACTAACGGTTTCCGTGGGAAACCAACTTCAGCTTGAGCACCGTCCCATCCTGTGCGC
CTTTATGTCTTGATAAAAGAATGCTTCTAGTGTGCACCACAAGAAACCAACACAGATTTTAAAAAGCACATGCCTCTTTTTAGTTTGGGGACGC
TAAAATCAGTTTGAAATCAGTCAGAACAATGGAAGATAATTTGGTGTTTTGAGAACTGTATGGAGAGAAAAGTAAGAATGTTCTACAGAAGGAC
TTTTGGTTCATTGACACAAAGTGATAAAATGGTAATAATAATAATAATAATAATAATAATGATTATAATAATACATTCTCACCATTTCTGT
GCTCGGCAGTGCTCTGACCCAGGCTCTGCTTCTGTGCACATAAGATGATTGCTCTTCTTGCCCCTTGCTCACACGTGCACTCTGACTGACACAC
ACACACACACACACACACACACTCTCTCTCCTTCCCTTCCTCCCTCCCACCAAAGTAGTAGTGACCTTGTGAAAGAGACTGAATCGGAGCACAG
GCTGAGTGGCATACGAGGACCGGTTAGAGGACCATAAAGTGATGGTGTGTGCTCCCCGAGATGACGCTCATGCCATGACCCATGTGATGGAGTA
GCCAATAGGGTTTACGGTTTCATCTCTCCTTGGTCCCTCCTGAGGCTGCGTGCTTGTGTCCGGTTAACCGTGGTTCCTGTGAGGTAGATTATGT
GACTTAAGCTCATCCAAGGCATGGCACCTAGGTGGATCTGCTGATACTCCACGGCGAGCCCTCGAGCCCACGAGGGCCCTCGGGCTGAGTTCCT
CAGTCAGCAGACAACAGTGGCACTCTACGGGACAATGGTGCTTCCCAACATGTTACTTCATTGATGTCAGGCCACAGTGTGGATGGCCTCAAAG
GAGCAGCGCATGCCATGTGGAGGGTGCGTGAGTTCTTCTTGATGCAGGATCCTGAGACTTTCCCTGACCCGACATTCCTGCAGTTTAGTATAAT
GTATGGAATAAAGAACATCTTGTCCTTTTCCTGACGTCACCTCCCAAATCCTCTGCTCTGTGGGGTCTTGCCGTGTTGGCCCAAGTTGTCCTGG
AGGATAGGTTGTGGGTACTAGGTATGTACGGTACCAGCAGACATTGTATCTGTGACAGTTCACATGCCGTGTGTGTGTGTGGGAGTGGCTTGGT
TCATTCTTTTCTCTTCCCTCTCCCCATTTATTTATTTCCTGTGTGTTTGTACCACCACACAGGGAGTGGGGCTCAGTGAACGACCTTGAGGGGT
TCTTTTCTTCCACCATGTGAGTTCTAAGGAACAAAGCCAGGTGGTGGGTATGACAGCAAGGGCTCTTACTGGTCGATCCGCCTTGCCAGAGCAG
GGCTCTCAGCGTATGTTCCTTGGAGGCTGCCGGCTATTTTCCTAGTGGCTCTGGTGCTGTCTAGAGCAGGGCTGATTACTACAGTAAGACGGAA
ACTTCTACCTTTACAGTGCTTACATAGAATTTGTGATAAAACTTTAAGCCCTAGAAATGAACTCAGTCTATGTAAGTTGGAGACAGTTCTGTCT
TTTTGTCCTCTTAACTTTGAGATTCGGCTGTAGATGTTGGGAGACAGCTCCTGCCATCGCAGTGGGTCCTGCCTGCCACCCTGTAGGTGTAGCT
GTTCTCCCTCCCCAGGGCTGGGATGGCCTCTGGTTTTCACACCAAAAGAAGTAAATGTGAGGGGAGGGTTATCCTGACCACATTACAGGTCTAG
GAATGCAGACATTGCATCTCCTGGGAGGGTAGTCTGCTGCTTGGGGTTTAGAGGGGTTTATTATCACCTGTTGCTTTCTTCCACACTGTATTTT
GCCCCTCATGGACTCTGTAAATCTGGGTGACTCCATAAAGAGTTTACTATCTCTAGGACACTCAGGATTTGAAACCACGGGTGGACCCGGTTTC
TGCAGTGGCTCTTGTTTTTTTGGTGTGCTGGATGCACAGCCAGTGGTGGCGTTCTAATTGGAAAATAAATGAGGCCGAGTTGTAGCTTCCTCAG
TCTGTGCTGTCAGCATAAGCAAGGCCTTTCCTTATCCGTGTTGAGAGGAGCCGTATTTTTAGAGTAAGGGAAGGAAGTGAGTCCGAGTCGAAAG
CCTTGCCCGGAGCTTTCCGCTGTAGCTGCTCAGAGCAGGAAAGATGGCAGTCGGCCTTGGCAAGTCTGTGGCCAAGTCTTGAACTACACAGCTC
ACCCTGGAAGCATCCCAGCGGGTGTCGCTCCTGCTGCTGCATTTGGGGTTGCTACTTCGTCTAAGGCCAGAGAGTGGGAAAAACTTTGTGGAAC
CTCAGAACCCTGGGAACACTGACGACTCTGGACTTTGTAAGGTTATCCCAGACTCTTATGTTAATGGTGGAGTCCTGATTCGTTCCAAATTAGA
TCAAGGGCATTTAACTGTCTCCTTCCCCTCTGTAGTCACTGAGCTTTAAATTCTAGTGTAGATTGTGATTCTATTCCCCTTTACAAAAGTTTG
ACTTTTCTAAGTGTAGAAAAGCAAAACTGGAAACAGAATTGTTGGCTTATGTCTTGGGACAGGCCACCCCACTTCATCTCCTTTTTTTTCAATT
ATTAGCTATTCGTCCCTGTGAAATTTATCTGATTCTCACTTCCCTTATTTGAAAAAACCAACAACAGAGCCAGTCATAGTAACTAACGGGGTTG
TGAGGATTAAATAAGCTTAAGCACCGGGTGTCTCTCTCAAGGCCGAATGCAATAGTTATTACTGCTGTGCTGGACACTGGAGAAAGGAGGAACG
TTGGTCTAGCAGCCCTGAAGAGCTCACAGTCATTCCGAGGTAGCTCTACAGTGAGCAATTAAATAAATATGTGGTGATAGATGATAGGATGTCA
TTCACATTGAACGACAGAGTAGCTGCCCACAGCAATGGGGCTCTGGGCTGAAAGCCTTTTTTTTTTTCCCCCTCGAAAGCACTGGTACTGAGTT
CAGTGATTCACTTTGGGCGAGGTTGTTTTCCCCAAGCTGTTTAGATCTTGTTGCTTCTTGTCAACTTGGTTTCCTGAGCCTGCACTGCCCGCTC
TCATCTTCCAGCTCACATCCTCCTGCTCTCTCCTTGGTTGCTCTCCCTGTCTGTCCCTATGTGTATTATGTGGCTTATAGTAAGAGCGTGTGTG
CTGAAGAGTGCTGGAGTGTACTGGAGTGCTAGCCTGGGCATGAACAAAATCGAGTGAGGACTCCCCACTTCCTCCGCTGCTGCCTCATTAACCG
GAAACGCCGACCTGGCTGCGGGGCTCAGTGGACAAAGGTTTGGCTGAGTACTTGATGCCTCCTCCATGCTTTATGTGTGACAGGCAGTCTTCCT
CACGTTGCTAGAAGTGACAGGAAGAGTCCTGTGACAGGTCTGTCACCTCCCTCACAGGCTCAGCTCACAGGGAGGAGGTGCACTGATTGGCCAC
TGTGATGGAGTTCTCAGCCACAGTAATCCCCTTCCACAGATGCGTCATGCGCCCACATGTGGCAGGGCCACGGGAGCGCTTCGTGGTGACTTTG
TTAGAAAGATGCTGTCAACCCTTTGTTTCTGTTCTGTCTGCCCCACCTCCGATGCGATGACCATTTTTGCTAAGGAAAAACTACTACAACACCC
TCTAATGCTGTGCCAGGAGCACAGTTGAGCTAGCTATTTAGTCCTGACCATGAGAACTCTCAGTGCACTTCATCTTCCTGTGGTAAAGCACAGT
ACTATAGGATCTTGAGGGCATTGTACGTAACCTTCACTGACTGTAAAACCTCGAGCAGCCGCAGCCACCAGAGTTAACAGTGGAACCACGTGCC
ATTTAAACTTTCCTAGCGTGCAGGTAAGAATGTGGAAAGGCACAGGTGAATTTGACTTCAGTAATAGGGCTTGTTTATTATTAAAGCCACAGCC
AGCTTCGGCATCACTCTAAAATGCCATTATTAACCATTATTTCCTTTCTAAAAAAATAAGTCCATGGTATGATGTGTATTTACAGTACATTTTC
TTCACCTTCTAAAAGCCCAGTAGCCACATATAGGCAGAGTGGATTTAGAGAATCTAACCTTACCTCTCTTCTCAGCCTTTTGGCTAAGGTTGAG
GGGTATGAGAATTTGACTTATAATTTAATGAAACTTTGTTCAATACTTTTTCCTACTCAGTGAATTTTAGATGTTAGATTCACAGATTAGCCCT
TTGGGTAGGACAGAGGCACTGTGCTGTCCTTCGTGTATGGGAGAAGGGAGTTTGCCTGAGTCTTTTGTGTGTGTATCGGCATGTGTGGGGTGGG
TGGTGCCTGGGAGTGCTTCCAGGACAGGTCCAGGTGGCTTTACTGTCCAAACACAAGGCCTACCCACTTCCTTTTGCGAAGGCACAAACTCTTC
ATAGAGTCTTGAGGTTAAAGAGTTTTTCTGAAATAGAAGGTTTTACTTGAAAGGACTATTCACAGCTACACAGGATAACAAAGCACTTGGGGTT

CCCACCCATTCCCCTCCCCCAGACAGATTTCGAAGTTGGCATTCCACAGAAGAGTTGACTCTGAGGGTGTAGGTTGAAATGGTCAAACAGATCC
AACAGCTTGGCTTTGGATTGCTTCTGTGCGCTTAAATTAGATCCCGGAAGGCCCACATTTTCTTGGGTCGGAGTATATGATGTGATGACTATGG
TCTCCCTGGAAGGAACAGGCATTGCAGGGCTAGGATAATTAAAGACTTCCTTAAAGGAACGAACAGGTATTCTCAGCTTCTAATTAGAAAGGTC
TGTACTGCACATCACTGCAAATTAAAACAGCTCGGTTGGAGAGGAGTCTGCACTGGCACAGCCCGACTGCCTTGTTTATTTCTTCTTTTCCCAA
AGGATTGTTTTTCTCCCTTAAGTTACGTAGGGATTAGAGAAGCAGTCAGGTACACAGGGTTATCAACAAACAACCACAACTAACTGTTACGTTA
TCTTGGTACTAATTGTGTCTGCGCAGTGACAAGAAGTAAGCTGAAGCCACCCTTTGCAGCCTTCAAAAACAGGCTTGGGGTAACCTGCTTGCAC
TGTGCCCCGCCCCCTCTCTTTAAATGGTCTTTAGTGAGGAAACCCTTTGGAGACCTTGAGAAACTTGGAACCATTGATAATAACTTTGCCATTA
GGCTTAGAGAAGCAGACACACTTTGAATGTCTTCAGCACATTGATATGCTGAGCATAATTGTAATGTATTTTCCGTGTGTTCAGAGAAGCAGCC
ACACCCCGAGACTGTGGGTGTCAAACAGTATCATAGTAATTTAGGAAGCACCCACAGACTCCCAGTGGGCTTTGCACGTAAGTCTGTACCCTGC
TTTTCTGGCATTTGAATAGTCTAAAGTTGAGTAGGTAAAGCTGCTGAGAGCATGTTAAATACCCTAGAGAAGTAGCTAGGTGTGAGAGCATCTG
CAGTGTACTCAGAAGGACAAGCCTCTGATGAGCAGGCAAAGCAAAACAAATCACTCTACCCTCCCGCTCTGAAGCAATGTGATGGACTTATAGA
AGCTTCAAGTGTCCATATAATTCATATTTGTCACTCTTGATCCTGAAGTTGGAATAGCAGGAGACTAGAAATAAAGACTGCAGAAACTTACCCA
GGCTGGCGGGATGATGCAGCAGGAGAGGGTCGTCAGCATTCATAAGTGTCTGTGTTCATCGGGGCTGCCGCTTCAGCCGCCTGCTGTTCCTGCT
TCTCATCCCACTTTTAAAAAAGCTTCCCTTGATGTGTGCGTGTGTTTTCATAGGTGTACATGTGTGTATGTACATGTGTATGTTTGTATTTATT
ATGCATGTTTGTGTGGGTATGTATGTGGCTATGTATGTTCCTGTTTACAGGTATGTGTTTGTGTGGATGTGCACATGCATGTGTGTGTGTGATA
TGTTTGTGTGAGTGTGTATGTGTACGTGTATGTATATCTGTGCATTCATAGTGTATGTTTGTGGGTATATATGTGTGTACGTACATGTGTATGT
GCACATTTGTTTTGTATGTTTGGGTGTGTATGTGTTCATGTGCATATGTATGTGTGCATTTATTATGTATGTTGCGGATGTGCACATGCATGTG
TGTGTTCATTATATATGTTTGGGTGTGTATGTGTGTATGTGCATGTGTATATGTGTACTCATTATGTATGCTTGTGTGTGTGCATGTGTGCACT
CATTATATATGTTTATGTAGGTGTGTATGCATGTATGTGCATGTATATGTGCACTCATGTATGCTTCTGTGCGCATTCATTATATATGTTTGTA
TAGGTGTGTATGTCGAGGCTGAAGTGGGCATCTGGTGTTTGTTTTCCATCTTATCTTTTTGAGACAGGTTTTTTGGTTGATTCTGGAGCTTGCT
GGTCAGACTGGCATGGCTGGCCACACCAGGAGTGTTTGGGTGGGTACTGGGCATCTGAACTCAGCTTCTCCACTTTCCTGAGCCGTCTCCGCAG
CCCATCATTTCACTTGCTTTCACTTGGTAGTTCGGCTGTCTCCAGGGCTGACAGCTCGGCTCTAGCAGCAAGACAGGAGGCAGTGCCACATCCT
TGCATGACTTGAGTCCCAAAAGCCTGGCTGGGGTGATATGGGAATGAAGAAATGCAGACACAGGGACACAGAGAAGGTGGGATCAGGTGCTCTG
GACGCTCGGTGTGTTCATTAACTACATTTAAATGGGGAAGCAGGGTCACTGCATACAGCTGGAAAAGGAGGTGAGGCTCTTAACATCCAGATAC
ACAAGGGGCCATGGTTATACACAGCTGAACACAGAGGTGGCTTTAGCTGATCTCTGCAGGAGCAGCACCTGGAGGGGAACAGTCTTCAGGCTGT
AAACATCCAGGGGGAGAAAGCTACACAGAACCTACCTGTTCTGCACACACTGTCTACATTTGTACTCAGGGCCAGGGCAGGCTTAGCCTTTGCC
ACTCCTCTGAGCCTCACCTGGGCAGCTTCATCGGTTCTTTGGGGCTGAGACTTTGGTCCTTGTCATGGCCAAGTGCATGCCAGTACACACACTT
TGGACTTCCCTTGCACATGGCATTCTTTCTTGTGTCACTCACAGCTCCTGAGTGCCCTGGAAAGTACGACAGACAGAGCTTTCATAACAGTATT
TGAGTCATGAGTTAGCATTCTATGAAGAACACTTGAACACACATGCATGCACACATGTTCTTATCAGGAGAAAGAAGCATGAAGTCAGCTAATA
GCCGAGGCCACTGGTAAGCCCTGAGTGGGTTGTGTGTAGACCACTGCCTTCCGGATGCTGTTCCTCCTCTCCTGCCGTGCTGACGGGGTAGAGA
GGTAGAACTCCAAGAGTCCGCTTCTTTCCTGCTTCCCAGCCACAGTGAGTGATTATTACAAGTAGCACAGCCTTAGACCTCATGTAGGGTCTCT
TGTGCTACTACTTGTCCTTTTCCCCCAGCACGTGTTTATTGGGCTGGTGCATTGCTCTTTCCATTTATAGAATGTTTGCCTTTACGAAGCAGAC
AGTACAGTGAGGTAAGCAGGTAAACAGGTACAGAGCAGATGATTCCGTAGAGGGTGTTGAAGCAGATGGTAGTTTCATTTAGGCATAGGAGACT
TGTTAGGGACTTAAATGCCATCCACAAGGAACAGAATTTAGCAGCTGTAATGTGAACCTTATTTTCTTTAAAAAACACAGAAATACAGTTATGT
TTTTTAATCCTTGGTGTTGGATATGAGGATGCTTCAGATTGTCCACAGCAGCTGACTATGATTTGCCTCATGCTCTAGCAGGGGTGCGATTTTT
GCCAGCCGAATATAGTTTTTGGGATTGTTTGATGTTTGGAATTCTGGGGACTTTTTAGACCGTATATAAATGCTGGGGCCCCAAGAAGTGAGTG
GTTGTTTTTGGTCACCATTGGTTAAACAGTTGTGCACAAAGAGGAAACAAGAAGAAATTAGCTGTCCTGTCGGCAAAGATCAAACTTGCCCCAA
GGAACTTGATGCCCTTAGTCAGCATGAAGTAGTCTAATGATAATGTCATCTCTTTCCTCTCTTATCCTTTGTTTTTTCACTCTTATCTAGTGTT
ATTGGGTCAAAGGGTGGAAGGGATAGAGAAGGGTGAGAGAAAAAGCCCACAAAGTAGCTAAAGTCTGGCTCCAAGAAGCTTTAACTGTGTGTGC
TGTTTTATTTGGTCTCCCTGGATTTGCATGCCTACCCAGTTTGTTTCTGTGTAGGTGGTGTGCAAGCTGGGAGAACTGAGTACTGAGGAGGACC
ACAGGGTGTAGAAGTCTAGATGCTGAGTGAGGAAGAGATTTGTCATGGACTCAGAGTGTCTTTAAATGTCATTTCACGCACCCAGGATCTTTGC
ACCTGGAGTGGATACATTTAACAGACATTGTATACCAGTCAGAGGTATCTGCACTCCCATTCAAGTTGCTTTGCTTTCTCTTGTGTTTTGACTC
AGTGTTGATCTGTAGGAGCCAGCCCAGTACTCATCATCCAAGGTGAGGTGGGCTGTGGTTCTGGGGATTCCTGGGCAGTGTTTTTTTGTATTTT
AACTGTGAGTAGTCTCGCCCTTGGTAGCACAGGTGACCTTGCTGCTGGCCATGTGAGCATTCCTGGCTGGTAGCTAGCAGCGCTTCCCTATGTT
TGGGGTGCTTACAATTCTTTGATTCGCCTGTTATGCTTTCCCTTGGAGCCATCCCTTCCTTTCAGAGTTCTAAAGTGGATTGACACTAATTTCT
ATCCAAGTTGGAAGTCTGATGGCATGCAGAAGGGAAAAGAAGTCGGTTCTGGAAATCTGGCCTGTCTGTGAGTCATCGAGGGGAGCCCAGGATC
ACACTGGGCCACAGGTGTGAGTTTCTCAGGCTGTGTTCCAGGTGCCCAGCACTTGGTTCAGTGACTCATGTGCCTATCTGACAGGAGCGATTAC
CTCACCGGTTCAGTTTTGCATTGGTTCCTTCTTGGCTGAGGGGAGGATGCCACTCCCCTATTAACCTGCATTTTCTCATGTTCTGATTCATTTA
ACACTCCACCCATTTTCTCAAGATCTCGGTTGACAAGCTTATGCCTGTGACAGAAGCTCCTTGATGTTAGTGCCACAGTAAAGAAGTAGGGAGA
GAAACGGGATGATCCGGAAGGTTCATGGAGGTGTGCACTTCTGTGCCAGCAGTCTTGTGTGCCAGCAGTCTTGTGTGCCAGCAATCTCGTATGC
CAGTAATCTGTTTGAGATGTCATACTCCCATTGAGCTGAAGAGAACATGGGAGACTTAATAAAGCTGAGTTTCCTGTCTGCAATTATACATCCG
GAAAGGTGCTGGCTTGGAACTCAAGGTCATCCCACTGAACCATACTGAGAATTTCAACTACAGTCACATTCTGTATTGTCGCCACTGCTGGGCA
CTGTCTTGTGTCTGCCTATAAAACCATGCCAGCCGAAGCACCTCTGTTTGCTTCTCCTAGTGCCAGGTCCTGCCTTGGAAGCCTTGTGCAGATA
GAGTCATGGCACCCAGGGCAGAACAGAGCTTTGGACAATACTGGCTCTGTGTGTGCTGTAAATGACGGTGGTAACAATGGCATTTACTGTGTGT
TCTGTACATGCTGCACGTTGTGGAGCATGCTTCTGCCTACACCGCCTCAGTTCATCCCTCAGATTATTGTCCTGTTCTTAGGTAATGAAACTTA
GGCTTAGAGCTGTTTATGAAGCTTGTCCTACTTCTTACAGAGCTAGGTGGTGGCAGGGTTGTGAGTTGAGATCTTTGAAGTAAAAGTGTTTGAA
TGTGCTTTGGTATTTTGCACACATTTTCACTGTGCTGCTGGAGGAGGCTGTGGAAATGCATTCCCTGGAGAATGCAAAAGGGTGTGTGTGTGTG
TGTGTGTGTGTGTGTGTGTGTGTACATACGTGTACGCTCAGGCATCTTTTTTATCGAGAGGTTGAAGTCTAAGAAATGAAGTTACTGAGTGGCC
TCACGGGGCTCTGACCTGGGAAGCAGTTGTAGGGATTTCAGCAAGTGTGTGAGCCGTTTTAAGGTTTTTGGTTAGTTATCTTTCTAGCCATTTT
TGTCCACAAGTACTCACTGAGTCATGTTTGACCCAGTAAAAATATCCCAAGTCTAACAGATGATGTATTTAAACAGTAGTCACTACAGGAATCC
CCAGCACTTGCAGGTGCTCTGCTGTTTGCTTTGGATTTAACCACTTGTGCTTGATATAAAGTCCCCTCATTCTTGAGGCTCAGAGAATTAAATC
ATCTGCGCGATTTGGTCTTCAGCCAAGCATCTTGTCTCCTGTTGGGAGTTTTTTCCGTGGACTTTCAGGTGTTGCAGATGCCCTACGCTCACCT
TTGCTCATGTACAGAAGGGTCGAGCTAGCAAGAGAGGAGCCAGGCTCTGCTTAGCCCCAACAACCTTGCTCTTTTCATTGTTACTTACCTGCGC
GGCCTTTGTTTTCTTCCTGTGAGTGAGAGCCTGTGGCGGGGCATTTGTTAGACTCCACTTGCTGCTCAGACCCAGGAGCTGGCTGCTGGCACAT
TTGTGAGAAACCACTTTCAGGTTCTGGGTGATGCCCTTGTTTTCCTGTGGGAGCTAGAATTGTTGATTTACGTTTGCACCAAGCAGAGTGAAAA
AAGGCCCAAGGACAGTGCTGTGGGAGAATGAGGCTGGCATTTGGCTGGCACTAAAAACAGCAACGTGTCTGCGCACTCAGCAGCAAGCAGTGGG
AAGAGAGAAAGAGCCTTTTCCTGTTTTACTCATTTTCAAGAGGGTTGGAAAGAAACCCAGAAGAAGGCTGTATAATACAGTGACGTTAACCAAG
ACTGACTACCGAGTTGTGTGTTGTATTACAAGAGTGAGCAGCAGGGTTAAATGGATTTGTTTTTATTTGTGCCTTGCCCTAGGGAGAAAATAAA
GATGGGTGTTTACTGTTGAGTAATCTTTTCCTTCCTGCCTTGTATTAGTTAACAAAAGTTACTGAGGAATTTGCCTGTACTAGATTTGTGAATC
ACAGATGACAAAATGACAAGATTTTGTCAAATGCTTTCTAAAAACGGAATTTGTTTTGATATATATATATACAATATATATATATACAATATAT
GTATATATACAATATATGTATATATATATATATATATATATATATATATAAATAGAAATCTTTGTGTTCTGGCCTAAAATTCTTGGACGTTTTC
TTGCAGGTTTCCTAGTAAAGGCTATTTGTCTGTCATGCAGACTGAATTCACATGGGCAGGACTAGGGAATAAGTGCTTCAGCCTGACTAGAGGA
CACTGTCCTACTTCTTGACTCTTATTTCCCACCCTCCAATTTCCCATCTTTTGGACTCTGCAGGAGCGGAAGTCCACACAGCTAGTGCTGGGCC
TAGGACACTGTTGGAGGAGCTGAAGGGCATGAAGTCTTCCAGACCAAAGGCTTCTGGGCCCTAGTGAGTTCTCATCCTCACAGTCGCTAACCGA
GTTTGGCTTCTTCCGTGATCAGGGCACTGTTGTACAGGACAGAATGTATAAACGGGCATTCGTGTTTTCCTTGAGAAGTGTCTGTGTTCCAGGA
GTCCTGCTGGCCACGCACCTGTGCAGGAGGACGCCATAGCCACGACAAGTTCTGAGTTTGATAAGAGGGATCATGAATCTTTTCCTTGGCCCCC
ACCCTGTGGCCAGTAAAGTGCAGATTAGCAAAGCCTGCGTCTCAGCTTGTGTCCTGAGACACCTCCTCCACATCCCACCTCTCTTAAAGCAGTT
TTAAGGTTGTTTGTGGTAATAAGCTCAGTTCGTTTGTATTGTGAGGGAGACTTTTTGAGGAAGGAGAACTCCCTGAAAGGACTTGATGGAATCT
TCTGCATTTATAACCACCTTTGCTCTCGCATTGGCCATTTGCCCTTTCAGTATTAGCTGTCCCTGAAGATTTGGCCGCACCAGCTCTTAGTGCT
GTACAATGTTGGGAGTAAAAATATTTGCACTGTGAGCATTTAGGGGCTTTAAGTCATACATACACACATCATAAAATAGCAATTAGCATCTTTT

CTTAGTTGACTGCACAGAGGCAGGAATTAAACCCAGCCAGTGCTGCTACAGTTCTCTGCTGTGCTGGGAAGTGGCTGTCCTAGTGAGCCTTCCC
AGGCTCAGGGCTTATTCATGGAGCTGGGGAACATTGGGTCTTCGCTGCTGATCCTGGCTGCCTGCCTGACATTTCTGGGCACGTGGGGTAGAGC
AATGCCCTCAGTGTCGCTGGTCTTGGGGAGGTGGTGCTTTGTCGTCTTGTTTTCTGTTTTTTTTTTTTTTTTTTTGTGGGTAAGGGATAAAGA
GTCAAGATTCATTTGTGTTGGGAGGATGCTCAACGCCGAAGTGAGCAACTGTTGAGTAGTAGAAAGGAATGCCATCCCTCTGCCTACACATCAT
GAGGAGAATAATGGTTGCCACCTCTCTCTGTTTTTAACCCATCTTTGTCTCAGGAAGTCATGAAGGAAAAAAAAATCACAGAAAAGGAAAAAAA
AATCATAGACCCTAAATGCTGCCTCCTGTGAGATTTCATTCCGGGCCTAGCTGCCAGCACATCTGGAAGCTGTGGAGAGCCAACTAGTTCAGCC
TGTTCTTGTTTGTGCTAGTGACAGGGTAGACCCCATGCCCATGTCCTTTGTGCTTTGTGGTCCTCTTTGTCACAGGACCGCCTCGTTTACCGCT
CAGGCCTGCAGTGCGGTGTTAGCACTGACTAGATCCAGTTGTTACTGGAGGTCTAGGCAGAAATACTGCATATAAGCCTTGTTTCTATGTCTGT
CCCTCTGAAAAAGTTGAGAACACCATCCTAGCAAATATTGTGTGACCTGCTGTTTGCAAATAATAAAAAAAATCAAGGTGTGAATTCATTCCAG
ATTTTTTTTTTTATTCAGTAAACCAAATGTAAAGTGCTAGTAACCTGATCATTGAGGATTTTAATGGGAAAATAAAGCTTTATTCAAAACGGTC
TGCCGCATAACAGTGGATTTCACCATAGAACTCATTGTCTTGGAACACTGTAAATCAGAAGTTTTGTGTGTTGTGGTGGTAAGGCCATCTTACA
TCCTTTTTGGGAAAGGGATGGTGCAGAGAAACTTGAGGACACTGTCACTTTTCACTTTGTCTTCTCCTCACTGTCTATATAAAGCTCCCCAGGT
CTCAGTTTGACCACCCACTGTTTCCTTGGTGTCCTTGGTCCTGAGGATCCTAAAGCTTGTCCTTAGATACATGCTTTTCCTAGTGTGAGGCAGC
AGCTGGTCTGAAGGGAAGGGAGGAAGACTTGAGAAAATCAGCCTGTGTTAGGAATAGGGACACCAGAAGTAACCACACACCTTGTCAGCTTGTT
CTCTCAAACGATCTCAAGTTAAATACAATCTGTCTCTTTCAGGGGACCCCTGCTATAAAGGAGGGGGTGCTATTTTTTTTTTTTTTTAGGCTTCT
TACTTGGGGCATCTTACAGAGAATCATTGAAAATGATCTGTTACATCTTTCTGTGTGAACAGAATTCGCTGCCTTTGTTTTCCTTGGACACTTT
TCTCCTAGAAAGAGCTCTTAGATATACCCCAACCCACCCGTTTGGAAGATTCTTGCATAGCCCCACCTCTCTAGTATTTCCTTCAAATTTGTAG
ACTTCTTAGGGGGACAGAGGCAGGACACTGTTGGTGACAGAGGCCACAGCAACAGCCTTCTCACTAATTCCGCCCCCTTTTCCTTTTAAGGATT
GGCTCTGTTTCAATGAAGTTTTTTTTAAGAAATGACTCCCATCTTCTTATGCTAGGTAGAACCTTGAGTTGACTCCCGGTCATAATGAACAACA
ATATTATCTGGGTTAAGCAACCTAGGTAAGTGGAGGTAAAGTATGATTAGAAAAATATAGGTTAAAAGAAAGTCAGGCTTAGAACTGAGAGTCC
TTTAACTGGATTAATCATAAGAATCTATTTTTAGACCTTCGCTGGCCTCAAACTCACAATGTGGCTGAGTCTAGCCTCGAACCCTTGCCCGTTC
TTCCTTACCCAGCATGCACGCTGACTGGGACTCCCACTTAGGGAGGACTCAAAGAACCAGTTGTTTTTATCTTTGGAATTGCTAAGTCTTCTAG
AAGCAAATTGTTGGGACAGTAAGGTTTCTAAGCGGCTTTTGTGATTTACACATAAACCATAAACAGTAAATATCCTACTGAAGCAGTAAATGCA
CTGCAAACAATAAATATTCTACAGGAGCAGAATAGCTTTTCTTTGGTTTTGATGTAAACAAGGCACTTTGTTTTTCTAATTACATTAATGTAGA
GTTGAGACTACATTTTTTTTTTTTTTACCCCCTCCCCTTTCATGATCGGGCAGACAGTGATGGTGTGGTTTTTATAGGGACACTGTGAGATGTT
GCCTAAGTCGTTGAGTCTGTGTGTCTGGCATCGTGGGTGCCACATCTGTCATCTGTGCTGAGTGCACACAGATGGGATCTGGAATTAGGACAGG
GTAGTCAACAGTTGGAAGCAGTGAGCGCTCACCCTGGTGAACTCCCTGGACCCTGCTGGCTCGACAGGTCTGTAGGACTGGCCAACTTAGACTC
TTTTAGTTAGGTTCTTGGCTGGTAAGATAGCACCAGGAGACCCTGCCAAGCAAGGTCTCCTACTAGAAAGTTTGTGATGGGAAGTGAACTTTTA
ACTGAGCGTGATAAACGGGGTTGCTGTGACCCAGGCGATGGCTTTAACACCAGGGCTTCTGTGTTTGCCAGTTAGAGGTGCCTCATCTTATGGG
TCCAGGAGAAAGAAACCCTGATCCGGAATATACAATATGGTAGGCGTCGCGTGTCGGTGTGATGCTTAGTCTACGCTGGCTTCAACTTGGCAAG
TCATCCATGGTTGGCACAGGTAGCGCATTTGGTGCGCTCCCCTCCCCCTTCCCCTTCTGGGCAGTGAAGATCTCCCCCTGTGTTGACTTGAATA
CTTAGGATTTAGAACAGCAGAGGAGCGTTCAAAGGAGGCACTTGTGAATAGAGTCTCTGTCTGGCCCAGGCCCTGCTTCTCCATTCAGCTCAGT
ACAGAGGTACTTTTGATGCCATCTGCTGAGTTGTCAGCTCTTGTCGAGGTGAAACAGCTGAAGTGGCACAGGGGGAGATTATTAAGGGCACGAC
CATCTAAATGGGAGCCCTTTAACAAAAGTCTGGCAATAAACATAATTTCCTCATTCTAAATTGCTGTGGAGACCCCTTCCCCCCCTCCCAGCAT
GTGGTCCACAAAGCTACTGTTGTCTCACTGGGGCTAGCAGAGGGCCAAGAGGTCTTCCTCCTGGGCTCAGCAGGCACTCCGCCCTCCTTCTGGT
ATGATTGGAGGAGAGGATTATGGGAAGCAGTGGTCTCTTTGATGCTGGTTCTCAGCAGTTGGTGTGGGTGGGTAGGGGCTGGGATATGATGTGC
ATGGTAGTGAATTTAAGCAGTCATCATGTATGGAAAAATGGCAGCCAACCATCGTTTGGTGGGAAGCAGTATGTGAGAAAGAGAGGCCAGAACA
GCATAGAGGCAGGACATCCAGTTTGTCTTCACACACCTGCCTTGGATTGAGACTAGATAGATGCTGGGGCTAACTCCACCTAACCTCAGCACAG
CCCCAGAGAAGACTCCCGTGAGGTGGGGAAGCATTGCTTCTCTACCCACACCCTCAGCTCCCGCTTCAGCAAGTGGACGGACGAGGCCCCTGTT
CTCAGAGCTGCTGTAGGTAGGATGCAAGTTGATTTGTGGGTTTGAGGAGTACAAGTCCACCCATTTGTTAGCTTGTGTTTTTATGAATTGCAGC
AGTGACTGGTTGCTGTGTGGAACTCTCACACCCTTTGGTGCTATATGTTGGTCAGAGTTTTTCCTAGGGGTTTTTGGTTTTTCTATGGTGGGGC
TAGTGAAATAACAATTTTCACCCAATTAGTTTGTTCTAAATGGAGAAGAACCAGAGTCGTTTCCAGAGTATGCACAAGTGCTTGTGTTTTCCAG
CCAAGGGAATTGGGTGAAGCAAACTTTTCTAGAGTTTAAAGCAGGTAGGTTACATTCAGAGAGACTCAGAGGAAAAGGAACACACTGGAAAATG
ACAATGTTTCTACAGAAAACAAAGACTAGGAATCACTCAGGATTCCAGGGTGAATGAGAGGTGGAATGCCACTCTAACCTGCAAAGAATTCTTG
TGATATGTTTTTTAGTATAGTGTTACAGAACAATACCAGTGTGCCATCCCAAGGCTGTGGGTGGACAGAGGTGAGGTTTCACAGAAGCTGGGCT
GCAGCTCTTCAGTTCTGTTAGCCCCAGAATGCTATTTGATCAGACAGGACAAGGGCTCCGCAGTTCTGTCCCAGCTTTTGTTTGTCATTTGGAG
CTGTTGCGACAGGGCTTTATTTATTTATGTGGGCAGTCCTGTGGACTTAAACAGGGTTTATTTCACACACTTAGAGACAGTTTGGTGGGCTGGA
GAGATGGCTCAGTGGCTAAGAGCACGCACGGAATGCTCTTCCAGAGGTCCTGAGTTTAATTCCCAGCAACCACATGGTGGCTCACAACCATCTA
TAATGGGATTTGGCGCCCTCTTCTGGTGTGTCTGAGGACAGCTCCAGTGTACTCATACATAGATAAATGATTCTTTAAAATAGTTTGGTTATGT
AAGAGAATATAGTGTGTGTGTGTGTGTGTGTGTGTGTGTGTGTGTGTGTGTGTGTGTCTTGTATTATGTGGCACACAGTCAGTCCGATGGCCTC
CATTATGTAGCAGCACTGTTGATACGCCAGGATCAATAGAGTGTTCCTAACAGCAGTAATCTTAATCAAATGAGATAGACTTCTGGCTTAGAGG
AACAAATTTATCAACACAGGTTGTGCAAGGGAGCAGTCAAATACATTCAAAATAGGAGACTGTATCAAGAAGCTTTGTCGGCTGCCTACTCTCC
TTGAGACTTTGTATCAAGAAGCTGGCCTGCTGTCTTAGTCACTCTCTTTGCTGCTCTGATTACCTTCCTGACCCACAAGGAGATGTTTATCTTG
GCTGCAGTGTAAGAAGGGCTAGGGTCTGTGGTGGCAGGGAACAGATGAGGGTGGGAGCTGGCTGTTTGTACTGTTTACAGTCAGCAAGCAGAGA
GTGGAAAGAAAGGGAGGCCTGCTCACAAAACCCAGGCTCGCTGCTAATGACATACATACTTCCTGCCATGTCTCCACCTTCCCAGACGATGCCC
CCAAGGGCCTGTGTGCGGATTTCACATTTACAGCACACAGTAAAGAGAAGATGCTGAGGAAGATGGCTGAGAAGCAAGCTAGAGTTTGGTGTGT
GAGTGTAGACTGATTGGCAGGTAATATGGGGGACCATTGCTTTCCTTAGCTCTGAATAATTCTATCTGAAAACTGCTATGCAGTGCTCTTCCCG
TCTTTGAGAACAGGTTTTCTTGATGGATGTCTCTGTTTGAAGTGGTTCAGGGAGGAGCTCCATCACTAGGAGAAATAGACCAATAATGTTATGT
TAGCAGCTGTTCAATCTAGACAGTAAACAGTTAAGACAGTCATTACCTATAGGGCCATGTGACTTTTACTTTTCTGTGTTTGAACAATTTAAAA
TAGACAGAATGGTATTTTCCATCCTGCAGCAGTTACATTTGAGGCTGTGTTTAAACTGTAGACTCAGAACGCTTTTAAGGGAAGTTCCCCCCCT
CCCCCACTTAGTTTAAAAAGCCAAAACAAAACAAAAAAATCAAAAATAACAAAATAAACAACAGCCCGAGCCAACCCAAACTCGCTGTAATGAC
AAGGTTTATTTGTGGTTCAGGGCCTGGGCTGCTCTTACTGTTTTGTTGGTCCTGGATCAGTCTCACAGACTTCTAGATTCTGTAGACCTATGCT
CTCATTTTAACTATTTCTGGGCCAGATGCCTTTCTTGTTCAGTAAATTGAACTAAAGTGGCTGCCTGCTCTCTGTGCTGCAAGAATTTGACATG
GAAAAATAGAGGAAAAATTTTACCTCTTCTTTCTGAGTGTGGGATGGTGCCTTCTGGTGCAATGAGGTGCTGGCAATGCGGGGCGGGGCTGACA
CGGGGCAGGGCGGGTTGATGGTGGGGGGCGGGGCTGACACGGGGCAGGGCGGGTTGATGGTAGGGGCGGGGCTGAACAGGGTAGAGCGGGGCTG
ATGGTGAGGGGCGTGGCTGACACGAGGCGGAGCTGATGGTGAGAGGCGGGGCTGGCCGGGGAGATGGTGTCTGTGGTCTTGCAGCCTGGCAAGG
ATGACACTGCTTCAGCAGTTGTCATTCACCTGAGTCGTTTACTCTTTCCAAGATAAGCATGAACATAAATGTCTTAAGGGGATGTCCCCGGGGG
CCTGAGCAGCATCCAGCTCTCCCTCATTGAGATTTGGCCTTGTCCCCGTAGGATAAATATACATGCAATAGACAGCTTTGGTGTCTTCTCATAG
TTGAGGGTCTTTCCTCCCCCCTTTCAGAGTTTTCTTTAAAAATTTAACTACTGGTTGTATCTTTCTCATTTGTTTTCAGCTTTGGCATCTTTGT
CTTCACTTCCCTTTATGTTTCCTGTATTAGGAAATAGTGTTTCTAAACTCTGCTTGTAGGTCAGGCAGACTCTTTTCATTGAGAGATGTGTTGA
CAAGGTCTGGTCAAATGAGAGCTGCAATGGTTTGTTTTAGACTCCTTTCAGAAGTATGTTATCCCGCTGCAAGATGAGTGGAAAGGTGTTGTCA
TTAACCTGGTATTTATTAGATCCTCCCAACTAATTCAGGATTACAGAGTTTATTATTTCTCTAAGTGCCGTTTTCTCCCTAGCCTCTAGTTTGC
AGTTAATTTTCAGAATGAGCCCTTGGCTTATTCTTTGACCAACTTGCTTTGGTGCCATTGGCCTTGGTGTGCCATAGTCTGTAATCTCCAAAAT
AACCGTACTGTTTAATCTCAGGCCTTGGGAAGTTCAGCTGTGGTACTTTTTGGTGTGAGCCCGAGTGTTTCCTTTGGTGGTGCAGCTGCAGGTC
TTTTCTCCAGGCTGCAGCTTTTCCGTGTTGAGAGTGTTTTAGCTGTGGGCTCGGGGCAGCCTCTCCCTCACTTAGGTGCTCCCTTCTAACTGGG
GCTGGAATGATGGTGGGCCGTCTTATCCTTTACTTAGGTATGAGAGTTGCTTTAATGACTGTTGCTGAGCTAGTAATGGCCAGAGTGAGCTGTA
ATATACACTATTAGTACCCAGACCACAGTTCCTTCCCTCCCTGCCCCTGTGGATACTACTGCCTCTTGAGGGCAGGGTTAATATAAAATACAAA
GTATACAGTTGCTTGATATAAAGTTAATTCTGAACTTTTTTTGAATAGCTAATTGAAACATAGATTTTCTTAATTTTTTCATACTGTTTTATTT
ATTTGTGTGGTTTTCCCTTGGTATTTATTTATTACTACAGTTATTTAAGCTGTTTAACCATGTGGCTGTCTTTATAATCATAGTAACGTCATAA
TCATAGTAATAGTTTAATATAATGAATCTTACACATGCATGCACACTCACTCACTCACACATATTTTGAGGCAGCACTTCCAGATATAAGAGAG

GCTTGGCCTGGCCTGGATCCTTCTATTTATCCTAGTCTCCCAAGTGCTGGGATTATAGACATGCATCACCATTTTGTATTTTGAAATTTTTGAA
TTTGACTCATTTTGCATTTTTTACAGAATAAAAATATTATTGTGTAAAACTCTGGCAATGCAGACACTGATTGGCAACAGCTTATCTTCCTCCT
GGCCTTGTTTGTTAAGAAGCTAGGACTCAGTATTCTCCTTTAATCTTCTCCATTACATTGGACTGAAAGACTAACCAGGGGGTTCTTGGGCTGT
TCATTGTCTCTCTGGCTGTCAACCATTGGAACTAAGATTGCTAGAATAGGCAGCCATGCTGGGCAGACAGCGTCACCAAGTGGTATAAACTGTA
CCAGGATCCAGTGGAATTAATTCACCATGTTGTTTCCTGTATTCGTCCCTCAGTCACATCCCTCAGTCACACGTTGTGTTGAAGACTGGCATTG
TGGGAGGGGTGATGAGGGAAGGCTCTGGCCTCCTGGACACCTGCTCTTCCTAATGTAGAGGCGGCAGTCAGGAATGAGGCGGACATCGGCAAGA
GTGAGGGAGGTAGGGGTGCACTTGTGACAGACAGATGGCTGAGATTTTGTGGCTGGTCTTGGGGTGTGATCCAGGTGAGGTCAGCAGTTTCTCT
CCTGGGCTTGTTGCCCTCTAAGCCATGTAACATGGCAGGGCCCAGGCCTTCAGCAGACTCTCCTGTCTTTAGCCTGAGAGTCTTCTGTTGGGAA
TCACAAATCCTGTCTGGGAGTCCATTGAAAGGACATTGAACAAATGTTCAGGTATGGCCCCCGATGAGGATCCGAGGCTTTGCTTTATCTGGTT
TTCAGGGGAGTGTTCTCCACATTCATGTTGGTGTGGGGTTGTGTTTATAGATGTCGCGTGGCTATTCATTTCATGTTCTTTGTGCATTACTGGG
CCCTCCATACAGTTCACTGAAGATTCTAGCATCCAGGCAGGAATTCTACCTGTCTTGGTTTTGAAATTTTCCCATCTGAACATCCTAGATCACC
ATGGACTGTAAAATCAGAATTTCTCCAGTGACGGTGTAAAAGAACAGGAAAAGGGAAGGGAAGTGGGAAGGGAGGAAGACAATGCCCGGAAACA
CTGCAGTCTCTGAGCGGATGTGTCTGTCTGTCTGTCTGTCTTGAGGGCCCCTCACTGAGAAAACAGGAAAATAGAACCTAGGGCTTGGGCACCT
CCTGGGCATTGAGTCATTTCTGCCTCAGTTTAGTTACTTAAACAAACAAACAAATTGTCAAGTAAAAGTGGCTCTAGTTTTCTCTGTACACACA
AGTCCTGAATAGAGTTTTTAAAACATGTGTGGAACGGTGAATCTCTAGTTAGGACGGATCCATCACCCTAAAACCTTCAGCGTGTTCTTCCTTT
GATTTCCCAGTGATAAGAGAGAGCTAGGAAATAACTCCTCTGAGTGCTCTTGGAGGAAGCACCGGGGTGGAGTGAGGTGGGTGGTCACAGATTA
GACTGTCCTACAAGCCTTCTCCAAAGCCAGGCCCTGGGCCTCTCCAGCTAGCTATCTGGGTTTTGTATGATCACATTTTAATAATTTTAATGGT
GACTAGGGTTCCCTCCCCCCCTTCACCCCCCACACCCGTTTTATACAGCTCTTGGTTTTTGAGTGTTGGATTAAGGTTGTAGTTTTCAGTCTGT
AGGCTTTAGACTCCCATGTCAAGGCAAATTCCTGACCTTCCCTGTGTATTGTTCTTCAAGCGCAGCATACGAGGGTGAGTGCATGGGACAGCTG
GTGTCTGTAAGGAACAACAGGGGTGTCTGGCCCTGGAGAGGCAATCTCCCCCCCCCCCCAACTCTTCTTCCTTTCCCTCCTCCTCCCCCTCTTC
CCCTTCCTCTTCTTCCTCTCTCCCCTCTCCCCTCCTTCCCCCTCCTCTGTCCCCTCCCTGCCCTCTCCTCCCCCTCCCCTTCCCCTCCTCTCCT
TTGCCTTCTCCTCCCCACTTCCTTTAGATTGTGTGTTCTGTGAAGATGTTGTAACTCACTGACCATCCCTGGATGGTCTGAAGGGAGGTACTGC
TTACATGGAATTACCTAGAAGGAGAGGTCATCGCAGGGGCTCGAAGGGGCTGGCTCCTGTTGAGCTTGCATGTACAAAGGCAGGTTCCTCTTCT
TTATTATTTGTTGAAAACCATTTGCTGACTGCCCACTATGTGCAGGCCTGGATTGGTATTGATGGTGAGGACTTAGAGTTCAATAATTAGTGAC
TTGCCATCTCTTTTTTCCCTCCTTCCCCTTTTTTTCTAGCACCTTAAATAACAACCACCACTAAGAAACAGCTTCTAGCCTGGTAAAGGGAGGG
AGACTGTCCCTACCTACAGTTTGCCCATGTCTGCATATGTCTTGTCTCTGGGGTCCTGTTAGCATGGTTGGTTAGTGTGAATGAGAGGACAACA
TCAGGGCTCTCATGAGTGCTTTGTCAGTTCTGTCAGAATAGGGTCTCACTGACTGGAGCTCACTGATTGGCTGGACTGGCTGGGCAGTGAGCCC
CTTGGATCATCTTGTCTCTGCTCACAGTACTAGTGTTCCTTCTGGGTGCATGCTGTCATGGCCAGCTTTTGACATGACATGGGGACCAGCTTCT
CACGAGGACCTGAACACACTGCCCAATGTGCTGTCTCCCACCTCTCTTTTTTACAGAGAACTCTGTACTTAGAGTAAAACACCGCAATCCACCA
GAGATGAATAACTAGACTCTGAGCTCATGCATTTCAGGCACTGTTCCTTGGCGTTCGATGGCATCTGACACATGCTGTACAGGGTGTGTGTGTG
TGTGTGTGTGTGTGTGTGTGTGTGTGTGTGTGTCCGGAGCTCAGGTTGCAGGGAATCCCCAATGATGCCACAAACACCTTGTAATCAAATATTT
GATTTTGGAATAATTTTTGTCTTTCTGTGGTAAGGGCAACATTTAATTCAGAGGTTCAGTCCATTATCATCAAGGTGGGAGCATGGCAGCATGT
AGGCGGGTATGGCGCAGGAGAAGCTGAGAGCTCTACATCTTCATCTGAAGGCTGCTAGAAGAAGACTATCTCCCAGGCAGCTAGGATAAGGGTC
TTATAGCCCATGCCCACAGTGGCACACCTATTAATAGTGCCAGTCCCTGGGTTAAGCATATTCAAACCACCAGACCCCAGATTCAATTATGTTC
CCTTTTTGGGATCTGCACCCACAGTGTAAGAAGCTGTGTTTTGAGACAGTTTCTAAACAACAGAACAGATATGGAGAGTCCAGATGACTTGGCT
TTTATATTCCCAGGTATAATAATTCCCTGAAACGTTCTAATTGCAGGACTGGGTAGTACCTGGTGTTCATAGTAATTTCCCTGGGACATCAGTA
AAAGCCTTTGTATGGTATTCAAGGATTCAGGGTTTTAGAAAGTTTCTCATTCATATCAGTTCCTTAAAGAAAGCATGGTACATAGTGTATTTTA
TTTTACTTTTCTTTGCTAGCTCAGCTTTGTCTTTGATTGTGGAGGGGCGTGGTGTAAGCCAGTAATCATATTGTCATCATATTGGTGGCTTTTC
ACTCTCACCACCAATATTCTTTAGTGTTATTTCAGAGAAGTTGCTTTTGCCAGAAGAGGGAATGATTCAAGATTGAGGTTTCTAATCTTTAAAC
TCTCCAGAGAACAGGTGTTCTGTCTTCTTTATTAAAACACAAGTTCTGACTGGTCTCATAATATGTTACATTCTTAACATTTCCTTTGTCTGCC
TGCTGGTCGGAAGCACTGGCCCTGGGAGACCGGGAGGAGGCAGCAGATGCTGGTGGTAAGAAGTAGGACCTATGGATGAGTTTTGCATAGTTGT
GAGCCTGCCTGCTCTTAGCCACGGGGCGGGGCGTGCAACTGGAGGTCAGAGGGAGAGAGACTATGGCTCCTGCTGCCATGTTTTGTTCTCTGGC
TGGGAGATGTTTAGGACACTCAGCTGTTTTCTCAGTGCTCTGAGCTGGTGACTAGCTTCCTTGGCTAAGAATGTTCACAGAGTGCTGTCTGGCT
GCTGTGTCCCCTTTCCGACTTGCTAATTTTCTTTTTTAAAGACACTTCCTGGGGTTGGGGATTTAGCTCAGTGGTAGAGTGCTTGCTAGGAAGT
GCAAGGCCCTGGGTTCGGTTCCCAGCTTTGGGGGGGGGGGCTCTCCCTAAAGTCACTTCCTGGAAAAAGTGTACTCGCAGTGGAGATTGTGGTT
TTGAGGTTCACAGTACAGAACCAGTTAAACTGGTCTCTGCCTTTGGGATGGATGGATGGTGGTCATCAAGATTGCTTAGTGCTGATCTCACCCT
GTTTACCACAGGGCCCTGGAGGCTCCATGTGTGTGCTCTAGAAACTGAACTCAGAGCAAGGGAGGGCCTCGGAGGTTTCAAGCTGACCTCTGGG
TGGTCTAGATGGAATGGTCATGACGTGTCAGTTTGGATGGGGCGTCGGGTTGGCTCTTGGCTTTCCTTCCTCGTATGAAGTGTGTAAGGGAGAG
GTGAATCTGGGTCTGTCTGGTCTTCGCAGTCTTTTTTTTTACATGTTAAAAATATTTTGAGATTATATTAAAATTCAAGATTTCTCTCCCCTT
TTTTCCACCCAAACCCTCCCGCATACCTCTTTCTTTCCTCTTTTTAAGTTCGTAGCTTCTGTTTTCATTAATTGCGGTTACATCCCTGTATGTG
TGTGTAGACATACACATTTCTAAATATGCCCTGCTCAGTCTGTGTAATGTTAACTCGTGTGTACATTTTGAAGACTGACCGTTTGGTAGTGGGT
AGCCAGTTGGCGTGCTCTTCCCTGGAGAAGACTCTTTCTCCCACTCTCAGCGTTCTTCAGAAGCGATCCTGAAATCCTCGGGTGCACGAGAAGC
CAGCAGACACTGCTTTAGCAAGTCGTGGTCTCATCCTACTCTCTCAGGCAGTCCTCATGCTGGATCTGGCTGGAGCAAACATATGCACATTCTG
TGGGAACAGGAGCAACGGTTTTATGTTTATTTTGAGCTTTGCTTGTTTACTAGGGAAGCGTAACATCTCTACCTTAGGTGATTTTTTTTTCTTT
TTTTAAAAAAAGATGCGTTTGTGTGCACATGCGTGTATTCGTGTACGTGTATGCATACCAGAGAGCATGTTAGACTCCCTGGAGCTGGTGGTTG
TGAGCCTGTATATACAGGTTCTCTGGAGGAGCAGTCAGTATACTCTTGACCACTGAGCTGTCTCTTTCAACCCCTCCATAAACTTTTTTTTAAA
GTAAAATTGTCTTTGTGTGGTGCATGTTCACACGTGTATGTGAATGAGGGTGCATTTGTCTGGAAGTCAGAGGATAGATAACCTTGGGTGCTGC
TGGGTCCTTGCCTTCTTCCTTGAGGTACAGGATCTTTGTGGATTGTGGATAGCCAGACACAAGCTAACTGGTTATTGAACTTCCACAACTTCTG
TCCCTGCCTTCCTCTTGCTGTAGGAACACTAGGCTTGCAGACATGCTCCATCCAGCTTTATGTGAGTACTGCACATTCAAGCTAAGTTCTTCTG
ACTTGTTATCCAAGCCCATTCATCAGCTGAACCCCCCCCCCCCCCGAGCATCACTCAGATGTCTAGGGACAGGTTTCTGATGAAACAACATTGA
TAGTTGAATGCAAATGAGAAGCAGTCAGACCACGAAGAAATCTCTCTAACGTCGTGAGAATCTCTCTTGGTTGGGTGCAATGTGTATGACAAGC
CATTCGACTCTAAGAGTACAAATATTGCTTCTCTTATAAGATAAATACAGTGTTACTCCACACCGTGTGTGTACCACTACCAGTATGTGTAGAA
TCATCTACATGAAGTTTCTATTTAATTTGGCTAGATAGGATACCTCTCTTTAACCTGCACACCTGGTAAGGGGACTGGGTCATCTCTAAGGTTT
ACTTTCTCTGTTTTATAAGATGAGGGTGGTGGTACTTGTGTTACATTCCATGAGGAATACAGGCGTTTGTCCAGGGCACACCTGTCCACCAAGT
ATCCCCAGAGCTGGGAGGGGCTTGTGTCTGTGCTCTGCTCTTCCCGATTCCAAGGCTGTGCAGGCCTTATTCAGGTTTGCAGAGCTGAGGTCAC
ATGACCTGGCACCTGTCTGTACTGTGGGTTAGAAAACACTTATTTTTCCCACGTGGAGTAGCTGCACACCTAATTTCATAAAGTAAATAAAAAC
TTTGTGTATCTTTTGTGTGTTTTGAGGTTGAATGGTGCCAAATGACAGAGGGACACAGACTTTAAGGAAGCACAAGCTTGAGGGAGAGGGAGAG
GGAGAGAGAGAGAGAGAGAGAGAACACCAAGAGCAGAAACAGGGTGAAGTTATAGTGGCTTCCCGTCAGGGGTGCATAGCAGCAAGGATTCATA
AGGGTGAATCAAGATGAGTCTAATACTTTGGAATGGAGTTGGAGTTGGGCATGCATAAGCCTACAATTTGAGAGACTGAGGCAGGAGGATCATC
AGGACTTTGAGGGTAACCTGGGTCTCATGAGGCCTTGTCTTAAAAAAACCCCAAACTAATTAGCCAAAGAAACTTCTTGCAGACGATGTGGCTT
AAGGCAGTGTCGGGACCATCCCTGTGGGTTCAGTCCAACAGAAGCTCTCTCTCTACCTTCTTGGATGTGTTATTTATCTCTCGAATTCTTGTTA
CTGAGACTTCACCAGTCATTGGACGTAGAGAACCATCAGATATTTTAAGGAGGTTTAGGTGGTCTAGGAACAGATTATGCAACATGCATCAGTT
AACTAGGATCTGATGCTAGAAGGAGCTCTAGGAAAGTGGACATTGCCGTCTGTCCCCTTATGGGGACTGAGAAGCTTCCCTTATATCATTCGGT
ATGCCCTTCCGTGGGCCAGGGACAGACTTCCTTAAAGGAGACGGGCTACCACAGTGTCCTGACCTCTAACACTCTATAGTGTGCTTTTTCTTGG
AACATGTATTATGTATTGCATATTTGCAAGGTGGCACTTTTGGCTTGGTTAGAAACAATTAAGGAGAGTTGAAAAATAGGCCATTCTTCCTTAG
CTTTCCCTGCCAAGCCTAGGGTTGGTTTTCTTTTTCCTTAAAAAGGAAACATTCATTTTCCTAAAATTAAAAAACAAAACAAAACAAAACAACA
ACAACAACAAAAAAAACAGAACAGGATGAATCAAAAGCTGTGAGGTTTCTCCACCAAGGGGTAATTCTGGTTCCTGTGAGGCGGGCAGACTGTG
ATGAAGTGACACTGACAGACTTATTTGGAATAAAGATGCCAGAGCGTTTTGACAGACTCATGGGTTTTGTAGATCTGTAATCTAAAGTGGCACT
ATATGAGGCATCTTAAGAGTCCCTACTCGGCATAGCATAGTTGTGTTCTCAAAAAGAAGAAAGAACTTCCATGCGGTTCTCTGTTCCCCATCAC
CTGATTTCTAGCCATGAGAAGGTTGCCTCCGTGATTTGGAAGTAGCTGCATAGAAAGGCTGGAAGGCTGAAGGGCTGCGTGGGTACTCTAGGGC

CTGCCACATGGCCGAGGCAGAGGGTTCACTCACACTATGTCTTAAATGCACTCTTCCTTCCTGGATTCAAGAAAAACACAGTTTTACTTAGTGA
TTGTGAGGCCATAGTCATGTGACTGCTGTCATAGTCATGTGATTGTGCGGACATAGTCATGTGACTGCTGTCATAGTCATGTGATTGTGAGGCC
ATAGTCATGTGACTGCTGTCATAGTCATGTGATTGTGCGGACATAGTCATGGGAGCTACTTAATGTGATTACTGATAGAGGACATAGACTCTCA
TATTCTGGGCTTGTATTTGCTCACGTGGCCCACACTGGTGCGACGTGATTGCTGTGCTCCCTGATGCCATGAAGTATATACCACGACTCTGCCC
TCTGACACGGCAGTCCAAAGCTGTCTCCAGTCCTGTTTGTTGAGCACTTCCGTTATTATACTAGTTCTCAGATAATGCCATTTTAGGGGACTGC
ATGGTTCTTGTTGTAATTTGAATTCCTGAGCCAATTCTGTGAGTAAATGGAGAGTTGTTCTTAGGTTTAGTGTTATAAATTTACTCTTTCTGTC
ATAAGACCAGAGAGTTTTTGGGAAATGTGAGGATGTTGTTGCCTTGCCACAGAAGGGCCATCCTTAAAATTGATTCCTTAAGTAGTTCATCTTC
AAGATAGTGGAGTGGCTCCTAGTTGCTTCAGAGAGTAGGCGGATAGCCACACCATCATCAGTGTGAGAGGAGAATGATAGTACTTTTTTGGATT
TTGTTTTGTTTTATTTTGTTTTTAAATACAGCGTGATATGAATAAGCTATACAGTTTAGGTCCTAAATCAAAAATTATAGTTGAGACAATGTTA
CTTTGCTTGTCCCTGAATCTTTTTGCATACTTCCTGTTCTCTGGGCAATAATCTTCCTACTCCTTATATTTGCTATAATTTGTGGAATCTAGTA
GTAGCTGTTGGGAGTCCAGGAAGAAGGCAGTTACCCATGTATGGCATATCGAGTGAATATCTAGAGGTGTTTTGTTTTTGTTTCATTTGATTGT
TTGTGAGCCTGATATGTAGACAAACACATTACTTTAAGTTGATTAGGATCCTGGAAGATGGCTTCCTGACTTGATAACCAGACCCTCCATAGAG
GTTCAGAATCCCCTGGAACTTCAGTTCTAAGGGGATCTGTGCCCTCATCCGACCTCCGTGGGCTCCGGAATGCATGCAGTGTTAAATAAGCTTA
CACGTACACACATATACACATACATTTAACAGAGTCTTTTGAGAACCTTGGAGCTTACGATAGGTACATTAGATCTCCGAGAAAAAAAATGATA
TTTTATCTAAATTCGTTTGATCTGCTTCCTTATTTTCACCAGCTCTGCCATTCTATTGGTGAGCACGCTCTTACCGTGATCACCTTTTATCAGT
AACAGGAAAAGCCTGGAGGGAGGCTGGGTTATAGACGTTATCCACTCCATCTGAATAACTGTTGTGAATGTTGGTAATAACCAGTCTTTCTTTT
CTGTGTCTTCAGGGTCGACATGTTAAAACGGGTCAGCTGGCGGCCATCAAGGTTATGGATGTCACCGAGGTAAGGTTGAATTGACCATACATGT
TTATGAAGAGATCGAGACTTTAAAGTTACTCCAGCCCAAGTACCTTTGCCCTTCTCGGCGTAGTTAGTGGTTGTGGTCACCTTAGTGCATGGCT **Exon 3**
TTCCTGCGGCGTTAAGGCTCCAGGTCCAGCAGCAGGCCCGTGGACGAGTTGTTGCAGATGGTCTCAAGAAACGAAGACTCTTGTTTGCTTAGTG
CACTTTTTATTTAAACAACTAACCCACAACAAACTCCCTCCATCTGAGTGATGTCATCCTTGGCCACAAGATGGGCTGTAATTGGAAACATCTT
CACTAATAGGAAAGAAAATGTTGGGGTGGGAGGGACCATGGGGGGTCACATGAACGGCGATTGAAGGGAATGCTCCCCTTAGAGTATTAGACCA
CATCTGTGCCATTCCTTTTATTTATTTATTTATTTATTTATTTATTTATTTATTTATATTGAGAAAAGGAAATATTGAGCCAAGTTCCTAGAGG
CTGAAAGACAGAAACATCTGTTTAGAAAGAACGTCAACTAGATTGTTGTTGGCACAACTTAGTGGCAAAAGATAGTTCTTTAAATAGTCAGTGA
AGATTTCCTGTGCAGCTTTAGATGGGAAAAGAGAACTGAGCTAAATAAAATGTGAGCCCATGCATACACGCACACTCACTCGCGCGCACACACA
CACACACACACACACACACACACACACACACACACACACACACACACTCGAGGGTGCCTCTGTGAGTGTATCCTTAATGGGTTGTGAGGAGCTA
GGGCTACCCCATAGTAACCTGTTTTAGTTTGTCTCATCCTCTGTACTCACTGCCTTCTATGCAGTGTTGAGTCAGATTCAGCGCAGTTACCGAG
TTCTAGAAAGAACTAAGCAGAAGTCTGCAGCGTCTTTACATCAGATCGTAATTTCCCTAAATCTGGGGCTTACTGAGGAAACTGATGGCTGCCG
ATGAGTCATCCATAATCTGGAGAGGTGTCCTCCACCATCCCTGCTCCTCTCACACAGCATGGGCCTCTCCAGTGCGCACATTTGTGAGTTAGAA
TTAGGAAACATTGTAGCCCACAGGTGACAGCTGTTGAATCCCGCAGACCCACTAGGGAGGATGTGGGGTTCTGTGGGGTGACTTCCCTCTTGTC
TGTCAACAAGGATAGCTAGAGACTTTTAAGAGGAAACCTGTTTGGTTGGTTATCCAAAAGATTCTTGTAACAAATTGTGCTGTGGGCAGGACAG
TTCTGTGGAAAAGTAGATGCTTTTCAGCTAAACGTTTCCATTTGTTTGTTTGTTTTTCAAGACAGGGTTATTCTTTGTAGCCCTGGCTGTACTG
GATTTCACTCTATAGACCAGGCTGGCCTCAAACTCAGAGATCTGCTAGCCTCTGCCTCCTGAGTGCTGGGATAAAGGTGCACGCTACCACTGC
CTGGCTCATTCAAAGATTTTTAAGGATCAGTGGAAGAGTGGTACATTTGAACTTACTGTAATTCAATGTAGTAGGTGTTTATGAAGTGCTCTGA
GCCTGGGCCACGCAGTGACAACTTCTCGATGAAGTCCCCATTTCTTCCCGCGTGACCCCTGTTGCTGTTTTGGGATATGGTGTCGCCAGAGCCA
CAGAACCTATAGACTGGGTTGGGTGGTCTATTGTTAGACTTAGGTACCACTTTAAATGGTCCAGACATGGATATATCCTTTATAGGAAGCAGAG
GCAAGCAGATCTCTCTGAGTTTGAGGGCAATGTATGTAGCAAACTCCAAGACAGCAAGGACCAGGCAAAGAGACCTTGTCTTTGTAAGCCTTAA
GCCTTGGGTGTTTATGGAAGCCAGAGTTTAGTTTCTTTCCGGACTTAGTGCTTTCAGCCCACGCTGTGTTTCCTGAGTAGCTGCTGTGGCCTCC
TGGTATGTTACTTCACACCTCAGATCCATTTCAAGTCTGGAGCCTCCTCTAAGGCCAAGTTTGATTATAATTGTGCACCACATGGTCACCAGAG
AAAGGAAGAGAAGCATTTAGGAGAGTGTAAAGAAAATCAAGTCCTTTCCTCTTCTGACAGTAGCCCTTTGGTGTATTTCTTATTCAGTCATGTC
TATATGAGAACATGTATGGGTTCATGCATACCTGTACATGTACTACATGCCTTTTGTATTATTGTTGCTATTTTCTGTGCTGGGGATAAATGAG
GCCCTTGTGTGTCCAAGCTAGATAAGTGCTGTACTGCTGAGCTACAAAAGGTGTTTTCCTGCTTGAAAAACAAGGTGGCAATGGTCAGGTGATG
GACACGAGTATTTGTAGTGCTCCTTTAAAAATAATTAATGGGGGGCTGGAGAGATGGCTCAGCGATCCTGAGTTCAAATTCCAGCAACTACATG
GTGTGATGGAATCTGACGCCCTCTTCTGGTGTGTCTGAAGACATTGACAATGCACTTATATATAAAATAAATAAATCTCTTAAAAAAGTAATTA
ATGGAAGCTCATGTATTCCAGTCTGTTAGCTCCTCAGTGATGTTTCTTACATGCTCTTCTGCTGTGGCCTGGGGCTCAGGAAGGATCCCGAGGA
AGATTGGGAATAGAAGGTTGTGAATAGAAGACCTTGGACCACTGGCAGCATGGAAAGCTTGCGCTTGCCTGTCCCAGCACTGAGGCTTCTGGGC
TCATGTTGCTGGGGCCTGGGTGTCAGATAAGAGAGGTTTGCTGCTAAGCAGCAGATGTCAGCTCTGGGCAGGGGGGAGGGGAGGTCATTGGAGA
GGGTCCTCTTGACAAGAGTGAGATTGTCTGCTTGCCATTTCGTCTCAGGAGCACAGAGGTACAGGAGCCAATTCCTGGGAAAGTCACGAGCATG
CGTCTTATTAGGAGGGACTAGGTGCTGGTCAGCAGGCTCAGCGGGTGCGGGAACAGTGGGTGGGAAGACCACGGCATGGCGCTCTTGTTGGCAG
CTCCAGCTGCAGTAAGTGGGGATGTAGGCCTGCCCCACCATTGTGTTTGGTTATGAGAAGAAGCCGGACATGACACTACAGATGGGGCTGAGTA
GTCTGCAAGGGAGGCATTTTTTTATAGATGTGCAGATCTCTTCCTATTTGCTTTGATCTTGACAGAATTAAGAATTGAGGTCAGAAGGGCTCTT
CTGAAGGAAACTGTCTTTTATCTGGTTGGCTTTGGAAAGGGTTTACAGAGCATATTACAGATTTATTTATTACAGATCTTTGTTAACGATTTGA
TATTAGTGCAGCAAGTGGGTAGTGTTATATGTGTTTTGTTTACAGTAACTTCAGGCTGAGAGGAGCTTAGGGAAGGAAATGTCTAGAGAAGACT
CATGCACTGCTCACCCAGTCTTCCCCAGCGATAGCATCCTGCAAAGCACAGCCTCATAACTCAAGTGTACAGGATGCAACTGACTGGACTATAT
AAGGTATAGAACTTCAGCTTCGGAGCCATGGCACAGGCGAGATAGTTAGAACAAGTATGCTGTGTAACTTAGGACCGAGGCAGGCCACTAGAAA
CCAACTAAACACAAACCTGTCCAGGCACTTGTAGTGTTGAAAAGAGTATTTGATGTTTAACCAGGGCTTGTTACTGCTGAACTGGTATGCCCCA
GCTCTTGTCATTTAAATCCTTTTAAAAAAAGATTTATTTATTTTATATATGTGAGTACACTGTTGCTCTCTTCAACACCAGAAGAGGGCATCAG
AACCCATTTCAGATGGTTGTGAGCCACCATGTGGTTGCTAGGAATTGAACTCAGGACCTCTAGAAGAGCACTGAGCTATCTCTCCAGCTCCCGT
CATTTAAATTCTTAACAGTTTCATTTGTATTTACATAAGGGCGCATAGATGTGTATAGCATAAGTATATTCATTCTTACAGAAGAGGGGCAGTG
TCTGTAGGTCATAAGCCTTAGAGAACTGTGGGTGGCCGGCTGCTTTTCCCTATTCTGTGCTGTTTTAGGCAGCAAAACTGACACCCAGCCATTC
TACAACAGAAGGCACAGGTCTTCCGTGTCCTACTTCAGCTCAAGGTGTGGTACTCTGTCTGTATAGGGAAGATCAGCTGTTGAGTGTACACAGG
GTGTGGCCTGGATTCAGTACAGCTTTGCCTTGTTGATAGATTAGTTCCTGTAGGCTCAGGTATGACTGTGATGCTGCTGCCTGTGGCTTGTGTG
GGTTGTTTGTTTCCCCCCTTCCTCCTTCTAGATGCAGTGTGAACATGGAAACTGAAAATGGTCAGTGGGCAGGGGAGGGAAAGTGGCATAATCT
GATCATTCTTCTCAGCCTGGCTGGAGGAGGGTGAGCCAGGCAGATTTCCATGTGATAATAGCCTTTCTGCTTAGGTAATGATCTTTCGGGTCTT
CATGGAGTAGTCCTTAAAAGTTTGTCTCTATAAACTAGTATGGACTATTGGAAAGGAAATATCATTTGAGAGTCTGTCTGTCTGCCTACCTGCC
TGTGTCTCTCCCCACCTCTGTGTGTGTGTGTGTGTGTGTGTGTGTGTGTGTGTATGTGTGTAGGTGCCCTAAAGGGCTGAAGAAGGTGTCAGAT
TCTTCACAAGTGCATAATGCTGGTGGACTTAATTCTCCTTCATGCAGAAGCCCAGCTCTGCTCTCTCACTACTTTGAATGTCTGAGTGAGTGTA
GGCATGGGATGGTAGGTGGTCCTCTCTCTGACAAGTGTGTTCTGTTTCTTGTCTCCGGGGAGAGTTCCACTTTAAAAACAATTGTTCTGCCTTG
GCTAAACCCAGGAGGCTTCAGACAAACTCTCACCAAGTGTTTTGCTTTATGGTCATACAGAGTGACAAGAGATGAGGGCCTCTAAGGAGGGTGA
CTGTTGTTAACTCAGTAGTGGGTGGCATTGCCTGTCTGCTGACCGATGAGAGTGGCTGTAGTGTTCTGGGGGCCTTTGCCCAGACCTCTCTCCT
AGAAAGTTCCTCTGCCTCCCTGTCATCTATCTGCTTACTGTTAGTGCACCCATGACTCTAAGAGAAGGGGAGGGGGAGAGGGTAGGTGTAGGAG
TGGGGGTGAGCACTCAAGGGACTTGAGAACCCAGTGTCAAGTTAGTGAGCCATGGCTAGACCAAAGTGAGATAAGTGGTTGGTGGGCTGCCCTG
GAGTCTGTCTTATGAGTCATATTTGAGCAGGATTTTTATTATTTAGTTTCTAATTACCTAAAGCAGAACTCTTTCTTTTACTTTAAATAATTGG
TCTGCAGCTTTCTGAGTGGCTAAAGCAGTAGTAGGCTTAAGGTAGCAAAACCTCAATTGTCAAAACCTCACAAGTTTTGTTTGCCTTTTTGAAT
TGGATCATGCTAATCCTGTAAAAGTATATTTTTTTGTCCAGCAAAATCTTATCTTCAATTCTCTTATACAGCTTTAAAAAACTCATTTGAAAGA
ACAACTGTTCTGATAAGCTATACACAGTGCTCAGTGTCAGGGGATATGCAGTGTGTTCAGAGATACCAGCGTACATTCAGACTTTGGCAGCAGG
AGAGCCGTGGCAGAGGGCCCGCCTGGGTGACACTGTTGGGTAGGACCAGCAGCATCCATCCATGGCTACGTGAATCAGTTATCTTTCCTTTTTA
CCAGTTGTTTGGGAATCTGTAGGTTATTAGAGTCATCCTTCCCGGGTTTTGGGGGTAGGAACGCCTGCAGGTTTGTCTGTCGTTGTGTGAATGG
AGGTGATCGCACATCTAGTTAGTACTCAGACTGTCGTTCATGGTGGGAGAGTTAGCTGGTTACACTGGCGGTGACTGCCCTGGCCTGCTGTCCT
TGCTCCACCCACTACTGTTAGCAGGAACTCAATGCCTGGATTGTGTACATTCACCAGGGCCTGTTTCATGTTAGACTCTATAGGAATACTCTAT
AAAGCCAAAGATAATTGTAGTTCATTTTCTTAGAACCACAGCACATATTTTCTGTGTGCTATGTGCTGCAAAAAGATTTTCATGCTAACCTAAT

ATATTATTTACCATTGTGGGTGGCCTCACCTGCTTGGAAGGGATTTTCCATCTTGGATGTAAAGAAACTGAGTATAAGGGATTGATTCATGTTA
AATTTAAAGCAAAAGCTTCATCATCCTTGTATGCAATGGGGTGTGTGTGTGTGTGTGTGTGTGTGTGTGTGTGTGTGTGTGTAAAACAAAGAAA
AAGAGGCCATGACTTTGAAAGGAAGCAAAGTGAAGGGTGAGGTATGTGGGAGGATCTTAGGGAAGAATGAGAAGGGAGAAATGATGTAATTATA
TTTTAATTAAAAATATAATTTAATTAAATCGGTTGCTCACTAATACTTGTAAAGTCAGAATTAGAAAATTCTTCTAAATTAGAAGAATGAATTT
TACCTTACTGGTTTCACAATTATTTTATACTTTTATAATATTCTTGAATTTGGGGGGCATCCAGTTAGTTACCATAATTATTGGTGGTTGGATC
ACCAACTAGCTGAGGAGTGATCTTAATCCATTGTTGAGACCATGGCCTTTAAAGTCTGCGTCTGAGCTCCTGTGCACCTGAGGCCGTGGGCTGG
CGGTAAGAGGTGTGCTTGCTCACACACAGGTACTCCTCCACATGCCACGCCCTCCGAGCAGCCCTTGGCCAATCCTCACTTGATCTGTGTGAGT
AACACTGTTCAGTGTGTAGGGCACTTCTCTTCTTTCTGTGTAGTGTGCAGAGCTCATGGAATTTGCCACAGGCATGGTCCAGTAAGGAGCCTCA
GGCAGTCAAGTAGAGTGTGTGAAGGCAGTTGCTGGGCGAGCCCCTTCCTTCCTCCCTCCCTCCCTCCTTCCCTCCCTCCCTTCCTTCCTTCTTC
TCAGGAATGACTTACATAAGGAAGTTGCCGATCTTAGCTCAGTGTTTTGCATTGGTACTGACTGCAGACAAGCCTGAGGCCTGTAGTGTTTCTG
GCCTAGCCTAGCAAGGCTGTGTGCTTCTTCAGGTTTGACAGTGACCCTTCAGCATCAGGGATGGGAGACAGGGAGGCAGACCATGAGAGCTGAC
AGAAATCTTATGCATGACCATGTGGAAAGTTAGAAGATTAAAGAAGAAATTACCCTAGAGGTAGTCAATGCCAGAATTTCTTCCTCTTCCTTGT
TTCCTCTCTTCTGTCCATTTAACCCTTTTAATAGGTACCTGACACATGCACACTTATACATGTATGGTTACATTCTGACAGATACCTAACATGT
GTACACTCACAAGTTCAACCACATTTTGATAGGTACCTAACACGTGCACGCTTACACATGTATGACCACATTCTGTTGCCAAGGTCATACTGCT
TCGATAACTTCTGGGTTTTAGATTTTAGATTCTTAGGCTTTATAAAACGTTAAAGTGTAATCACTTTTGTAGAGAACTTGGTTTTGGAAATAAA
CATAGAGTATTTGAGGGTGTATGTCATTGAAAACACACTTAGGACATTTTGCCTTAAACATTGGGATCTACCAGGATTTCTGGGTGTTGACATG
AAACTTTGGAACAACGTGTGTGTTACTGGGCTTCAGGAGGATCAGCATTTGCCGTTTGTTGTTGTGTTTCTTGGAGGGCTTTATCACAGAGTTG
TGCTTTGACTTCTGTGACACCATGGTCTCCTTCAGGGGAGCATAGTCCTCCACACTCATTCATAAGTGAGTGGCGTGTAGGAGGGGAGAAGGCT
CTGCCCGCTCTGCTGTTATTGTAGTTGCCGTCTCTTGTCTGTCCTGGGCTCCAGGTGAGCAATAAGGTCTGACTGAATGACTCAGCAAGGAGGG
AAGAAGGAAGGCTCTCTGAGGGCTCTGACTGACTAGATCTGCCATCAGGCAGAGAACAGCTTTGAGGCCGTGGCCATACCCAGAAGAAAGGCAT
TGATTCAGTTAAGTAAAGAAGAACATGAATTTAATATTGACATTGGTGTGTTTGAGGGAGGGAGCACTTTTCTTTCTCCATCACTTGGGCATTG
CCAGTTTGGAAGAGGATTGCGGCTTTTCCCCAAAGCACTGACTGATTTGGATGAGGATGTTACTTTGAAGGCTGTCATTCTTGTGGCCTGATCC
CATAACAAATCCCATTTATTCTACGTTGTATCCTCTTCTAGAATCTGTAAAATTGTCATTTACATTTTGTTGAATGGTGAATCGAAGTCATGTA
ACTCTTTTATGTTTGAATCAAAGCCAAATTAAGCTTAATCGATTCTGTGCTACCTTTAAGATGGCTGGTGCTGAGTATCCCACTCTCGGATCTG
CCTGGACCCTGCAAGGTGAAATAGTTTGTTTTCTTTCAGGGACACGGCTCAGTTGTAGATCTTGGGGCAGAACTCTGCCAGAGACTTTGGCTCA
CATCACAGCTCCCTTCTCCAGCTTCACAGAGACCATCAGTTGCCCTCCCGCCATTTGCGCAGCTGTCACTGACTGATGATTAATTTTGTAAAGA
AGCCAGGTGGAAAGAGGATGAACTCAGAACATTCCTCAGTCTTCTTGGGTCCCAGTCATTGTCTCTAAGGTGTCTCTGTCCCTCAGGCAGTACA
GTCATCAGTGGAGTGAGAGAGACAGAGAGACAGAGACAGAGACAGAGAACTTAGATAATAAGGGATGGAAGCCACATTTTAAGGGAACCCAAAA
GGGATGGAGACATTAGCTCAGTGGTAGAGCACTTGGCTAGTACACTAAAGGCCTTGCAAAACAAACACGCTTACTTCAGACACCTAGGGGGACG
TTAAAACCAGACAGATACCTACTAGGCACACTCCTGTGTGGTGTGCTCAGACAGTGCGATGGGCAGTATGTTTCATTTGATTCGTTTACAGCCC
ATAGCTGCTATGTAAATTAATGCCATGCAAATGTTGGCTTCTCCCAGCCCCCAATCCTCTGATAGCCAGGGAGCGAACTGAAAGCTTGTCCACT
GTCAGAACCCAGCTCCAGACAAAGAACTTGTCTGTGAGAGTTGCTCCTGCTGGGCGGAGGCCAAAATGCTAATTGGCACCAGCTGCATTAACTC
ACGAAGTGGGGCTCAGCAGCTGCCTCCCCATTGAAGGGAGCCAGAGGACAGCCCCTTTGCTCCTAGAGCTCCCGCCCCATCCTTCAGATCCCCA
TGTGTGATCACCTTGGCTCCCCTGAGGTGGAGTATGAAGGACACAGTAGTTTGGACTTACAGTTAGACACATGGGGTGGCATATAAGCCTCCTC
TGCTCTTTGTCAGTTCGACCGTTGGCTTACATTTCAGCCCTTCGTCTGCTCACCTGATTTGTCTTCCACTTGAACCAGTTGTTTACTTTTGTGG
TGGTATGTGTGTGTGTGCATGCGTGTGTGTCTATTAAGTGTTGAAATAAGACCACCAAACTTATTATTTTTTTTAGAAAAAGATGTGTGTCCTC
CCTACTGATGCTGAGTATAGAACTAGGGTTCTCTGGCTGTTTCTTTTAACTCCTGAGCCATCTCTCCAATCCCCAAACTCTAGTCATCAAAGGG
GATTTGGAAATCATCCTAACTACATTCTAAAGACAATATAAAACACTGTATACTAAGTCTTCATCGTAAGATTCTAGTTGTAATTGACTCAAAG
GTCCTTGGCAAATTCTATATTTTCTGATGCTTTTTTTCCCTCTGAGGCTAGGCCTGCATAATTAGACAGTAAATGACAGTGAATGACACTCAGC
ATCTTTCTATTTTATTACATGATTTTATTTTAAAATCATGAAAACAACAGAAGTGATGTTCAGAGTGTCTGTTACATTACATCATCCTACAGTA
GTGCTTTGGGATGGAGACCGCGATTTCCCATCAGGACCTTTTCAGTTGAGTTTCTTGCGCCTTAGAACTTGGTTGCTGAGGACTGTAGGACAGA
TTTTGGCAAGCAAATGAGTGCCATCACTGACTCTCGATGATGTTTCAGTCATGTGGAGAGGCTACTGTTGACTTTGATTGTGCTTAAGGTCTTG
TGCTTCACAGACTCTAGGATGTAACCCGGAAGAGTCCCATTGTACAGTCTGCAAGGTCTTCTCCACCACATCAAGTTTGTAGCCCAGTGTCAAA
GGTGTTACAGCGTGAGTCTTGGGATGGTGCAGCGTGATGGCAGAGCACAGTGTTTCTCTGTCCAAAGATGCTTAGGATCACCAGAAATGGACCA
CGAAAATCTGCCGATCATATTTCTCAGTATACATACTCAATGTTTTCAGACCAGCGGACAGCACTGTTTAAGCTTTTAACAGTTTGAGACTATA
TATACACTTGGTCTTTTATTTAGTCCGTGGGTTATGGAACCTTTGGAAGCTTTGACTTCTTAAAGGGACAAACAGCTGGGTCTTAAAAGAATGC
CTTCGTCCCTGGAGGAAGAGAACACTGCAGCCTGTCAATTGTTGATCTTAATTGCTGCCCAGGGAGCAGCTCCCTGGAGGCTCTTACAGCCCTA
CTCCCTGCTAAGAAGTGGACCCCTCTGTCTAGTGGGTGGAACAGTGGTCCTGGGTTAACGGAATGAGATGGAGAGTGGGAGGGAGGGAGGGACA
CATGGGTAAAAATGTTTTCAAGTCTGGGCAGCTCATAAAGCACCGAACTCAAGGATTAGACTGATTGGACTTTAAGACAGAGTTAATATTTGAA
CTTTGTCCTACCTTGTTTTTTTTTTTTAAACATTTTTTTTAATCTTTTTATTTTATTTTATGAACTTTGTCCTACCTTGTTTTTTTTTTTTTAAA
CATTTTTTTTAATCTTTTTATTTTATTTTATGTATATGAATACACTGTAGCTGTTTTCAGACACACCAGAAGAGGGCATCGGATCCCATTTCAG
ATGGTTGTGAGCCACCATGTGGTTGCTGGGAATTGAACTCAGGACCTCTGGAAGAGCAGTGAGTGCTCTTAACCACTGAGCCATCTCTCCAGAC
CCATGTATTCTTTTTAAAGACGATCATACCGCGTGTGTATCCTGGGCCAGCCTGGAACTCTGAGTGAGAAGAAGCTGGCCCCAAACTCAGAGGT
CCCCTTGTCTCTGCTTCCTCAGTGCTTGAGTTAAAGGCAGACACTATCACCCCTGGCTGTGCTCTTTCTTCCCAATCTCTGCATGCCTGCTACT
GTGAGTGTACAGCAAGAGGACGTCTGGCTTTCTGGGGCCTGTGTTTAGCTGTGGGCCTTGTTCCGGGAGCAGCCTCGGGTGGCTTTGTAAACAG
GGCCCTTTGTTCCTCACCAACAAACTCACAGCCTTCTCTTGTCTCACCTGTGTTATCTACATCATCCTGTATGTATGAGTGTGTGTATACAAAG
GACATGATTAAGGCCTTTCTAAGAGAGGTTGACTCTTCTTAGTTATGTCAGGGACCGTGCTTCTTTCCGCTTCTCTTCATCAGCACTCCTTTCT
GTTTAGTATGAAGTTCCTTTGGAGAGAAAATTCAAATCGAGGCAGTTTTTATATTGGCCAAGTTAAAAGCTGGTTTTTGGGCAAGCGTGTGCAC
ACACCTATATATTACTGAGAAACCCCAAATCTCCCAGGAAAGGGAGAAATGCCAATAAAGAATGAGTGTGAAACCCATTCTATGCAGAAACCCA
AGGGGCTTGCAGCAATGAAGTGAGTGTATAAGGAATTCTAGAGATGTGCTCAGGTGTATTCACTGACAACATGCACTGTGCTTTCCTACCCCAA
GGCTGATTGATTCCTACACACACACACACACATTATTCTCTCTCTCTCTCTCTCTCTCTCTCTCTCTCTCTCTCACTCTCTCTCTCTCTCTGTT
CTCACTTTAATTCTGATTTTTTTCTTCATTTGGACGGTACTTGGAGATCTTCCTTAGCCCGCCCTCTTACTGGAGTTTGTCAGCGTGTGCAGCA
GGTGACAGGATAAAATGCTCATGCCGATGGTTGTAGTTCCTTCCCCACGTGTATAGGAGAAACAGACTGCGGTTCCTCATCCCTGGTCATTCAC
ACGATGATGGTTCCTGCCTGTATGTCCCTCTCTTTCCTCCCTCTTCCTCTTCCCTTCTTATCACAAGGGCTCTCCCTCCCCCCACCCCTCACCT
GCTGCTCTCACCTGAGTGGGAACCTTGCACACTGTCCCTGAAGTCATGTGTTGTCGTCTGTCCAGTACCTTGCAAAACTTTTCTTGTCTTAAAT
ATATTCATAAATGTTTCCTAAGGAAGAAATGGCTTTTAAAAAAAGATCATAGTATCAGTCAGATTACCTTTTTATGTGTGGTGTTTGACCTAAG
CAAATTGCCACTCAAAGAAGTGGGTAGATGGCCTGAGGGTCATTGGTAGAGGGGTGTCTGGGATTCTGCCCTGTCAGTCCTGCTGATTATCATA **Exon 4**
CTTGTGGTCATATTCAGAAGAGCAAGCTGTGGATGAACAGAGTTGTGCACTCTGTCCATTTCACTGGTGTGGATGGGAGGAGATCAAGTCGGCC
AAGAGCCACATGTTTTGTTATATTTCCCCTAAGGATGAAGAGGAAGAAATCAAACTGGAGATAAATATGCTGAAGAAATATTCTCATCATAGA
AATATTGCAACATACTATGGTGCTTTCATTAAGAAGAGCCCTCCAGGACACGATGACCAGCTCTGGGTAGGTAGATACTTGCAAGCCTTTGGCG
TGTGTCCCATCTGTGGTTGTTATAGTAAGGGGATTGAGTGCTTCGCAGGGAGCGGGAAGGAAGGAACAAAGGACAGGGGCTTCCTTATCCTGTG
TCTGCACTGTGGCAGAGAGATCAGGAATGGGCAGCCATCTTACAGGAAGTTCTTCGGTGAGAGCCAAATCAATTTCCCTTTCTGCTTCCCACCA
AGGCATTGCCAGTCATTCATTAGTAACGTTAACGATAGTAGTTTATTATGAATAGTAAATTCCATGGAAATTTCTTGAACTGACACACTTAGCC
TTTAAAAACAAAAGCAAGGGGTTGGGGATTTAGCTCAATAAGGCCCTGGGTTCGGTCCTCAGTTCCGAGAGGGAAAAAAAAAAAACAAAACTAT
TTCTATTGAAAACACTGGCTGAGAGCCTGTCACGTGTGTGAGGGAAGCGGAAGGTGATGAGTTTATAAGGCAAAAAGGTAGATGGATAACTGAC
TTTAAGAAGGTTAGTGTAGGAAGCGTGCTAACATGCAGCGGTACTGAAGGGCCAGCGATAGATACACCCAGAACCTGCCACGCAGTTTCTTGCT
ACTGATAGGTGACAAAGATGTTGCCGGAAACTCACAGTGGAGAACAGTGTCTGTGCCGGACAACTCCAGGGAGACTCAGGAATGGAGCTCGGTT
GCTGTCTCTCGTAATGCACAAAGATAATTGAAAATAGATTAGAGGTCTTAATGTGAAATCTAAAGCTCTGAATCTGTTAGAGGACAGCATAGGG
CAAGCACTTCAGTACAGCATTGGGCAAGCACTTCAGTACAGCATTGGGCAAGCACTTCAGTACAGCATAGGCAGGGCTTTCTGGAAAGGACGCC
AGAGTTCAGGCAGTAATAGCAACAGTTGGCACATGGGATTGGATACAATTAAAAGGTTCTGCACAACAGAGCAAACAGGAAGAAGCTATTTTAA

AGGACAGGAGAAAACTTTTTCTGTCTGTACATCTGACAGGATTAATATCTAGAATATACTTTAAAGAACTAAATAAACCAAACAGCTCAATCAT
AGGTGGGGCACTGAGCAGGCAGTCCTCAGTGGAAGCACGTGTCACCAGCAAATGCCAGTGAACACGTGCAGCCTCCTTAGCTCTGGAGGAGAAG
CAGGCACTGTGAGGATGTGAGCACAGAACTCACACGTGTGAAATGTAAATGGTCCAGCCACCGTGAAAGTTCCTTTTAAAAAAAACACCCTAAA
ACTAGAGATACTGTAAGATCCAGCTATGCCACATTAATTACCCAGAGGACCCCAGTGCGATATGTCAGAGGCCATTACCTGCACACATGGCCAC
ATTATGCAGCCACACTGCATACCTGTGGGCAGATACATAGAAAATGCAGTACGTACGTACATATGTACGTATGTACACACACACACTCTCTCTC
ACTGGAATTCTGTTCAGCTGCAGAGAAGTTAAAACTGCATTTGCTAAAACAAAGATGTGACTGAGATCATCCTGTTCTGTGAGAAAAGTCAGAT
TCGGGAAGACAAATATTGTTAGGGTTTTTGTTTTGTTTTGTTTTGCATTTAGAGTTCCTTAATGGTACATAAAATTACACACACAGACTGTCTA
AAAATAAAGGCGGGGAGTGGAAAGTTCCTGAGGAAGGAGACCTGAGGCCATCCTGTTTCACACACACACACACACACACACACACACACACACA
CACACACGTGTATACCTGTGCACTGTCAAATAAGTAAAGATACAGATGGCAGTGTGCTTGGGTAGTCTTGGCACACACAGACTGTTAGTTTAGT
TCCTGTTACTGTGTTAAAGCACTGACAGAAGCAGCTGAGGGGCGAAGGGTTTATTCTGGCTTACACGACAGAGTCCCTCAGATGGGAGTCATGG
CCGCCAGGAGCGTGAGGCCAGTTCCTCACATAGCAGTCCTGAAACAGAGCAGACAAGAAGTGCCAGTGCCCACCAGCGCCTGCCATCAGTGACT
TGCTTCCTCTGTCAAGACTCCCTTTCCTACATGTCACATGACCTTGTTACAGGCGCCAGCAGCTGAGGACCAAGTGCTCAATCACAGGAGCCTG
TGGGCGTCTCTCATTCAAGCAAAATGTACAGACACACTAGAGAGAGGGAAAGAGGAGGGAAGGGAGGAGCACAGGGGCTGCAGAGGGAAGGACA
GGAAGATAGCAGTGAGACAGCGATTCCTATCTTTAGTGCTTAACTCCAGTCATAGGTGAAGTTTTGGGACAGTTCAGCTCCTTTTCTAGGAAGT
CCACAGGTGTCGTTATCATAGTTAGATGTTACAGGTACACAAATTTCAAAACTGTAAGGGTTTAAAAGTCTCATTTCCCTTTTGATTTAGACTC
AGCTAATAGGAAATACTATGAAATGATTTCAAGACCATAGTGTATAAAAACAGCACATCTTAGGGAAGACCTTCTGGAGTACAGATTTCTAATA
ACCCAGTAGTGATGACCACCAAAGTGATTTTCCTTGGAGGAAGGGAAGAAGCACACCACAATTGCCACCCAGGCACGTGCCACAGAACGGGACA **Exon 5**
GCAAACAGTTGTTACTTGGAGGCTTCCAGCACGATCTCGCATGTGCCCGGGTGGTTTGTAGGTGACGGGGCTGTGTGATGTAGCTCGGGTGTGT
GCTCATCCTGGGATGTGGTTGTTACGACATGTCTGGTCTTCCTCGTAGCTCGTTATGGAGTTTTGCGGGGCTGGTTCCATCACAGACCTTGTGA
AGAACACCAAAGGGAACACGCTCAAAGAAGACTGGATTGCTTACATCTCCCGGGAAATCCTCAGGGTAAGGAGAGCGGGAGCAACCCTGTGCCA
TGCTGTGATCTCCTCTTCCACTTGGAGCAGAGTCCTCAGCTCGTCTTTGCCGTTGCTCTCCCATTAGAGATAGCCGTCTCTCGATAGCCAAGCA
TTCTCTGTACGATGTTGGTTTACTCTACATCGCGGTTGGATACGTTCAACTCTGAAGACCAAGATGTTGGCCTGGGTGGTTTGGAAACCATTAG
AATATACAGTCACCCCCTTGTTGTCACTTCCTAAGGAGTGAGCTTTAGCAGCCATACTGTCTTAGAGCTATGAACAAACATGGCCTCACGAGGG
AGGCCCTGTGGAGCTCTTGTGCAGACACTGCATCGATTCAGCATGAGGGACTTGAGCACAATCATGTCTGCTCCATGTGGAAGAGGTTGGGACA
CTCCAGTCACCAGTCCCTTGGGGCAGCGGAGGTCCTGAGTCTGTGGCTGTTAGACGGTGCACCTCACAGCTGAGCGAGCCTGCACCTCCTGTGG
TGTCGGGGGTAGCCCTGCAGAGCAGCACTCACAGTTCTGAGTGCTGGTTCCAGACCTTCAGTGGCTTTGGCACTGGCTCTTAGAATATCCTGGA
ATATTTATGAAGTCCTAATGCTTAGCGTTCTTCACAGCCGATTCCAGAGCTGGCTAATAAGCTCAGAGCAAGTTTCTAAACTGCCGAGTTTTTA
GCCACTAAGTGCATTCTTTCAAGTCAGAAGGCCGCCCTGACGGCATAGGAGGTTTTCAGAGCCTCCTATTTGGAAATTGTGTGAGTTGCTAAGG
GACTTCTTTCTTCCACTACCACAGCTGTTCTGTCCCTTTCCCGCAGACTGCATACTTAAACTGGTTGCCTAGTGACTCATCCCTCTATGTTTTA
GAAATAAAGACTCTGGATTCAAACTGAAGTTTTATTTCTTGTTTGCCTACTAATCAAAGTCTGGTAACGAGTTAGTCTTTTTTTTTTTTTTTATT
ACCGTAACTACATTTACTTTTTATATTTAAGATTCATAGAAGACTGCAAGTTTGACAACTGTCAACTAGAATTCCCAGAGAAGTGTTTTAATGG
CTCTCTGTTTTATATATTTTATGGAGCCTTCAAGAAGGGTTCCTTCAAAAGACTGTGTAAGGTAGGGAGTCTGATGGTAGGAGAACCTGTAATC
CCTCACACACATCCTCATTGAGTATCAGGTGCCTGCCTTTTATGGGTCTTACGGAGAGTTGAGCCTGACAGGGAGAAGCTGCTGTTGTAAGGGC
CCGGTGATGACAGACAGGGTTCAGCAGCAAAGAGGCTGGATCCTAGAGCTGGCTAGGCATGGGTTTGAGGAGCTTGGCTGATGGCTGTTCTCTG
TTTGAAAGTAGACGTCCCTTTTTGTAGGTAGTAGCTTTAGACTTGTGGGCTTTTGCTTTGGCTTCTCTGGTGCTGCAGTTTCAGGGACAGAGCA
CCACTCCGAGTAGATAAGGAGTCTGTGCCTTGAGCATAATTTCTAGACATAGGATGTATCTTGATATCAAAGTTGTTTGCCTGTTTCTATAAA
CCCAGGGAGAAGAGGTTATTGGTCGAGTGTAATGTGTTTAGTTTAGCATTTTGTCACTATCTGAGTTCAGGAACATTGAAGAGAAGCCTAAGTT
GCTTGCAGCAAGAGTCAGTGTGGTGGGAAATGTCATCCTTTTGAAGCACTGCAGATTGTTTCCTTTAGTAAGAACCCTCTCCGCTCATGCGCTG
TCTATAGTACAACTTTTCTGTGGTGGAGTTTTAGTTCAAAACAAAGAAACACCCAGATTCCTTGCTGGTGTTGTTTGGGGAGTTGCTATGTGTT
AGTGTTGATAGAGAAAGCAAGTGAACCCCGTGAGTCTTATAGGTCCTTGGACATCGTGGGGGTGGCTGGTTCCCATATGGAAAAGCCCCCTCTT
GTCCCTCCAGGCAAGGGTCCTATACATAGACCACCACTGCCTCAAAGTCCTCTTGGACTAGCCTACTGAACAGTGAGAGGCACAAGGCTCTCCG
GCAGAGCCTCTCCGTCCTTCCTGCCCGTTTCCTCATGCACATTCGAAGCCAGTTCTCTCCTTGTGGTAGAAAGCACTGTTTCCGACCGAAGATG
AGAACTCAGGACTTTACGTCACCAGTGTTGAGGGGCAGTTTGACAGCTCTTTTGTCACATGAAGTCACGGAAGATGAGCTGAGAAGGAAAACCC
TTTCCAGTTGATTTAGAATAGGCTGTTTCCTGAGTTGATGTGGACTTGGCATCTATGCCCAGGAGGAGTAATTATGGCCTTAGTTCAGGGTCAG
ACCCCAAGGAATTTAACTGCACATAAAGGAAATGTATTCAGTTTTGCCTATTTGTCTGATCTAAGGTAGCTAGCTGTGTTATGAACTATTTCCT
GAGTAGAAGCAGCAATCCTTACCCAGTATGGACTAAGAAATGGGGAAGATTTCACGCACTTACTGCTGGAAGGGTATAGATTGCACATAGCCAC
AACCGTCTGAGAAGACAGGGCTGCTCATGTTTGTCGGAAGATGCCTGAGGGTAAAGCAGAAGTTCATGCAAGTTAGAAAACAAAAGTGAACCGA
AGCGGTAGAGGGAGTCTGTAAGAACCAAGGGGCTTCAGTTTGCTCTTTAGCGTTTATGTTCCAGAGTGCTGAGGAGAGGGGCTTCCCAGTCAGG
GGAAGCAGTGCCGTCTCCGAGGGCTTCTCCCCACCACAGCCTTATGGTATAATGAGGTACCCAGTAAGATGAGTAATAGTAGATAGATATCCCG
GAGTCTATTTCACTGACAGATCAGTAAGTGAATTTAGAACCCACTCTGCTTGAAAAAGAAGTGTTCTGGAGCTGTCGTTGATAATTAATTCATC
AATGCTCTTCTTGATAGCACAGGCCCGTGAGCCCAGTTTACGCCGCAGCTGGTGAGTCATCCAGTGGTGCATTACTGCATTTATGTCACAGGTG
GATGTGTATGTTTTCCCACCACCTCTTGGGAGATGCTGCTGGACTCATTCTGGATACAGGGCATCAAAAGCACATGTGTCAGGAGTGTTTGTTT
GCCTAAGGGGAGAAGGATATTTTATTCCTACACAAGGCCATTCTGGTGACAGAACCATTTTACACTTGAACAGATATCCAGTGAGATACATCAG
CACTTAGCAGCTTGTTCCTCTGTAGTGGGAAGACAAGCCTACAGTTTTTAAACAGCCAGCTTTTAAGGCCATCTCTAATTCTTTTCTGTTCCCC
CCCAGGATGAGTCTAGCTCAAATAAGTTCACAGGGTTCCCCATTGAAAGATAGACATTTGGTGTGGGAAAGGAGCCATCCTGAACACAAATAGC
ACAAATTATAGCAGAAAACCCACAGATAGCAATCTAAAGCCAGTGTCATAAGCACAGCTTAAAGAGGTTGTGATCATCCCAGAGGGAAAGCCCG
GCCCACCGCGTTCTGTATGTGTGGCAAGGCTGACGGGGCCTTCCTGGGAATGCGCCAAGGGTAAATTTTACGTTCTAAATGTGGATCTACAAAA
ACTTGGCCCACGTGTGTACCTTATTATGAAAGGGTGCCCCTGGCCGTCTGTTTACAGGGACAGAGAACTGAGACTCAGATCTGCTTTTCTTTTC
AGGGGTTGGCCCATCTCCACATTCACCATGTCATTCACCGAGACATCAAGGGGCAGAACGTGCTGCTGACTGAGAACGCGGAGGTGAAACTTGG **Exon 6**
TGTGTGCACCATCCTGGTACCAGCGGGCAGCAGGCTGTACCCCTTTAACATTTAGCCAAGAGTTCATCTTGTTAACACTGTTTGCTTTTTCTTC
TTCTCCCCTGAAGGATAAGCGGACAGGCTTAGTGCTTAGCCAGCCCCAGCCTCCTCTCATCCCAGGCTTCTGTAGTGTCCAGATATAGCAGCCA
AAGGGAGTTTTAAAAGTTTTTGTTTTAACTTGGTGTGAGGGGATGGGGGGCACTGATAAGGCCCAAGCCCAGGAACTCATGACTGCCCTACACT
GACTTAAAAGTCATACTTTGTTATGTAAGCACAGTGGTGCCTGGCACAGTGCATTACTAGGACATGGGAGGCTAAAAAAATTGTGAGTCTGAAG
CCAGCCTAGGGTATGAAGTTGGGGGAGGTGGAGGAGTAGAGGAGGAGGAGGGAAGAGCGAGAGAGATGGGGGGGGGGCAGACACACAGAGAGAC
AGGCACACAGAGAGACAGGCACACAGAGAGACAGACACACAGAGAGACAGGCACACAGAGAGACAGACACACAGAGAGACAGGCACACAGAGAG
ACAGACACACAGAGAGACAGGCACACAGAGAGACAGGCATACAGAGAGACAGACACACAGAGAGACAGACACACAGAGAGACAGGCACACAGAG
AGACAGGCACACAGAGAGACAGGCACACAGAGAGACAGGCACACAGAGAGACAGGCACACAGAGAGACCCTCAGTGATTTACTCTGCTATTACA
AGAAGATTCAGACTTCCTACCTTGGCTTCTAAGAGGTGTAGGATCTGACCCTCACTACTTTCCTGTGCCCCACTGTCTGATGTCTCCACTTTTT
CTACCCTACATCCCATGTTGCAATATGGGAATTCTATGAAGTTATTTTTTTAGTAATAGAATAAAGGATGAAGTAATAAAAAGTAAGCTGGGCT
GTTTTCAGATACACTGGTGGGAGCAGCACTCACTGCTGACAAAACGTTGTTTCCCGTTGCTTCTTAGACCTTGCCCCAGGACCTTTGCACATCC
TGTTTTTCCATTGGGAATTCATGTCCTCACTTTCAACATTAGTTTTTCCTGTCTGTAGGGGTTCTCTCTGACCCTCTCATCCGCCTTGGGCCCC
TGCCTCCCTTCTCTAGTGCTGGTGATAATTTATAAGATTTATAATTCTCTTGGCATCATGTGGCATTTCTCAAATAGAAGAGAATAGCCCTCTT
TACCAGAATATTCTGGGAGCACAGCCACCCGGAATCACTTTAGTCCTTGGCGTATTTTCATACGGTGCCTGGACGTCGTCTTCACGTTGGTGTG
GGTATTCAGTGGATGCCTCCGTGGATGGCATTTCCTGCAGGTGGCGTTCTAATGCCTCAGAAACGGTCCCTGTCTCCCTGAAGTACACCTGAGC
TCATCCCCATTTTCTTTTTTTCTTTTTTCTTTTTTTAATGCTCTTACACATTAAGCACGATCAGCGCCAACTTCTTCTACTAAAGGATCATGTT
AGTTTTCCTGTGGACACTACATGGCCCACTCACCTTTTCTTCTGGGGAAGCCTGAGCCGTAGAAGCAGCTTCTTAATGCCCAGATCTCGGCTCC
CTTCCGCAGGTTATCTGCAGGGGCTCTGCCCACGAGGAAGTCTCCTCAGGGCTAAGTGGAACACTCATGCTGTCCTTGCTCTGGTGTTTCCATG
CCCTGACCTCATGCCACGAGACTGATACCCTCAGTCTTTCCTGGCCAAGGTGCTGAAAAGCCTAATCAGGCTATTTGTCCTGACTTTGCCCTCT
GTGTGTTGAAGAACAGTCAACAAGTAAGCCAACTGTTTCTCGAGCTTGCTCTGTACAAGATGCTGGGATGTGTGACCCCTGCCAGTTGTCTAGC
ATTTTGCAGACCCATTTCGGTGAGCTGTGGGCCATCCTGCTGGGCCATGGCAGCCTGTGGGCTGTGATAATGGCGGCATATGCATCCTGCGTGC

ATGCTTTCTGACGACGCTCCCTCCCTCCCATCCATGGCTTTCTTGTAGCCAGCCATCATCATTAAATTCAAATAGAAAATAGAATTTGCTCTCT
GAAATCCCAACTATTCTAGAGAATGTTTATTGTGGGTGTGTTTCAGGTGTGTGTGTGTGCATGCGAGTGTGTGCACATGTGTGTCCAGAACACG
TAATGGAGGTCAGAGAACAGCTGTGAAAGCCGTATCTTTGCCTCCGTCTTCCTGTACCCCAGGAACTGACCTCTCAGGTCCTCACAGTGTACAG
TGGGTTCCTTTAAGTTCCTTCATCTACTGAGCGATCTTACTTCCCACTCTACATTTGTGAGTCTCTGTTTAGTACAGTATGGTGTGTTAGCCCT
GCAGTGCCCCTCGGCAGTCTGGGCTGGCCTCCTGGGTCTTCTGGAGTGGCCTGGGCTCTTGGGCTGCAGTTTTGACATTGCTGCTTTGAGGTGG
GTGGCGGGGAATAGGGGAAAGGGGGTACAGCCCTTTCCAGTGCTGCCTCCCAGAACACCTTGCAGTAACAAAGCCACTGCTAGAGTTGTACTGT
GGCAGCTCCTAGTAGACTTTCTTTCTCTTCTCTGTTAACTTGTGCTCCACTTTTTCTTACTTCCCTTCCGTAGTTGATTTTGGTGTAAGCGCTC
AGCTGGACAGGACGGTTGGACGGAGAAATACATTCATAGGCACACCATACTGGATGGCTCCGGAGGTCATCGCCTGCGACGAGAATCCAGATGC **Exon 7**
CACTTACGACTACAGAGTAAGACCCCTGTCCTCCCCCCCCCCCCAAAGCCCCTACCATTATTGTCATTGCCAGGCATTTTTGTGTAAAGAGTTT
CATAACTTCTCTTGGTTAGACAAATAAATTGAAGTGATTATAGAGTGACCTCTAGGGGCTTGAAATCATAGTGCACTTTTTTTATCCAGGCATA
AACTTATATTCATTGTCTGAATCAGCTTGACTGTGCTACTGTTTTATTTGCCAATAAAGAGAAAACTTTTGCTTTCATGGTTATAGTCCTGGT
TAGTAGGTTAAAATTGAGATCATGTTTTAACTCTTCTCCTGTATTTCAGTATTTGTTGCCAATATCCAGTTAATGGGGATTAATTTAAACCATT
ATATACCCGGATTTTCTTTTAATTTGAATAATTTAAATATGTATTGCAGACTGATTTTTCTATTCCTTAATACATGCATAAAAGTTGAAACACT
TTTCAACATTATCAAAATAATATCATGCTTAAGTAAATAAAAATAACCCCTAGGGCTGGAAAGGGAATAACTTCATAGCAAGGTTTGTTTGTTT
GTTTGTTTGTTTGTTTTTACCTGAAGGATGCTTTTATTTCATTTGTATGACTGTCTAGAAATGTAACTAGGCATCTGGAACATTCTCTTCTGTC
TTTGCAGAGTGACCTCTGGTCTTGTGGCATCACGGCCATCGAGATGGCAGAAGGAGCCCCCGTGAGTAGCTTCCTTCTTTTTGAGTTCATTTG
TTCTGTATAAAGTGATTACCTGTAAGAACTCAAGGACACTGGGCAGAACCACGCATGATTGAACGATTAGATGAAAGACCTATGATCAGGGGTG **Exon 8**
CCTGCAGATGTACAGGGAATTCTTGTTAACCAATATTACTTTTGTGGCTCTCTAGGTCTCTAACCTATTCTAGAAAATGAGATGCAGCATCTGA
AAAAAAATTTTAAAGTCTTTTTATTTCCTAAAGATTTATTTATTTTTTGTTTTTTTTTTTGTTTTTTTGTTTTTTTTTGTTTTTTTTTTTTTTT
GGAGCTGGGGACCGAACCCAGGGCCTTGCGCTTGCTAGGCAAGTGCTCTACCGCTGAGCTAAATCCCCAACCCCAGATTTATTTATTTTATTTG
AGTACACTGTTGCTCTCCTCAGACATACCAGAAGAGGGCATCAGATCCCATTACAGATGGTTGTGAGCCACCGTGTGGTTGCTGGGAATTGAAC
TCAGCACCTCTAGAAGAGCATTCAGTGCTCCTAACTGCTGAGCCATCTCTCCTGCCCCCGCAGCATCTGGAATTAATAAGAGCTATTTTTTTTC
TTTTGCATTTAAAGAATATTGATTGAAACAGTCCCTAACCTTTGAAATTTTCTTATTTTGAATTTACATATTGAGACATTTACTTCTTATTTAT
GGAATCTTCTCTGAAGATTTGAATTTGCTTTTTCTTGGGGGTTAATTCAGTAAACTGGTCAATCATAGAATTGACTTTTGATTAGGGTTGTGAT
AGAAATGTAGAAGAAGTAAGGAAGGAAAACATGTTTAGTATTTCTCCTGGCCTGCTAGGAAAGGTATGTCCAAACACTGAATGCTGCCTCGTAG
CTAGTTAAGAATAAATATTCGTTGAGTGAATGAAACAGTACAGACAGTTAAGAATCAGTTGCAGTGCGGAGGTGGATGCCCACTGAGAGTACTC
TGTCCCTTGTGTTTCCTTATATGGTATTGAGACGGACGTATTGCTATGTCATTTGTCTCAGTTTCCCTTCATGCTGGGATACTGGTGTCTGCTA
GTCTGGTCTACATTCCCAGCTAACTGCCTTACAAGTGTGCCCTGACCATACGCCTGATCTTCTGTAACCTGCCAGTATATGTGTCTGCTGAACT
CGCCTTTCTTGGAGTGAGTTACATAAACAGCAGTAACTGCACAGAGGCCAGCACCTCCGTTTACACTCAGCTTTCCTCTCTCCTCCGGAAAAGA
GATGGAGTAAAAGACAGCAATGTCTGGGGACTCTGTGCGCTGTGTTTAGGCGTTGGTTCTGTTTGAGGCTCTGGATGTGCCGGCTGCATTTCTG
CATTGGTTCCGTTTGAAACACTCTTGCACTGTGTTTAGGCATTGTTACTGTTTATAACACTGAAGGCCTTTTCAAAGCTGTTCTGATCCTTAGA
CCAACCTTATAAAAACAGCCATTTTCATTATAATCTTAAAGGTGATTTAAAATTTAAAAACAAGTATTTTTTTCCTTTTAAATTTGAATTAATA
TTTTTCTTTGATTTATGAGACCTTTCCAGTGCCGATTTGAAGTTGTCTATTTTTGGTCTTCTTATATTTTACAGTTTTTAAATGGTAAGTTCAA
GGTATGGACTAGGGTTTGTATCTAGGTTTCTCAGCTGTTTGGTATCCATGGCAATTACTTGTGCAAGAGGTTGTTTGTGCGTGAACCCCAGAGG
TCAGGAGAGGGTAAATCCGTGCCTTACTGACACAGCTGTCTCCTTTCCCAGCTCTCTGTGACATGCATCCAATGAGAGCGCTGTTTCTCATCCC
CAGAAACCCTCCTCCCAGGCTGAAGTCAAAAAAATGGTAAGCCATGTATGGATTTTCCCCTACTCCCATCCCTCTTCCAGATCCCTCCCTCCTC **Exon 9**
TCCTTTCTTCCCTTTTCTTTTCTTCTCCCTCAACCCCTATGGTTTCACTTCGTTCAGGAATAAAAATCTGGCCACCCTAACTTCAGTTCTACAA
AGCCAGCATCTTCAAACAGTCTAACATGCATTGATTTCTTTGTGTTGTTCTGGATGCATCAATTTAGACGCTAATGAACAAGCAAGCTCTGAAA
CCCCATAATAAGAAGACAAGGTGATATTTTAATGTTTGAGGGTCTTTAAGTGTACTGTGTTACAGAGCAGTTCCCTACTAAACTGGCTACGATG
TGTTGAAAGCCCTCTGGCAATAGAGTTTAACATTGAGGAAAGGGGTCTGGGGCCATGGTGCATGTTTGCAGGCCCCACGACTTATAATTATGAC
TACCCCAGGACGACCATTTGAGCCTAGGCATTTAAAGCATTCTGGGTAATAGAGCAAGATCCTGTCTGGGTGAGGGTGGGTTGCAGATACCATA
ATGATGTTTTATAAAATGTACCATCCAATTGTAGGTTTGTGTATGAGTCTTCACGCACCAGGCTGGGAATGGGAAAAGGACAAAGCTCTAAAAC
TATCTGTGGCACAAATGAGTGTGACAAGAGTCCCCCAGCCCCACTGTGCACACGCATGCACACACAAAGTAAAACCTGTCATTTCTGCCTCTGT
TTTTTAAACTCTGGGACTTAAAGAATTGAGCCAGTGTAAGGCCGGTTGAAATGTCAGTACATGTCCTGCATTTGCCCCACTGAGGCTGGCTCTTT
GCTTGTCTCTGTTTTTTGTGACTCCTGTGGTCATCTGTGCGCCTCCCTGACCCCACATTAGAGAAGCCTTTTCTCTGTCCCTGTGCCTCTGCCT
GCTTTGTGATATAGTGGTTAATGAGTACATTAGACTAAACTCTGTTCCTTTAACTGGCTGCTTTTATGGCTGTGAGTGATATGTTAGCGTGCA
CTGCAGGTGTGACCACAGTAACACTGCTGACTGGTGCAGTGCATTGTTAGTGTGCACTGCAGGTGTGACCATAGTAACACTCCTGACGGGTGCA
CTGCATTGTTAGGGTGCACTGCAGGTGTGACCACAGTAACACTGCTGACTGGTGCAGTGCACTGTTAGTGTGCACTGCAGGTGTGACCATAGTA
CCACTGCTGACTGGTGCAGGTACGTTGTTAGTGTGCACTGCAGGTGTGACCATAGTACCACTGCTGACTGGTGCAGGTACGTGTGCACTGCAGG
TGTGACCACAGTAACACTGCTGACTGGTGCAGTGCATTGTTAGTGTGCACTGCTGGTGTGACCACAGTAACACTGCTGACTGGTGCAGTGCATT
GTTAGTGTGCACTGCAGGTGTGACCACAGTAACACTGCTGACTGGTGCAGTGCACTGTTAGTGTGCACTGCAGGTGTGACCACAGTAACACTGC
TGACGGGTGCAGGTACATTGTTAGTGTGCACTGCAGGTGTGACCACAGTAACACTGCTGACGGGTGCAGGTACATTGTTAGTGTGCACTGCAGG
TGTGACCACAGTAACACTGCTGACGGGTGCAGGTACATTGTTAGTGTGCACTGCAGGTGTGACCACAGTAACACTGCTGACTGGTGCAGTGCAT
TGTTAGTGTGCACTGCAGGTGTGACCACAGTAACACTGCTGACTGGTGCAGGTACGTTGTTAGTGTGCACTGCAGGTGTGACCACAGTAACACT
GCTGACTGGTGCAGTGCATTGTTAGTGTGCACTGCAGGTGTGACCACAGTAACACTGCTGACTGGTGCAGTGCATTGTTAGTGTGCACTGCAGG
TGTGACCACAGTAACACTGCTGACTGGTGCAGTGCATTGTTAGTGTGCACTGCAGGTGTGACCATAGTACCACTGCTGACTGGTGCAGTGCATT
GTTAGTGTGCACTGCAGGTGTGACCACAGTAACACTGCTGACTGGTGCAGTGCATTGTTAGTGTGCACTGCAGGTGTGACCACAGTAACACTGC
TGACTGGTGCAGTGCATTGTTAGTGTGCACTGCAGGTGTGACCACAGTAACACTCCTGACGGGTGCAGTGCATTGTTAGTGTGCACTGCAGGTG
TGACCATAGTACCACTGCTGACTGGTGCAGTGCATTGTTAGTGTGCACTGCAGGTGTGACCACAGTAACACTGCTGACTGGTGCAGTGCATTGT
TAGTGTGCACTGCAGGTGTGACCATAGTACCACTGCTGACTGGTGCAGTGCATTGTTAGTGTGCACTGCAGGTGTGACCACAGTAACACTCCTG
ACGGGTGCAGTGCATTGTTAGTGTGCACTGCTGGTGTGACCACAGTAACACTGCTGACGGGTGCAGGTACATTGTTAGTGTGCACTGCAGGTGT
GACCACAGTAACACTGCTGACTGGTGCAGTGCATTGTTAGTGTGCACTGCAGGTGTGACCACAGTAACACAGAATGGTTTTCAGACTCCTCACT
GGTACTTTTGTCTTAGAAGTTTGACCGTATGTTTCACTTCCTTTTACAGGTCAAAGAAATTTTTCAGCTTCATAGAAGGCTGCCTGGTGAAGAA
TTACATGCAGCGGCCCTCTACAGAGCAGCTTTTAAAACACCCTTTCATAAGGGATCAGCCCAATGAAAGGCAGGTTCGAATCCAGCTTAAGGAT
CACATAGACCGGACCAGGAAGAAGAGAGGCGAGAAAGGTATGGAGGCTCCTTTATGTGCTATGTCGCCTTTCTGACTTAACATTAAGAGGTCTC **Exon 10**
AGTCCAGTGTTGAAAGCACAGGCTTCTCTCTGACATTTTAAGTGCAGGCTGCTGAGCTCCACACCCTTCCCAGGAGCTCTTCTTTCCCTCTGTG
CTCCTCCTAACACCTTCGCCTCTCCCTCTTCCTTCTTCCCGTCACGTGGTCCTAGGCCTTCCTGTACGCTTACACAGCAAGAGCAAGGTCACTG
CAAGCCAGTGCTCCTCCACAGCCTGCCTCCCCTCTCGTGGTTCCTGCTGGATTCATGTCATATCCCTCACACTGATACACACGGGGAAACAGTG
AAATACGCCTCTTAGAACACGCAGTATTTGGCTTTCTGAGTCTGGGTCTGGCGCTTGTTCTAATGTTTGCCCGGTGCATCCGTTTCCCTGAAGG
TTCTTTGGCTGTGCAGAGTCTTCTGTTTCATATGTATGTTTGGATTACTTTTTTAAGTACAGCAAGATATTTCTATGTAGTGTCATTTTCAAGT
GGCGTAATGTTTTCATCGTAGGCTCTGCATCAGGCTTATTCCTGGACCCTTGTTCTTTCTCAGTGAGTTTGTGGCTGTCATATAGGAAAGCTGC
TAGGTTTTATATGTTAATTCTGTATCCTGCAGGGTCTTGAAGAGTCTGAGTTAGCATACTTTGAGTTCTTTCTTTTTACATCTTATTCTTTAGT
TGGGCGGGACTTGAAACACTGTATCAAATAAGCGTCTAGTTCTAGGTTTTAGAGAATATGTTCACTGCCCCTCATCCCATACAGGGTTGTTTGT
AGGCTTGCTGTATGTAGCCGTCACTGTTTAGAAGTGTACTCTTAGGCTTTGTGCTCCTACTTCCTTCAGGACTTTAAGTGGGGCCTCACTACAT
AAGCTGCAGATGGCTGGTGAGCCTTCCATCTCAGCCTCCCCGTGCTGGGGTGTGTATGTGCCAGGAGCCGGCATTCTTCTCATTTTCAGCTCTG
TCCTGAAGCTTATTTATGTCATGTATTACATTTTTTGTTTGGCATAGCTCTGCGTCCCTGAGATAAACCATTTTTGTTGTGGTTTCTAGTCTTA
ATATATTCTTGAATATAACATTCAAATAATTTTGTTGGGAATTTTCCATCCAATGTTTCTTAGAGAAATGAGCCTTTAATACTTTTTTCTTAAA
GATTTGTTTATTTACTTAATGTATATTAGTATACTGTTGTTATCTTCAGACACACTAGGAAAGGTCATTGAATCCCATTTCAGATGGTTGTGAG
CCACCATGTGGTTGCTGGGAATCGAACTCAGGACTTCTGGAAGAACTGTCAGTGTTCTTAACTGCGGAGCCATCTCTCCGGCACCTAATGGTCT
TTTCTTACACAGTTTCTATTGGGTTTTGCTGGTAGGTCGTCTTGGCTTCATAGGATGAGTCTGCATCTGGCCAGCATCTGTCCTTCCTTAGACT

TAGTTTTATAAGAATTCTTAACCACTGAACCAGTCTCAGTACTTGTTAGGGACCCATTTAAATGCTTATATCTTACAGATTTAATTGTGGTCAT
AGGCATTTAGAAGTTTATTTATCTCTCTATTTGCCAACCTCTGGAATATAAGTTTTCAAAGCATTTTCTAATGATCTTTTGGGTTTCACCAGCC
CTATGGTAACAATTTGCTTATCATCTTTATTTTATTAATTTGAGTTTTCTCTCTGCTTTTGTTAGTTCAACTTGGAGTTTGTCGATCTTGTTAC
TCTTTTCAGAGAACTGGATGTGGCGAGTACTTTTGTGTTCTGCTCTTCCTGTGTCCATCTCAGTACTTTCTACGCCGCTCTTGTCCTATGCTGA
GTTTGCTTAGTTCTTGATGTCTCTAGACCATTATTTGAGATCTCTGAATTTTTACCACATCTGTTATCTCTGTCATCTGTGTATCGATTGTCTT
TTTATATCTGTCAGCATAATACTTTTTCTGAATGTAGATATGCCAGTGCCGTTGAGTTACTTCTCTTGTGTGCACTCTCTCACAGAAACTCCAA
CTTACCAGATTTTATCACTGGGGAAAGACCAGCTTTAGGGAGTGTAGCCAGCTCAGAATTCCCTACCTTTGTTGAGAAGTCAGTGGCTCTTCTC
CTCATGCTGTTTTTACAAAGAGCATCTTGTTTTTGGTCTTTCTGACTCTGAGAATGCTTGCCTACACAGGCACCTGTGAGGATCTGAGCTCTCA
CGTTTCTCTTGGCCTTGTCTTTCAGATGAGACGGAGTACGAGTACAGTGGCAGTGAGGAGGAGGAGGAGGAAGTGCCTGAGCAGGAGGGAGAGC
CAAGGTATGGACACCAGCCAGACCAGGCTCCACCGATGCCCTGCACTCTGAAACTCCTATGGAGCAGCGGGCCGCACCAGCTTGCTTCCTATCT **Exon 11**
GCCATTTTTCTCACCTCAGCCCTCTGCGAACAGTTCCCCACCCCAAACACTTGCCTCGTTTCTGGTTTTGTTTTTAACCCAGTTTTATGTTTGA
GGCTGAGGAAATAATGCTTCTGACATTTCTTGCTGCAGAAGGCACAAATGGTACAAGTCTGCTGCCTGACAGAAGTTGGTCCCACACACAGAGG
AGCATGTGGCTCAGAGGCCAACAGGCCCTCTGATGAGAAACTCCTTACCGCCTTATTTTCCCTATGTGCCAGTGGGGTTGCCAGCCTTTGGCCT
TTTCCTTCATGTGTCTTCTACCTGGTTTAGCTCAGTGAAGCCAGGCAGAAGGCGAAGAAGAGTGGGTTCCTTTCTTATACTTTTACTTTAGTGA
TGTGCTTGCCTGCACCCGTTCTAGCTCTGGCCTTACCTTTGGTTGGAATCCAAACAAAACCAGGGTGAAGGTTACACTGAGACCCATGGGAGTT
GTGAGCTGCAAATAGAAGTTCAGAGAGGGTCACAGAGGTTTAAGGAGGTGTTTCTTGGCATTCTGTATGGTACAGGCGTGGGGACAGCCCCCAA
AGCAGAAAAGTGATAGCTGAGTTTTCTGAGCGAAGCCCATTCATTCACAAGGCTGCTGCTGTCTCTGATGTTAAAGAATGGTATCTGGGAGCAA
AGCAGGCCTTGCCGCAGACCAGGTGAAAATGAAGGGGTGATGAGACCAGGGTGCCAGGACTTCTGTGCAGTGATGTAAAACCAGAGGTGAGAGA
AGAGGAGAGTCCCAAGCAGGAATGCTTATCTACTAGGGCTGAGTTTTCAGACATTACTAAATGACACAGGATGATGCGAGCCTCTGGACAGTAG
CCTCTGGAGCAGTAGGCATAGTTGTCCTGGTAGACATGTTGTTTGAATTCCTGGGCTCAGTCCGTGACCCGTGAAGGGAACTGGGTATGTGCCC
ATTGGGTCACACCCTCAGAGGCCCAGGTGATGCATGGAGCCCTGATAGACCTGTCCCCTACAGTTCCATCGTCAATGTGCCTGGAGAGTCGACT
CTGCGACGTGATTTCCTGAGGCTGCAGCAAGAGAACAAGGAACGGTCTGAGGCTCTGCGGAGACAGCAGCTTCTGCAGGAGCAGCAGCTCCGGG
AGCAGGAGGAGTATAAGAGGCAGCTGCTGGCTGAGAGGCAGAAGCGGATTGAACAGCAGAAAGAACAGAGGAGGCGGCTGGAAGAGGTAGAATG **Exon 12**
GAGAAGAGCTGTGTTGCCTTTTCTCTTCCCTACCCATGCTCTCACATCAGACACCCAGTTAGCTTGTTGAGGAAAGGGGAGCTAATAAGTTGTG
AGGTAGTCCGTCCTTAGCCTGCTGCAGCGGCAGAGAACTCCTGTAGGGATGCTTGGCCGAGCACCAGCAGGCTTTGCACTCCTGGAGGATTTGC
CTGATACTTAGGTGCACAATGTGAGGTGCAGGACTGCTCGAGACAGGTTTCCACTGTGCTTAAATTGACTTGCTTGGACTTGACCTATTTTCTC
TGCCATTCTTGGCTTTCGTGACCCAGTTTTATATTTTTCTGAAACTTCAGACGTTTGCCTTAACTTCTGGGGTGGATTTAAGAGTAAAGCATTC
AGACCAGTGCAATGGCTCAGTGCAGTGGCACCATGGATAAGATGTTTGCACCAGCAAGGCAAATTGAGTTTGATCCCCAGTTCCGGGTAGTGTA
GAGAGAAAAGAATCAACCCCAGCAAGTTGTCTTTTTTCTGACCCCGCCACATGTGCATGCACATGGCCCCGTGTTCGTTCGATCGATCTATCTA
TCTATCTATCTATCTATCTATCTATCTATCTATCTATCTATAAAGAGGGAGATTCAGAAAGCAGAGTAGTCAGTAAGGTTACTTGTATTTTATG
GTAGGTATAGTTCACATGATTTTCACAAGGGCATTGATACTGTAAACCCTGGCAATGTAATTATTTATAGGTTATTTTTTATTTTTAGTTCACA
CGGATGTACTATTTCCCTGTAATTCACTTGAGAATCAACACTTTTTGATTTTCTTTTCTTTTTTTAAATTTTTTTTTTTTTTTTGGTTCTTTTT
TTCGGAGCTGGGGATCGAACCCAGGGCCTTGCGCTTCCTAGGTAAGCGCTCTACCACTGAGCTAAATCCCCAGCCCCTTAAATTTTTTTAAAGA
TTTATTTATTTATTATATAAGTACACTATAGCTGTCTTCAGACACACCAGAAGAGGGCATCGGAACCCATTACAGATGATTGTGAGCCACCATG
TGGTTGCTGAGATTTGAACTCAGGACCTCTAGAAGATCAGTCAGCACTCTTAACCACTGAGCCAGCTCTCTACCCCTGATTTTCTTTTCTTGAC
CTCCTATTACTTTATATGCCACAGCCCCGCAGTTCCTCGTCCATCATTTGACTTGCATAGCTATTCTTTTCCTCCTGTTTTTTTCTAGTAAAAG
GAACATCAAGATGAAGCCCTGTAATGACATTTCTGGTGGGATTTAGTTTAGATAACTAAACATACTACTTGTCGCCTAGATCACGGGATAGTCT
GTGTTGGCACTGGTGTCTTTGACCATTCTGTGGATCTGTTTGCTGCTTGGAGAAAAGCAGTGCCTCACTGAAGGGATGATGTAAATAGTGTTCG
GTTAGGACTTTAGGGGGCGAGAAAGTGTTGAGTGCTTGCATAACTTTCAAAATGTACCTGTTGCAAAATTATTCATAAGACATTTGAGATGATT
CTCACTGTCCTGTGACCAGAGCAGAAAACCAGAATGAGCTTGATGCTGACTCGAGGTAAGGGATAGGAGGCTCTGGCCATGCCCACTACTGTGT
TCCTGGGCTGGTACTCCACTGCCCAAGTGGGTGCAAAGGTCACCCTGTGGGGCTGTAACTTGTCTTAGGGACTAGATCAACAGAGTTTGTTTGT
AGCCCAGGTGGTGAGGGTGGCTTTCAGGCTGCGGTAGTGACACAGCCAGCTATTGTCTGTGGGGTAGGCCACAAGGATGACAGGGTATGTGTTG
AGGCCTGAGAAAGATGCCTGGTGAGTTAGAGACGTCCCCCTCACACCTCTTCACTGGTCTGCTTGGCTGTCGCTTTCCTTAAACACAGCGAGTG
ACTGGAGAGATGCTTACGGGTTAGGAACTCTTGCTCTTGCAGAGGCCTTGAGTCTGGTTCTCAGCACCCACATGCAGCTCACAACCATTCTTGA
CTTCACGGCCACCAAGCTCACTCATGCTTACATATGCATACCTGCAGGCTAAACGCCCATTTGCTAAAAGTAAAATAAGTCTAAATGAAAGAAA
AGAAAGGAGGAAAGGCGGCATTAGGGAAATGAGATCGAGGGAATTCTCAAACAGTGAAAACCCACAAAAACAGAGCTAGAACGTTCCCCGAGCT
TCTAGGACATCATCGCTACTGTAGCAAACGCGAGTGCTGTGGAGAGGATTCGGAACGGGTCCTGAGCCCCACCCAAGCCTTATATTATATACCT
TATATTATATTTATATATATTATATAAATGTTTGAAAAGCTATTTTTATTCACTTTGTCCAGTGGCTAGCCTCAGTGGGACAGGGTTGTGAACT
GTACCTGTTTTCTCTCTCTGGGCGGGAGAGCGGGTGAACCATCGAGGTACTGTCCAGTGCAGTGGCCTCGAGCAGACTCACTTCCGTACTCGTG
CAAACCGCAGTGTAGTTCTCAGTGGTGAGGTGTGACTAATGGGCACATGCTGGACAGGCCTCGTTAGAGACTCTAACATCTCCCACATTCACAG
GAAGAGCTGACAGAGCTTAGACGAATGCCCTGTACTGACAGGTGGGGTGCCATTGTGTACTGTCAGTCAGGGCAGCTGCTATGGTATCCACTGC
TGTGTGCCGTGCGGATGTTAGAGCAGGCTGAGTCTCTGAGCTGAGTGTACCCCGTCTGTGGGATGCTGGGCGACATGACGCTGTAGAAGGTGCA
TGGGCAGAGCAGCCATTCTCACAGAGAGAGGCTTTCTGGATGGTGACCATGGACAGTTGGGTTCATTTTTCTGTTTTCTGTACAGTAGGGATGA
GATCCTGACTTCCCAGGGTTCTAAAGAGAATTGAACCAGGAACACCAACTAGCTTGAAAGGCTAAGTGTTTGATACAGAGCCCAGGGCAGCGCC
TTCACGCCTTCCTTGTTGGGAGCTTTGAGCAGCGGCGATGGCGCTTGCTTTCTTCTGCTGCTTTTGATTTCTTTTTGTTGTTCTCTCGCGCATG
CTTCTTACTTTCCAGATATTTATCTTCCTTTGTCCTTTCTTCTGCCCTTTATTCATCTCTTTCTTTTCGTTCTGATTTAAGGGTCCTAGGTAGT
TAGTGATGCCCACGCATGACAGATTTGCTGACCTCTGAGTGCCACAGGGTAGTGAGGACGCTTGCTTTCAGAGTGGCAGACCGAGCCTGCGAGG
ACTCATAGAACATTACGCCTCTGACCAGTCATCATGAGGAAGCTGTTGGGAGCAGGGTGGTGTAGGGTAGTGGACCACTACAGCTAGGGTCCAG **Exon 13**
AACCCACCTCACTGACCTGCTCATCACCGTTGGCAAATCTCGTGAGAACTCAAGTTGCAGGTTCCTGCTAACGACACCCGTTCTACTTCTGCAG
CAACAAAGGAGAGAGCGTGAAGCCAGGAGGCAGCAGGAACGTGAGCAGCGGCGGCGAGAACAGGAGGAGAAGAGGCGTCTGGAGGAACTGGAAA
GGCGGCGTAAGGAAGAGGAAGAGAGGAGACGGCAGAAGACGAGAAGAGGCGAGTGGAGCGGGAGCAGGTGAGCAGGTGTCGGCCCAGGTGTCT
GTGGCTGGGCCACACACGCATGCACGTGTGTGCACACTCAGAGGTTTAGGTGTCTAACGGTTCTGGAAACTCCAATGACCTGACTCCCTATCTT
GAAGAAGAATAAAGATTGTTACAATGTAGAGACCCTGGGAAATAGAGTCCTGAAGAGGATGACTTGGGACAGATGTCTGAATGTACGTTCGCAG
GCTTGGCTTACATCCATTGAGCTCCAAATGTCTGGGTGATGCTAACTTTCAGACAGGGAAATTGAAAAACCAGCTGTAAGGAAATCATTAGTAG
ACGCTTAGAGTACGTACTGGCCGAGTGAGTGCTGTAGTCCATGTTTGCAGGTCTATTTGTAGCATTAATGATTTTTGGTGTGGTTTAGAGATGA
AAACCAGATTCTGTAGACTTGTACTTTTATTATTGCCTTGGGAGTTTATTTGTAAATTCCTTAGATTATTTATACAAACAGAAGATAACTTTTC
TTTCTTTATATGGCTTTCCTATCTTCGAATTAATCGATCTGTGCTTTCAGCTTTAAACAAAGAAGAAAGTCAATGAATGGAAGCCATTTTGAAT
ATGCTCAGAGTTCATATTTAAAGTACTAACAACCATCGTCTTATCTTCCAACGCAGTGATCCAGCTACTACTCTCGACGCTCCTCCTGGACCAC
ATGCACCCATGTTCCTAGACTCTCCCTGATGTAGCCTGCCTGTTACAGTTGACTTCTTGGCCCTGGGCTTGTTTGTTTTGTAGTTAATTATTAA
TGTTTTTATTGTGCTTACACCTGGCGGCTACAAATCTGTGAAAATGCTACCTGCTACTGGTCAGAAGTCACCCAGTCCACGTTTGATTGTGACA
AGAAAATTGATGGTGGCATATTAGTTGTTAAGATTTTGTGAATAGGTCTAGAGAGATGGCTCAGCAGTTAACAGCTCTGGCTGCTCTTCCAGAG
GACGGTGGCTCAGTTCCAGGCACCCACATGGCTTCTTACAACCATCTATAACTTCAGTGCCAGGGATCCAGTGCTTCTTCCAGCAATGCATACA
ATACAGCATGGTGTACAAACATACACGCAGGCAAAACGCTCATAAGTATAAAATATAAGTGAACTTTAAGAACCTTGAAAAAACTGTTGTGAAC
AGCAAGTGCCCTGCACTTTAAGGATTTCTTAAAATTGATTGTTTTATTCTGCTCCCCCCCCTTTGTTTTGTAAATAGTTCTTTGTTAACCTTGG
TGTGGTGCAGGGTGCATTCCCATGAAAGCGTGTATATGATGTATTCTCTATGCACATTGTATGTTGGGATCTTTGTGGTTAGCATAGCATGTGA
TTTCCCCCAGTTTTCTTGCATTTTTAAAGTCAGAAACAAATGTTCTATAGTAACTTGATCTTTCAAGTTATGACTTAATTCTGTTTTTTTCTTT
TAAAGAAATGATTTGGGACTCAGGTTACTTTCTCAGACAACTAAAGCCACATATCTGTCACACTTGGTGCCTTAGCATGGCTTTGGGCGTTCAG
TTTGGTTTATAAGAGGTGGTGTTCTGAGTGTTGGCAGGCATTGTTTCTCTGCTGTCTGGTATTCTTCTGACCAGAACGATTTGTTAGCTCACAC
ATCTGTTCAGGGCATATGGCATGAAATGGGGTGGTCAAGATAGACACGGAACCCACCTCCGTGGGACAGTTCTCCCACATGCCTGTCCATTGCT
CCCCTTCCAGCGTCTAGGACTAGTAGGTCCTCTTGCTTCATGTGGGGCTGGAAGTGGTTGTTGTTTTCCTGCTGAAGAGGAGGCTTCTTGTAAA

ATGATTTTGCTCTTAGGAGATATTTTATATCTAGCTCATGTTCCATTTCTCAAATGAAGAAATGGGTAGTGTAGACGGTTGAATATGTGCAGCA
GGTGCGGCAGCTGAGTGTGTGCAGCAGGTGCGGCAGCTGAGTGTGTGCAGCAGGTGCGGCAGCTGAGTGTGTGCAGCAGGTGCGGCAGCTGAGT
GTGTGCAGCAGGTGCTCCAGCTGAGTGTGTGCAGCAGGTGCCGCAGCTGAGTGTGTGCAGCAGGTGCTCCAGCTGAGTGTGTCCAGCAGGTGCC
GCAGCTGAGTGTGTGCAGCAGGTGCTCCAGCTGAGTGTGTGCAGCAGGTGCCGCAGCTGAGTGTGTGCAGCAGGTGCTCCAGCTGAGTGTGTGC
AGCAGGTGCCGCAGCTGAGTGTGTGCAGCAGGTGCTCCAGCTGAGTGTGTCCAGCAGGTGCCGCAGCTGAGTGTGTGCAGCAGGTGCTCCAGCT
GAGTGTGTCCAGCAGCTGCTCCAGCTGACGTGTGTAGCAGGTGATGCATCTTGTCGCCAGTGGACCTGGGTTAGGGTTTCCTGATGTCTGTACG
AATAGGATGTTTAACTGCAGAGTTGCATGTTCTCCCCTCTCACCCATCCCCAGTACTGGAGATGGGTAAGTAAGGGCTTTCAGCGAGTCAAGGC
AGCAGGTTACCTCTCTTCTGTGCTCTGTATGAGATCACCTGTTTCTACTGTGACTCAGTGGTCATTTTCTCCCGATGTCACGTCATCAGTCTGC
CCATGCACTTATGCCTGTGCACACTACACAACTACACAGACACACAAGCTCCTCAGCATGGGCTTGGCCAGTTTACCTGTAGAGAAGTGCCCAC
CGTCCATGACTGCTGACCACATCTTCGACTGTAGTCATTACTCCAGGGACCTTTGGGATCAGCAGCAAAAGCACAAATTCTGTTTGGCTGGTTT
AGGATTCTTGCTTTTTTTTGTTGTTTGTTTGTTAGGTTGTTGTTGTTTTTTTTTTTTTTTTTTTAATCATCTTAAACAGATTTGGGGGTCTCAT
TTTTTAAAAAATGTCTAATCCATGCTTGTTGAAAACGACGCCTTCCAGTTTCTGTGGTGTTTGTCATCCTTATGAAGAAGCCAGCCCCCTTTAA
AGTGTTGCATATGATGACAGTGTAACACCAGCCAGTGCATCACAGGAATATGTTAGACTTCTTCTAGCTTGGAGCCTTTGCAAAAGCCCTCAGC **Exon 14**
CAGCAGAATGTCTTCACTTGATGAGTTTAGTCCTTGGCAAGCTTGCTGTCCAGCTTCTGGAGTGGTGCTTGGTTACCCCCACAGGTGACTCAGA **(M1)**
TGCTTCCCTTCCGGGTGTATTGACTGCTGGGTAATTTCTGTGTGACAGGAGTACATCAGGCGGCAGCTAGAGGAAGAGCAGCGGCACCTGGAGA
TCCTGCAGCAGCAGCTGCTCCAGGAGCAGGCTATGTTACTGGTAACGCCCCGCCTCTGTCCCATTCCGTAGCCACAGGGCCGGTGTCCCTTTCC
TAAGGTAGCCAGCTGTAGTGACTATCAAACCTTTTTGGTTTTGCTGAGGCAGCCAGCTTCCAGTGAAGATGTCCTCTGGTCACACTGTCAAGCA
CAGTGTTAATGGCATTTAGTTTATTTCCCATTAGCAGGTAACTCCTCCTGTTTGCTAGATGCCTATCAGCTCTTAAACAGAACGTGGCATTCAG
TGAGTGTGAAAACGCTGGAGGTGTCAGGCTCACACTGTATGCTATAGGATATTCGCTCAGTATGTGTGTATATATACCACTGCTTCTGATGTTT
AATCGAATTCCTTTACTCTGCAATGGTGTTACAGTTAGCAAATGCTGTCACATTTTACACAGGTAACATGCTATTTAAAATCCCAGCCATTTTT
CCTTGCTCTTAAACAAGGGGTGGAGGGGGAGTGCTATTTTAAAAAAACAAAAGTCAAGAAGGGATCCAGAGAAGAGGTGTGCTCACCATGGTCC
CCAGATTCAGTGTCACCCTCTCCCTCTCCAAGGAGTGTCGATGGCGGGAGATGGAGGAGCACCGGCAGGCAGAGAGGCTCCAGAGGCAGTTGCA
ACAAGAACAAGCATATCTCCTGTCTCTACAGCACGACCACAGGAGGCCACACGCACAACAGCCGCCACCCCCGCAGCAGCAGGACAGGAGCAAA **Exon 15**
CCCAGCTATCACGCTCCAGAGCCCAAGCCTCACTACGACCCTGCTGACAGAGCTCGGGAGGTATCTGCGTTCCCATACTGCTTACATGTTGGGC **(M2)**
CCGAGAGAGCTAGACGTCCCCTAATGACTGTGGAGAGCCACTGAATGTGTACCTTGAGTTCCAGAACAGACCTGGGTGCTAGCCAGGAGCTCAC
CTTCGTTTTCCCCGTGGATGCCTCGGTTAACAAATCCTAGAGAAATCAGAAGGAAACTATACAAAAAACAAAAAACAAAACAAAAAAAAAAAGA
AAGAAAAGAAAAGAAAATGCCCGGCACCGATATTCTCTGGTGTGTGAATGGGAGAAATGTTAGTCTGCTCTAAGCCTAGTGTGAGGATGAAGTG
TTTAGCCCGATTTCTTACTGGGTCAGTTGTTGGCGTTTGGATGACCAGAGAAGATGCAGCACAGTGTTCCTCTGTGGACTAAACAGAAGGGCTT
GGGTGTGTCCAGGAGCACTGCACAGTCCTGGCCTACAGGTGGCACCCTTCTACGTCCTCCATCACGGGGAGGCGAGTCTGGGACAAATTCTGTT
CCTTATAGAGTCACGAAACTCTAGGCGCTGAGGATATTCCAGAAGCTGCTTTGCTTGATCTTCATTTTCATAGTTTTTAAAAGTCCTCTATCAG
ATCTTAGCCCGGCTCTGCACCCACCGCTGTCCAGCTGCCTCTCTTCTTAGAGCTGAATAAAACATGTTTTCTCTCAATGGCTTTGCTCTTGTAC
GTTCTCTGCCTCTCCTTTCCTACTAACACGGAGCCATGTCTTCTGATTCTCTGACGCAGGTGGAAGATAGATTTAGAAAGACTAACCACAGCTC **Exon 16**
CCCTGAGGCCCAGGCTAAGCAGACAGGCAGAGGACTGGAGCCACCCGTGCCTTCCCGGTCAGAATCTTTTTCCAATGGCAACTCAGAGTCTGTG **(M3)**
CACCCTGCCCTGCAGAGACCAGCGGAGCCACAGGTAGCAGCAGCAGCCTCGCTGTGCTGAAGACTTGTAGAGTGGCTCACTGTGCCGCCCGCGT
GTAGCTCATTGTCTGTCTCACCGAAAGGAGGCGTGCGTCCAGCGGCAGGGAGAGGAGGCTTGCGGGGGTTCTTGTCTGTTTGTTTTTGTTCAAG
GTTTTTATTGAACTCATTAGGACTGTCAGCAGCCGATACCTTTATTTCCCTCCCTCCTTCACTCCGACGTGAAGTCCCTCCTTCCTCTAAAATA
CTTTTTTTAGTTAGATTTTTAGAAAGTTGGCTTTGTGGTGTAAACAGGTTAGAAGAGAACCAGGAGCTTTTCTCCTGAACATTTTAAAGTAAGG
ACTTTACTTCTGTGGTCCCAGCCCCCTGAAGGACCCTCGGAACCTGAAGGTGGCCTTGTGGTTCCATACAGCTTCTGTTTATTTGGCGGAATGT
GTAGATTTTTCTGGGAAAAGGTTCTTTTTTTTTTTTTTTTTTTTTAACAAATTCTCAAGCCTGTCAAGATTCAAAACAGGCAGGTTATGTCCA
GTGGGTTAAATATTGATTCCCTGTAGAGGGTTAGGACAAAGTTTATGACCTTCAGTCCGTCCCACATTCTGTCTTAGGCATGCTGTCCAGAAGG
GACAGTAGGTGGCAGTCCTGTCCCAGGCGAACACTGATTCTTGCTCCTTCCCTGAATAGGGTGCTTAGGCATAACTCCATGCTGCTTTGCCTCA
CACAGATCTCTGAGCTTTCTGTCCTTGGCCCTGCTGGGACAGCTGCCTTCTCCTGGGAATTTGAATGTGCTCTGCGATAACAAACCCTCCTTGG
AGAGGCTAATTGAGAAGAGCAGATCTGAGTGTGGGGTCTATGCCGGTATAGCAGCAGAGAGCTTCAGAGAATAAGACATAGAAAAAGACGGAGG
ACTGTGAGAGCAGAGGCAGCAGGAGGGTCAGCTGTCACCTGCCGCTTGCTCAACTTGGTGCCTGTTTCCTGGTGACAAGCATATGCGGTTTGTC
CTGAAAGTTAAACCCAACCCAAGGGCAAATTTACCTCAGAGCAGCAGACCTCAGAAGCTTTTGATTCAGGAGAGGAGACAACCTACCTCTCTTC
CTCCTCTGCAGAGTACTTTTGGCCTAAAGTTTCACCTGCCAGAGGGAGACTCACATGGCTCCTAGGATCAAGCCCCACTTAGCACTATTTGGTG
ACACATCTGTTCGGTTGCTCTCCCAGTGAACACTTAAGCTGTTCTAGCAGTATGGGATTGTGCCAAAGCTAGGATCCTTTGGATCCACCTCACT
GTGAGCCACAGAGACTATGCACCAGGTCCTAGCTGCCAGTTCCACCTGCAGAAGCCAAGCAAGTCATTATGAGAACCATCAGCTAGTACGTGCT
GAGCGTGATGACTGTCGTAATGGGTGGTCATTCTCCAACAGCGAGACACCGCTTACTAAAATATTTTACAGATTTTTGAGAGAATGTTTTTAAA
GACAGATTGACTTGGCAGATTGTCATGTCTGTGAGTTCTGTTTGAAAGGCGTATTCCGACCGGTCAGCCCAAGGTATCTTGATGCTGGTCAGCC
TGGGTGTCTTGGTACTAGATATTTTGCTAAGGAAATTTTAATCCCACTTTTCTTTTTTCACAATTAGGAGTTTAAATCCAGCTAGGTCCAAAAC
ATTTAAGACTGAGAGGAAATAAGAAAATTATTGGTACATTTGAAAAGTTGGTTTTTCCTAAAGTTAGTCTGCTACAGTCCCGTATCAGGAGGAG
TTTGTGTGCCTGCACAAATGTGTCCTGTGTTTCCTCACAGTTGCAGTCTCAGACTCGGGGTTGTGAGGTGGGGGGAGGTTTAGGGTTCAGCAGT
GTCTCTGTCCGTGGTCCTCACCTCTCCGTGCTGACACGGGCATGCGTGTACATGTTCCTGTCCACTGCTGTCTACAGTGCGTGTCTGGGCACTG
TTTCCTTATGAGTGTGCCTTTGCTCTTTCTCTTGCCGCGTCATCAGCATGGAGCCCGTGTACTGCTCCATGCTTGACACTGTGCCTCCCTGATT
CACGCTCCACTTCTGCTATCTCCTGTTTGGGGTTTAGTATTGACCAGGAAAGCTAGTTTGTGTGCAGAAAGCATTGAATGTTTTGTGTTTTGTT
TTCTTGTAAGGTACAGTGGTCCCACCTGGCATCTCTCAAGAACAATGTCTCCCCTGTCTCGAGATCCCATTCCTTCAGTGACCCTTCTCCCAAA **Exon 17**
TTCGCACACCACCATCTCCGCTCTCAGGACCCATGTCCACCTTCCCGCAGTGAGGGGCTCAGTCAGAGCTCTGACTCTAAGTCGGAGGTACCTG **(M4)**
AGCCCACCCAAAAGGCTTGGTCTAGATCAGACAGTGACGAGGTGCCTCCAAGGGTAAGGACAGACAGACAGACCGCGCTGTTTTCTCTTTATTT
AAACTTACTGTTTGCTATGATGGTGGGAACAGTGACTCCAACAGGGTTAAAGGGCAAGAAGTCTCAAATGACCTCTCACAGAGGGAAACCGTTC
CCCATCTCAGCAGTTACAGTCAGCCATTGGTCAGTTAGCTTTTTATCATTGAGTGGTTTTAAAACATCACTTGGTAAAAGGTATTTAAGAGAGA
AATGTTGAACGGCATGCTTCTAAGTAAGTAGAATTGTAAATTCATACAGAAGCTACCCATTATTGTGGGTACCATCAAGAGTTTCAGAAAAAGT
CTTATGAGGAAAAATGAACAAGACAGTGATTTTTTTTTTTTAATGTAAAAGTCGAGCTTCTTGTACTTAATAGTACAGGAGGACTAAAAGGCAC
GTTTCAGTGTATGTTTTTCCTCCACCTGGAATTCGCTTCCTCTGAATATTTCCTCGCTTTCTTTGCCAGTCTTTACTTCTTGGCTGATCTATTT
GTAGACTGTTGTTGGGTTTCTTTTGAGCTTTTTCTCGTGATTCTCACGTATTACCTCAGCGAAGCGCTGTTGTGTGTTCTCTGCCACCAGCTTC
AGTCCAAATGTGACTTCAACAAACTGTAGAGATGAGCCAGGCTCTTTGGATCGTGCAGGAGCAGGGGAGGCTTGGGAGAGGGGGAAGCTTCCTG
GCGGGCGTTCTGGCTGAGGAGGGCTGTGTTATGAGGAGTGCCGTGATGAGAAGAGTTTTGTCGTTGTTCTTCTGTTGTCTGTCTTAACTGCTGT
GTTTCCTACCTAAAGGTTCCTGTGAGAACGACATCTCGTTCCCCTGTCCTGTCCCGTCGAGATTCTCCACTGCAGGGCAGTGGGCAGCAAAATA **Exon 18**
GCCAAGCAGGACAGAGAAATTCCACCAGGTAAAAGAAAAATGTCAGGAAATGTTGTGCCATGGGTTGCCGGTTTTTAGAGTCATTTTTGTCTGT
TTTCATAAATGCGCTCCTCCAGCACTGAGGTCCTAGACAAACATGATCTGTTTATACACACACACACACACACACACACACACACACACACACA
CACACACACACACGAGCAGCAAATAGATGAGTAGTGAAAAAGCATATTTGAATGTTGGTGTGTATTGTAGTAATTTTGGAATGAAAACCACCTT
TCCCTGTAAAGGTAGAGAAGGGAGGATTGAGGACAGGTTCTAGGAAATGTAATTTGAGAGTTTTTAGTCTCGAAGAAATCGTGAGGGTTTAGAC
TGAGTAAAAGGAAATGATAGCCACGTCGTTGTGACCTAACAGCCTGCAGCAGACTGCCACGCCAGTCAAGTGACCTCACTGCTGAGGTTCAGGG
TAGCTCCGTCTGATGCAGTAGCTGTTAGTCAATGAGGCTAAGTCGGAATTTTAATCGCAGTGAAAGAAGCTTTACCTTTATTTGGTGGTGTCAG
CTGGGTTTATGTGCCAGTGTCCCATGTAGTGATCGGCTCTGAAGTTTGATCAGGTGTGGTGTGTGCTGCGTGTACACTCACTGAAAATTTGTCA
TCGTTGCCCTGCAGATGTCAGTAAGTGTTTGGTAATTTGGTCATCAGTGAACGTGTTGTGTTGGCAGTGTGGTTAGATGGATGATGGTCGAGAG
TCTACAGACAACCCTTCAATGCGATGGCCAGAGAGAACTGGCATTCTCCTATGGCAGTGTCCAGGGCCCAAATTCGTCCTAGTGATTGTGTGCT
ATGGCATTGGCCATGGCAGGAAGCCTCATTTACCAGCTCTGCTTCCTCTCAAAAGAACAGACCCTTGGCATCTAGGGTGGGAGAGCTGCCATAG
TGGAAGAGGGCAACGTAGATAGTGAAAATTTTCTGATTGAGAGTAGGGTCTGTAGACAGGACAGAAGTGGTTGTAGCCGTAAACAGTCACATCT

CACATTACACATGACATGGATTTCAGGTACTTTAGCAGGAAGGTGCTGTTCAGCGTCTTTGGGGTTTAAGATATTGAAGATGCTGACTCTGAAT
ATAGTCTTTGTAATGTGCAGTATGCAGTCTGTGCGGTGTGCACTGTGTGTAAAGTGCGCTCTGTGTACTGTGTGTGCAGTGTGCTCTGTGTGTG
CAGTGTGTTGTTTGTATGCTGTGTGCAGTGCATGCAGTGTGCTCTGTGCAGTGTGCTCTGTGTGTGCAGTGTGCTCTGTGTGTGCAGTATGGTG
TGTGCAGTGTGCTCTGTGCAGTGTACTATGTGTAGTGTGCTCTGTGTGTGCAGTGTGCTCTTTGTATGCTGTGTGCAGTGCATGCAGTGTGCTC
TGTGTGTGCAGTGTGCTCTGTGCTCTGTGTGTGCAGTGAGCTCTGTGTGTGCAGTGTGCTCTGTGTGTGCAGTGTGCTCTGTGTGTGCAGTGTG
CTCTGTGTGTGCAGTGTGCTCTGTGCAGTGTGCTCTGTGTGCAGTGTGCTCTTTGTATGCTGTGTGCAGTGCATGCAGTGTGCTCTTTGTGTGC
TCTGTGTGTGCAGTGTGCTCTGTGTGTGCAGTGAGCTCTGTGTGTGCAGTGTGCTCTGTGTGTGCTGTGTGCAGTGTGTGCAGTGTACTCTGCA
GTATGCTGTGTGTGTGCAGAATGCCTTGTGCACAGTGTGCTCTTTGTGTGCTGTGTGCAGTGCATGCAGTGTGTTCTGTATGTGCAGTGTGCTC
TGTGTGTGCAGTGTGCTCTGTGTGTGCTGTGTGCAGTGCATGTAGTGTGCTTTGTGCAGTGTGCTGTTTCTGTGGTGTGCTCTATGCTCAGTGT
ACTCTGTGCAGCATGCTCTGTGTATGCAGTGCACTGTGTGTGCTATGTGCAGTGCACTATGTGTGCAGTGTGTTGTTTGTGTGCTGTGTGTGCA
GTGCACTCTGTATGCAGAGTGCAGTGTGCTGTGTTAGCTACTGTTGCAGACTTTTCTGAGCCCCCACAATCTACATCAGTGCATATCTCTGACA
TAGTATGGTGGGATGACACCGTTCTCTCCTCTTCCAACAGCAGTATTGAGCCCCGGCTTCTATGGGAGAGAGTGGAAAAGCTGGTCCCCAGGCC **Exon 19**
AGGCAGTGGCAGCTCTTCAGGGTCCAGCAACTCAGGATCCCAGCCTGGCTCCCATCCTGGGTCTCAGAGTGGCTCTGGTGAACGCTTCAGAGTG **(M5)**
AGATGTACGCTATCTCTCCTACATCCTTTGTCTGGGAACAGCATGGTTGTAATTTGTTATTAGAAGGCCTCCATGTGTTTTAAGGAGGCCTGGG
TTTGCATATATTTTACAAAATGATGCTTTGACATAGATTGCTAAGGATATAAGCAGTCCCGATATGTTTCTCCAAAAGCTGCATTTCTAGTGCT
TCACACCGCAAGCACTTCACTGTTGAGCGTAACTAGCTAAATACCTCAGCGGGATTTGAACACATTTCTTCCGTTTTGAACTATGAGGCTGTGC
TGTAGTATTACTGGTAACAGTAGTTGAAAAGTCACTTTGCAGTGTTCTTCTCAGTACTTGGGAGCATGTATTAGGCAGACGTGGGGTTTTAAGT
AAGGCTGCCAGTGTGTCCCTTGGTTCTGTTTTGGCCCAGAATTCAGACTCTATGGAGGGGGTGGGGGTAGGGGTCAACTGAAGTCAGCAGGGAG
AAACCGGAAGCTAGATTGATGGGACGGTGGCATGGACAGTTTGTGGCATGCATTCATCTATGTGTTTCTGTTTTTTTGTTTGTTTTTTTTTTTT
TGTCTCTTTTGAACTTAACAGCATCATCCAAGTCTGAAGGTTCTCCATCTCAGCGCCTTGAAAATGCAGCCAAAAAACCTGAAGATAAGAAAGA **Exon 20**
AGTATTTCGACCTCTCAAACCTGCTGTAAGGATTGTATAAAATCAATTATTCTATTTTAAGCTCTAGTGAAACCTTTGGGTTAAGAATATGGGT
AAAGGGCTGGGGTGCGTGGTTGCATGTGGGAGATCCCAAGCTAGAGGCCTGCAGTGCTTTGAGTTTTGCATGACCCTTCCCTGGGTGAGGAAAT
AGAAGTGTTGTCTGATACAGGATGCCTTTGTGGCTTCACCCTTAGGCATCTCAGATCCGAAGTCATTAGTAGGGCAGCTTTTGTTCTACTTCAG
CCTTATACCAATTGTCCAGTCAGCTTTTCAAAGAAGTGCAGAAAAAAATATAATTTTATACATTGGCAGATTTTAAGGAAACAAATACTTTATA
CTGAGTAAAAGCCAAAAGCTCCAGCACGCACACGCGCACACACACACACACACACACACACACACACACACACACACACGCATGCATGCACGCA
CAATCATTGCTTGGCATTGACCTTATCCTTGGTAAGTTTGGCATTTGCAGTACTGGCTTTCAGAATAAATGTTTGCAAATTGGCTTCCCCAACC
CCTGCAGTGGCTTGAGTGCTTGCTGCCTTTTGGTCTCTGGAATGATGGCTATGACTGCTCCTCCTGCTTCCCCTCTCCTTGTCATAGTAACTAA
TTATGAGACTTGGGTACTTGTTGGGTTTTAAGCCTATTGAAGTTTTAGTTTCCTCTTTAACTGATTTCTGGATGCCCCCTCCCCCTTCTTTCTG
CTGCCTGCTCCCTTTTCTGCATCCTTCGTCAATGATGGCCTCCCTCTCCACCTCCTTGGAATCGCCGTGTCCTGAAGGGAGAAGTGGTAAGCCT **Exon 21**
GCTTCCTGCCCCCCCAAGGCTAGCTTCAGCAGTGCCCTCTCTGCCTTCTGCTTGCCTTTAATGATCACCAGCGGCCTTAGCAGATGAACCAAGC **(M6)**
TCTGGATGGTTATTGTCAAACCTACCTCTCTAGAACAACTGTTCTGAAGTTGCTCCTTTGCCACAGATAATTACCAGGACATAGTGGGCTCAAG
CCTGCTCCCCGATAGGTCCAGAGGCTCAAGGTCAAAGATTAAAAAGGCGTGTTCTTAACTGCGTAAAAGAAGAATACTAAGTTGGAACTACTCT
TTAATTAGAAAGGGGGAAACAGTCATGTAGGTGGTTTTTAAAAATACTATTGATTTTTAAATAAAACTCCTAAGTAGAAAGGGGGATTTGGATT
TAAATAGGAGGGGCAAGCAATCAGTCTAGGAAATTAGATTTAGAGAATTGATAAGAATCTTAAAGTGTCTTGGCACAGTAAAGAAAAGCCAGTT
TTTAGTAAACTTTTCTTTGAGGTTCACAGTCATTGTCTGTATAACTGGGAATGTGACTAAGTCTGGAGAAAAGCCAGTGGGAGCTGCCGTGGCA
GATGAGATTTTCCCCCCTGCAGATGCCCTCAGCTCAGACAGATGGTAGTTACTAGTAGGGAAGTAAGGGTGAGCATGTGCTAGCACTGTGCTGT
CTGGTCAGCCTGGGACTCTTCGTGAGCTGTGGAGGGGAAAAGCCCAGATACGTCAGTTCAGGCCCGTGTAACTAGAGGCCAGTGATCTGAGTTC
TGAGTAATCCTCTTAGAAGACGGTGGTGAAGAGCACTGACTCTTCTTCCAGAGGTCCTGAGTTCAGGTCGCAGCAACCACCTGGTGACTCGCAA
CCATCTGTAATGGACTCCTATGCCCTTTTCAGGTGTGTCTGAAGACAGCCACAGTGTACTCATATAAATAAGTAAAATAAATAAATCTTAAAAA
CAAAAAAAAACTATCTTTAAAAAAAAAAAAAAGACATGATGTTATGTAGTCACGTCTTTGCAGCAAAAGCCTCTTACTGTAGACTTATGAGAGCA
GACTGCTGTCAGCTCATCTTGACACACTACTGGACAGTGGGATTCAAATTGTCTGTTACACCTACCCTGTTTCTCTGTCAAAGCTGTACAGACT
CCCACATATGAAAAAACAAACCTCCAGGAATCCTCCTCTGACGCTGACCTTGGTTACTGACTGGCAGTTGGCCTGGAAGGCTCGTTACCTTTTT
TCTCCCGCCCCTCCCCCTTTCCTAACTTCCTGTTTCTTCCTGTATCCTAGGACTTGACTGCGTTGGCCAAAGAGCTTCGAGCAGTGGAAGATGT **Exon 22**
GCGGCCACCTCACAAAGTGACAGACTATTCCTCCTCCAGTGAGGAGTCTGGGACCACGGATGAGGAGGAGGAGGATGTGGAACAGGAGGGGGCT
GATGATTCCACCTCGGGGCCAGAGGACACCAGAGCAGCGTAAGTGCTTGCTGGGCTCGAAGATAAGAGTCTTCCCACTAGGCCTTCTCTCTGCT
GCAGACTTCGGCCCTCACCCTGTGGCAGAAGTCTGATGGAATGTTCATGAACTCAGTGACAGCTTTTACACACACGCACACACACACACGCGCG
CGCGCGCGCACACACACACACTACACATGCCCCGTACACACCATACACAACCACAGACATACAGACACACACACATACCACACCACGCACACAC
ATTACACACACCTCCATACACGCCACACACCACCCAGTACATGTGCCCCATATACACCACACACCAACGCCACACCACATAGACAGCCACATAT
ACACAGCCACATACATACAGATATACCACTTACATACACACCTCACAGTATCTCCACATGTCTCCTTTCCTTTTATTTTAAGGGTTCAGTAAAA
GGAACGTTTCAGTGTGGTGTGTCTTAATTAGACATAAGTATTCGTTCTAATTAATCTTGTGCTTAAAACAAAGTGAACAGCAAGGAACGTACTG
ACTGTCCTACAGGAAACTTAGGCCTGAGGCTTCCTCTCACCTTAAGCTTTGAGAGGGGTCACGTGGCTTGGCTTGGCAGCGGTGACTGGTGGAT
CTTGTCTCTTCAGGTCGTCTCTGAATCTGAGCAACGGTGAGACAGAATCTGTGAAAACAATGATTGTTCATGATGACGTAGAAAGTGAACCAGC **Exon 23**
CATGACCCCATCCAAGGAGGGCACCCTAATCGTCCGCCAGGTACCGAGCCTCTTCTTTCTATTGTCAGAGGCTGGGCTTCTCTTATGGTCATTA
ACCCACCTGCTCATCCACTCACTCGTGCTATTTCTACATCATCATCGTTTTCATGTTAACACGACTCTGAGGGCAGCGTGTCCTGGGCTAGGGA
GGTAGGCGTGTGCTGCATGTCACCATGTCACGAAGGTAGTCTCCCACCCACATTGCTGTCCCTGCATGCCTATGGCAGCCTCATCCGAGCACCA
TGCTGAGTCTCACAGTCTGTGTTGGAGTGGTAGAGAAGCCACAGGGATCCTCTGAAGGCTCACAGAGGGAAGGTACAGACCTGGAGCCTCTAAC
AGGGCCACCCTAAGGCCCTGCTGGGGCATCTAGCCCTGTGTCTGCAGCCTTGAGGGTGAGCCAATCTACTGTGGTCCAAAACAGTCATCTCTGT
TTGTGACGCAAGATGGATTCTGTTTGGACCATGATATTTTATAACTGTCTGTTCATTCTTAGCTTAGGTTTCGTCCAAGTGACAGGCGCCTCAC
TGAAGCAGGACAGGGCACTCTCTAGGCTTTGGGTTTTGCCCTGGGTGTTTCGTTTCACAGCAGTGTATTTGGATGGAAAGTAGGGAGAATCTAA
GATCAGGCTTCAGAGACACCACAGGGTACTCTCCACAGGTGGCTCAGACAGAGAAAACAAGGGGGTGGCCCTTCACCAGATGTTACTGATGCTA
ATGAACCAGGTGAAATGCTTGCTGCTTGCATTGGGTGGTCCCCAGTGAGGTACTCTTGAGTTTGGCAAGGAAGCTCGAGCAATTCTGGCTAATT
CCCTTGGATTTTCCTTGGTAATTTAACACAGTTTGAAGAGTCACACAGCCCCTTGAACAAAGCAGTTATCAGCAGGGTTATCAACGTCAGCAGC
GCCTGAGCGAGACCAGTATGCCTGTTTTTTCTACAGAGTACAGTTGACCAAAAGCGTGCCAGCCATCATGAGAGCAATGGCTTTGCGGGTCGCA **Exon 24**
TTCACCTCTTGCCAGATCTCTTACAGCAAAGCCATTCCTCCTCCACTTCCTCCACCTCTTCCTCCCCATCCTCCAGCCAGCCGACACCCACCAT
GTCCCCACAGACACCCCAGGACAAGCTCACTGCTAATGAGGTATGTCTCACACCACAGCTGGCTGCTTTCCTGGGGTTAGACCAGCACTGCCTA
GGAGCTAGTTTGCAAAGAAGCCCTTCCCAGAAGCCCTCCCCCCTTCCCTGCTCCCTGCTCTCCTGTCATCTGCCCCTCCCCAGTCCCAAGTAAC
ATGTTCAGATCTGCGTACAGATCCTTAACCCTAGAGGGACCGAGCCCCCGAGCTTGTCAGTTCACTGTTTCCTTTCTAGATAGTAGAGTTGCAT
GGCTTGCTTTATTGTTTATTTTTTTAAGTAAATAAAATGATTGCTGTGTTTTCAGATCCATTCCGTATTTAGCATGAAATCCTTGTAGTCATTT
GTGGAATCTGCCTCTTCATTCCTTTTATCCAGTGTATAAGATGCAGGAAAGCGTGACTATATAGACTTGGCCTTCCCTACACAAACTTCTTGTC
TTTCGAGAGAGTCCTGCAAATAGGCGCCCATTGTTACTGGTTACTGGTTTCCTTTGGTTATTTGGGGAGGGGGTTGGTTCTTGGTTGAGGATGA
CTTTGGTTTTCACCCACCCTTTTAAAGGGATCTGTTTCCTTGACACCCACACATACTCAGGGTCTAAGAAGTCAAGTGCCCCTCAGGGAAAAAG
TAGAAAACTATGATGCCAAGCAAGGACAAAGGCCTGAGCTTCCTTTTAATTTAAAGTTTTGTAGAGGAAGTCCTGTGAGGGCATGGCATGATTG
TACTGCTCAGTCTGGGTCATAGATGGGAGAGTCTTGGTTCTGTAAGTAGGTCATGCCCGAGTGCAGTGACAGAGCCCACCTTGAACTCCTGCTT
AATTAAGCAAGTATTTGATCTCACAGGTTTGGGGCCTGTTGAGTCCATTGTTTAATAATTGAGAGAGATCTAAACGGGCATTTAGTTTCAGCCA
GTCTTGTCAACATAAACACTTGATTGACTGTGGACAACAGCGATGATTTTGTTCTCTCGTTAGAAAACACAGGACAGTCTTCTCCTCCTGAGGC
CAGTATCAGAGAGCTGCTTTCTGAGCAGCCTTGACGTCAGACTGGCCGTCGTGACTCGGCCACTAATTGATAGAGCAGGGCGAGAAGCCTGTGA
GATGGCAGGTGTTGTGTCACGTGGGGAAGCTTAGGGTGGCATGGGCTGTAGGACAGTGACTTTTAGGTGCTCCACAGTGGAAGTGAGTAGAGGA
AAAGGCGCTTGATCGGTGTCTTCTGTCCTGAGACCTGTGCTCGCACGCCTTTCCTTAGTGACACCACACGCTTCCTCTTACCATAGCCTTACCT
CACTAAACTGGCAAGTGTTTCTGTTTGGTTTTAACCATCATTACATCTCCACTTAACAACTGTTTCACTTCCTCTGTGAGCTTTTTGTCTGTTT
TCTTTTTTGGTTTTAAGTTATGTTATTAACACCAGTGCTCCTAAAAGGCCAGAAAGCCATGTCTACTGTCATCTGTGTGTTTACTAATGAGCCT

TGTTGTGTGGAGTGGACTTAGTGGTGATTCTATCATAGTACAAAGTAGATCACACCAGCAGTCTGTCACAGTAGGTACATCACACCAGCAGTCT
GTCACAGTAGGTACATCACACCAGCAGCCTGTCACAGTAGGTACATCACACCAGCAGTCTGTCAGAGTAGATCACACCAGCAGCCTGTCACAGT
AGGTACATCACACTAGCAGTCTGTCAGAGTAGATCACACCAGCAGCCTGTCACAGTAGGTACATCACACCAGCAGTCTGTCAGAGTAGGTACAT
CACACCAGCAGTCTGTCACAGTAGATCACACCAGCAGTCTGTCACAGTAGGTACATCACACCAGCAGTCTGTCACAGTAGGTACATCACACCAG
TAGTCTGTCAAGTAGGTAGATCACACCAATAGTCATAGTAGAGAGGAGATCACACTAATAGTCTGTCACAGTAGATAGGAGATGAAACATTTAT
TAATTTTACTTTGTAGTGTGAGAAGTCAATTTAGAAAGTAATGAATTGTTATTAAGACACTTCAGATGCTTATTTAAGAATTTTCATTTTCCTT
CAAAGTTGCGGTCATGAAGTGGTGCTTTTATCGGTAGTTTGCAGCATCACTACCCCTGGTGTGTATGTGTCTCCAGGAAGATGTGGCATTTGAA
ATGCCTGCTAGGTGTTTTGGGGATTGATGCAGGAGGAGAGGGCTTGGATTGGGAAATGGTACTCTGTGTAAGTGGATTGGAATAGTCTTTACTG
TTCACTTTTTACATGTGTCTCTTCTATTAATGAAATTGAAATTTACATTTCAGACTCAGTCCGCTAGTAGCACACTCCAGAAACACAAATCTTC **Exon 25**
CTCCTCCTTTACACCTTTTATAGACCCCAGGTTACTACAGATCTCTCCATCTAGTGGAACAACAGTTACTTCCGTTGGTAAGTACTGCGGCGTC **(M7)**
GAGGAAAGCAATTGTCAGACACAGGGCCCCCTCCCCGCGCCGCTTCCCAAGGGGCTCAGGCATGGCATGGTTGCAGGTGGCACGGTCCCTCCGC
TAGAAAGGCCATCCGCTCTCATTGCCTGGGTCTCAGTGAGGTCATGGTCAATTTCCTTTCCATGCCATTGCTGCTAGAACACCTTTTCTTGAAA
ACGTGTCAGACGCGGCTTATGAATGTTATCTGGGGCATACCGAGGTGGGAGGCTGCGGTGTCTATGTACAGAAGTTACGTTTTCACTGTACTGT
GTTTCAGTGGGATTTTCCTGTGATGGGCTGAGACCAGAAGCCATAAGGCAAGATCCTACTCGGAAAGGCTCAGTGGTCAATGTGAACCCCACGA **Exon 26**
ACACTAGGCCACAGAGTGATACCCCCGAGATTCGTAAATACAAGAAGAGATTTAACTCAGAGATCCTGTGTGCTGCCTTATGGGGTAGGTACCT
CACTGCTATCGGATGACTTGGCTTTATTCTGAAGCACAGAGTTGCCCTGCAGAGAAGCACACACTGAATGCTGTCCTTATTCAGTTGTGTTGTG
CAGCAGGACGCACGGATGTGACTGCAGTGGTGTCAGCAGTAGTCCCCTCCTCCATGTGGGGTGATGAGTGAGAGGAGGAGAGAGGAGCTGGTTC
TTGTCACGGAAAGAGCAAACCTGGATGTCTGCCTTCACCGCTTTGTCTGTGACTGACCAGTGTCCAGATGAGGCTCTTAGAAGCAATCTGCACA
GGGCCGGCTTGTCTGTGTGCTCAGGGCAGCAGCAGGGCTTTGCTGCACGCTCGGTTCTGCCTTCTCCAATTCAAACTAGCCTTTCTGCCAACTC
CCTCCCTGGAATCTAGCCCACAGTTGATCTGCTGTTAAAGTACCCTGCTTCTACCCAGAACCTCTTACTTCCTGGACCTGCACATGCAGATGAC
ATGACATGAGACACTGCACATGCAGATGGCATAAAGTCACACACTATCCACAGTCCTGCTCTCTGAGCCAACACATCCCCTGCAGCAATAAATC
AGGTCCCCTCCCATCCCCATCTCATCCCCTATTACTCCTTTCTGTCCGTTTCAGAAGTAGACACAAGGGCTTGCCTTTTATCTTTTGAACCTTA
CACCATTCCCAGTCTCACCCATGTAGCCTGCCCCTTCTTCCCCTCCTCCCCCTCCCCCCTCCTCCCCCTCCCCCTCATCCTCCCCCTCCTCCTC
TTCCTCTTCTTCCTCTTCCTCCTCCTCCTCCTCTCTCTCTCTCTGGCGCGCTCTCTCTTTCTCTCTCTCTGTCTCTCTCTCTGTCTCTGTC
TCTCTCGCCTGCTTAATCCCCTGCTCTGCCCTGGGCAGGAGCCTTTGAGAGGCTGCCATGTTCACAGCAGGCCCACCTGCTCCCTCAGGGTCAC
AGTTCCTGCCTTTGCCATTACTGCTTTGCTCTCATGATCCTGCAGTAAGGACCTTGTTGACCATCTGGATCAGTGGTTCTCCACCTTTTGGGGG
GTCAAATGAGTCTTTCACAGGGGTTGCCATCAGAAAACATAGATATTTATGTTACAAATCATAACAGTGCAAAGTGAAAGTCATGAAGTAGCAA
TGGAAATAATGTCATGGTTGGAGGTCACCCTGGCACAAGAAGCTGTATTAAACGGTCGAAGCTTTAGGAAGGTTGGGAACCACGGGTATAGATA
AAAATAGAGTTAGCTATTTAGAGGATTATGGGCACCGCACAGCCGGAAGCTGGCACAGAAGTGTGCCTAGCACACTGCTGTTACTCAGTAGACG
TCACTTGGCTGCTGTTCCAGAAAGCAGGCTGTCATTTAATCACATCGAAAGATTTTAATGTTGTTCGCATGTTACATTAGGATAGAGATAGTGT
TAATATGGCAGTTATGTTGAAATTGGTCATCATTTTGCCCAACAAAGTTACCTTGGCAACAGTCTTAGTGCTCCATCATGTTCCCCTAACATCC
TGGCAAGCCCGGGATGCAGACTCCGTTTCTGCACTGCATCCAGTGAGATGATCTCCCCGAGGGCCTTTGCTTAGCCGCTTCCTTACTGGCACAA
GGCCTGACAGCTGGCTAAGGAGGCCTTGTCTGTACTTACCAAGTAGCGTGTTTGGCTTGAGACACAGAAGGACTAGGCTTTTATGTTGATCTCT
TTATATGGATAGTGTTCTTGTAGATATCCCATTAACCTATGAGTCGGGTAAACTCACAGCATTGACTTACATGTGGCCTTACAGCTTCATAAAC
TTACAACAAAACTTGTTTAAAAACAAATTCTTTAAGAGTTACACAAATCTGACTTAATCTTGGGTAAAGGTGCCCAATTCTAGATACTCTTAGT
TTTCTAAGCTGTTTTAGACTACCACCCTTCAACAGACTGTCTTGACTCTGTCTGTGACTCCCCTTTTCACAAGATCATTGACCTAGCAGGTACT
TTGAAAGGACACAAATACTTCATCGCTTGTCCAGCTTAGAGAATTGGCGTGAATGTGTTTAGTTCCCTGAGAGCATAAGTCGATGCTACATGTT
AGAAGATGCTTTTAAAGGCGAAGGTTCTACACCCAAGAAGCAAGAGCATTTGGATAAATATATGAATTTAAAGGGAGAGTCTAGTGCTTCCAAA
AATAAGGATGATTGGATATTAACAGGTTTTTAATGGGTGCGGCCAACTGGCTTGAGAGCTGTGGGACCCCAGACGCTAGCAAAGGACTTGGGCA
CTGCATCTTAGGTGCAGGTAAAGTTGCAGCGGGACTCATTGTCTGTGTAAAACAACGGGGATCAGTTTGATGTGCACCTGTGAGACACGAGGTT
ATCTGGACAGACTGAGTGAAGGCCTGTGTCTGTCAGCTGTCACATGATCAGAACGCATTGAATATCAGAGCCAGAGGAGGGAGGGAAGGGGCAG
GCAGAAGATGTTAGTTACCTTCAGTGGGAATCCTGGTTTCAGTAGACATCTGTAGTGTGTGGAAGAGCAGTAAGTTCAGCATGGCCGTGGGAGC
TGCTGCCCTGGCACAGATCGCCATGCCTTTGTTTGATCAGTGCGAGGTGGTAGCAACAGTGTCCATGGAAGAAGCAGAGAGAGATGGAAACAGG
AGACGTCCTGACAGAGTAAGCAGGCCACTGGTGTGGTCAGCAGTGCGTCACAGGGAAGGCAGCTCCCAGGAAGTGGGGGACAATGGTGCAGTTG
TTTCTCGGTGGTGGGAACTGATTGAGACTATCTCATAAGCCAGTGAAGATTACAGGGGCTTGTTCTCTCTGTAAGTCGTCCTACAAGGTTGCCA
ATTGTGAGGACGTTGTAGCAGATGGACCCAAATCTCTAAAACCCCAGAAATAGTGAGTTTACAACTGGGAGCTCAGTTCCCTGGGAGCAGGTGA
GGTGGTGAGGTCTCCTTATTGCTTCCACATTTATGTAATCTAGATGTAACTGTCCCTTCTGTGTGGGAGGGGTCGTAGCTTTAACTGTTCTGAG
CATTCCTTCAGGAACCACGGACACCGTCATCTTTCAAGTAGAGAATGTTTCTCTGTTCTACGGTACAGCTCAGTGCAATCACAACCTAAAATGT
AGGCCTTTCCATGTCCTTGAACAGGAGTGAATTTGCTAGTTGGCACAGAGAGTGGCCTGATGCTCTTGGACAGAAGTGGCCAAGGGAAAGTGTA **Exon 27**
CCCTCTGATCAGCCGGAGACGGTTCCAGCAAATGGATGTACTTGAGGGCCTGAATGTCCTGGTGACAATATCTGGTGAGTGTTCTGTAAAGCAG
AATGGATGGCCTTGGCCACGGTGCACCAGGCCCTACATACTGAGCTCAGCTCATTCTCGATGCTGGCTGAGACAGCAGAGTTGAACGTCTTCAT
GATGTGAAGATGCGCACAGCTGTGGCACTCCCTGACACCCGTCGTGACCTCTGTTGTACTGTTGTACACTGGTGGCACATTGTCTTTTATTTAC
TGATAATAATACATTACTATTACCCACAGCATGTCGTCACTGGCGTTACTGTGGAGTGCATTTGCTTGGTTCTCACACATACACTGATGCGTAT
ATTTCGGTCTGTCTAGTTTCGGTACCCTAAAAAGCGCCATTTGTACTTCCTCCTGAGGCCCTGACGATAGCTGACCAGCTGTTTTTGATGACTT
TACTAGTCTAGAATGTCCTATCATTGGACACGTAGATATAAGTTTGCGTGACTGAGTATATATAGGTCATGTCGTATACGGGCTAACTCCTTTG
GCAGTGCACATTTAAGTTAAGGTTGCTTCCTGTCTTTTGTGGTTCAGTATCTCGTTCTTTGATACCGCCAAGTGCTGCACAGACAGATGTGTCT
CTGGTTATCCGTTCATTGGAAGACAGCTTGGTGCTTTCAGATAGGAAGGTTTAAGGAAAGCTGCTAGTAGCTGTGCAACTTTTTGCTTATACAC
AAGCTTTCATTTGTGTGTTTAGTAAAAGAAAGATGGAAGGAAGGAAAGAAAGAAAAAGAAAAAGAAACAAAGTAACAGCTGACAGAAGCGTGAG
CAGAAGTCTGTTGGTGCAGAGAGCTGCTGCGCTGTCCTACAGAACGGGTCCATTGTCTCCCAGCAGAGAGCTGGGGAAGGACTGTTTCTGCACC
ACAACCCCGATGTCTGAGACTGTGTTGGACGTTTTCCATCTTGATAGGTTAACAGATGTCTTGTGTGAATATGGAGTCCCCTTGTATGTGAATG
CTCCATGCACCCTTGTGGTTAAGTCTCCTCTTTCTCCCCATGGAAGGATCTTCTTAGACCTTTGTTCCTTATTGCACTTGAGTTGCTTTTTCTC
CCTCTTGTTGAATTTCTTTGTATATTTTAGCAATCTTTTCCTGCATGTGCCCTGAAAGTGCTTTTTCCCTGCCTCTGGCTTCTCTGTTCGTTCT
CCATGTCTTGCAACGCAGACATTATAAAGTTTAGTGAAACCTCACACTTTGTGCTTGCTTTGTTTTGTACTCACTTTTGTGCTTAATGCTTTTG
GTGTTTTACCCAGAAAGTCACTGCCAAATCCAAGATCCATCCAGATATTTTCATGTGCTTTCTTCTACAAGTTTGGTAGCTTGGGTTTTCACAT
TTAAGTCTGTGACTCATTTTTAATTTATTTTTGTCTAAGGTTTTAAGGTTTACAAGTAGATTCCCTTTCATTCACAATATTGTTTATTTCAGAG
AAGACTTCCTTTTGCTATTTGTTTACTGTGTTCATTTCTGTGCCTCTGTTTGTACTGTGTATGCATGTGTTTGTTCACTGTGTTTATGTGCATC
TCCCTGTACACTGCGATTGTGTCCCTTTGTTTATACTGTGTGTGCACCTGTTTGCTCACTGTGTTTGTGTGCATCTGTTTATACTGTGTGTGCA
CCTGTTTGCTCACTGTGTTTGTGTGCATCTGTTTATACTGTGTGTGCACCTGTTTGCTCACTGTGTTTGTGTGCATCTGTTTATACTGTGTGTG
CACCTGTTTGCTCACTGTGTTTGTGTGCATCTGTTTCTGCCCCTTTTACTCCACTAATTGATTTGCTCGTTCTTTTGCTGATAACCACAGGGTC
TTGGTTACTATATTGAATATATAGTAAATCTTGAAATCAAGAACTGTCTAATAACTTTGGATTTCATTTCAGTATTTATTGGTACACACAGATA
ATTTGGCTATGGTTTCGGCTAGAATTGAGTTGAATCTGTTGTTCAAGTTAGGAAGAACTGACGGTTCTATCCATGAACATAGTACTTCTCCACT
TAATTCTTTAACATCTTTGACAAGAATTTTCTAACTGTCCTCATAGATATTTTAAACAGCTGTCTTGTGAGATCCATCTATTTCATTTCTTCAA
TGTTAATGTGAATAGTATTTTTATTTGTTTTTAATTTTTAATTGTTGGTATATAGGAAAGTAATTGACTCTTGTATAATGGGCTTGCTAGCTAA
AATCATGGCTATAATTGTTTCAAGAAGTTCCCATGCCTACTGCTTTTGTTTAGGGTTTCGTTTTTTTCTTGGATACTGTTGTCATTAATAATAA
TGTTGACTTTTGACCACTCGTGTAAAATACTTATATTTATCTTGTTAGGCAAAAAGGATAAGTTACGCGTCTACTATTTATCCTGGTTAAGAAA **Exon 28**
TAAAATACTTCATAATGATCCGGAAGTTGAGAAGAAGCAGGGGTGGACCACCGTGGGCGACCTGGAAGGGTGCGTGCACTATAAAGTCGGTGAG
TGCTCAGTGGCGCCGTGCTCCCGCTGCTCTTCCGAGTTCCGTCAGTCGTCAGTCCGGTTTAGGTTACTGGACTCTGAATGGTGAGGTGTCCTGT
GACCGTGACCTGCAGGTCTCTTCCCTTCCTGACCTTCCAGGCTTGTAGTTAGCATATGCTGAGAGGTCCTTCACAGTCACTAGAAGAACGTTTG
AGACTTCAACTGCTGTCACGGCTCGTTATCTTTAATGAAGTACATCGTGCAGAGCTGCCGTGCCCTTATTTATAAATGACCATGGCTGGCCTCT
TAGCAACTGTTTTGTGATTGGTATTTTTCTTCTTTGGAACATTGAGTGATTCTCACAAAGTCTTTTCACAAAACCCACGCCAGGTTTCATTGCA

TAAGAGAAAGTAAGTGCTTGTTAGAAATGATGAGAAAGAATGGACCATAACATAGTTAGAATTTTAAGGAACCAGCAGTGTAGTTTTATAGAAG
TGCACTGGTTACAGTCGAGGAGTCCATTGATTGCTAATTTGTGCTTTCAGATCTTACTAACATACCAGCCATAGGCAGTTATTCTGTCCACTTC
CCCAGGATATTGTAATCGGTGACATTGTCTTCTCTGTCTATTGTGGCTACTCATTGGTAAACAATTATTACAGTTAGCATCTCACTATGTGAAA
TGATTCTGAGTTCCCACCAAGACCAGGAGACTGAAATACAGTTAATTAAAGTTAACAGGCATTAGTTTGGTTCCCATGTGGCCCTTGAACCTCC
TTAATATTGGTAGTTAGGGTATGGTCTATAGGGATTATCAGTGATAGCTTTAGTATGTTTGGTTTATAGAGATTAGCAGTGCCCCTAGAGTGAT
GTCCATCATTTCAGAACAGTCTATTAACAAAGCTGCACCCTTGCACCTAGGCAAGCAGCAGTATGATGTTCAGAGGTCCATGTAGGTGGTCTAC
AGAGCAGACCGGAACATCATACTAGGGAGCAGGAAGGGCTGCGAAAGATCATGTATGTTCTGTGAGTCACACATGACTTATAACTTGCTTCTAT
CATATTCCTCTGTCTGTGTGTGAGCCCTCCAGCCCTCAGGGTAGCTGCCTGGCGTAGCCTTTGGTAATAAAATCCTTGAAATCTAAGACATTAT
TAGGATTATCAGCAAAACTATTCACTCTCAGTTTAGAATTGATTTAGGAAATTGAATCACACACTGAAGAATTGGGATAGCTACGTAAGTGGCT
GAAGCTCCCTGTATTTCTTACCCCAGCTCCTAATTTGTTGGATGAGCAATAGAAGGTATATTTATAAAGTTTGCAAATAACAACTAAAGGAGTT
TATGGCTCCATGTTAATACTGATAGTATTTCTCTTGCTTTTTATGTTTGTTTCAGTAAAATATGAAAGAATCAAGTTTCTGGTAATCGCTTTGA **Exon 29**
AGAGTTCTGTGGAAGTCTATGCATGGGCACCGAAGCCATATCACAAATTTATGGCCTTTAAGGTAAAACCGCCAGTTTTAAATTGGCTTAAAGG
TCTGAACAGTTGACATAGAGCTATGCATTGACATGACATACTTGTCAGATCAGCTGCTCTAACATAGTATAATAGGCTTGCGCAACTATTTCCT
AGGTTCTCAGATGGTCAGTTTGCCAAAGTGGTACTTGCACCGTCTCTTAGAAAGTAGGCGGTCTCTGGAAGACACAGATGTGTCGCAGATGGAG
ACTCGAGCTTAAAGGGCTCAGTGAAAGGCAGTCGTGAAATGCATGTGATCGGTGCAGTGCAATTTAAAGACTAAGGAATAACCTGGTTATGATA
GTACAAGCCAGAGAGGCTGAGGTAGGAGGGTTGTTGTGGGGTCAAGGCCAGCCAGGGACAGCTAGCAAAACAAATAAATCAAACTAAGTAAATC
ACAGAGGTTTTTAATGATATAAGCAATACATATTAAATAGAAGGGAGTTAAAACATGTATAATATGGATCTGTTTTTATATAAATTTGAAAAAC
TCCCTTTAACAGCATCTTGGTATTTACAGTGAGGAGACTTTTGCTACACTCGGGACTGTTGTGGTTGTAAGTTCACAAGCACTCATAATTGGTC
TCCTATTCACTCCGGTGTGCGGCGTGAAAGGTCGCTTATGAAGCAGTGCTGGTAACTGTCATAGTCTGTGCTTCCACACTGCCAAAGTGCAGAG
AAAGAACTTAACAGTCATTGTTCTGTGTGTGTTTTTAGTAATTTATTTTAGGGATTACCCTAAATCCAGTGTATAAAATTCTTAATTAAAATTT
CTGCTGTTTTGGCATTAGATAGAAATAATAATTACCTCACAGATAATTATTAGAACACAGAGCTCTAACAATAGTCGGGTAACATTTTAGCAGA
GGGTAAGAAACAAATTACATCATCTAAAATGTGCTTAAAACAATTTTGATATAGTTTATTGAAAGAGTAAGTTTCAATTACTTGTGAAATTTGA
ATTAAGGGTACTTCCTGCTTGGTTCCCCCATGGAGTGGAGTCAAACTCACGGTTATCATTTTTGTAAGTCCTAGTATTACTGAGAAATTATAAA
GTCACTCACATTATCTTCCGGAGTCTGGTCTTAGTATTCGTATGTGGTAGCCCTCACTGACGTGAAGAATTAAGGCGCTCCAAGTCACGATAGC
CTGAGTCTAGTCTCACAGCTAAAGGCTATAGCTCTCGCAGTATCAACTCTGACAGAGAAAGTCTCAGTGCCATGACCCTACAGCAGCTCACTTC
ACTGCTGTGTCTGAGTATTCGAATCCCACACTGTGCCATTCTTACTATTGTCAACTTTGCCTATGCTCTAATCAAGACACACAACTGGACAGGA
CAGAGAGCATTCTTCAGCTAGGTAGGAGGGAAGACGTGTTTTCCAGATGCTGGTTAACCATCCATGTGATAAATCAAAGCCTGCAGCTTGCTTC
TCTGATGTGTAAGTTTGTTCTCCTGCTTCTGTCTTGATTACCTCTATAGTCATTTGGAGAACTGGTACATAAGCCATTACTGGTGGATCTCACT **Exon 30**
GTCGAGGAAGGCCAGAGGTTGAAAGTGATCTATGGATCCTGTGCTGGATTCCATGCTGTTGATGTGGATTCCGGCTCAGTCTATGACATTTATC **(M8; M9)**
TACCAACACACGTAAGAAAGAACCCACACTCTATGGTTGGTTGACTGACTTCACTTTGTTTTGACTTTTCCCTTTACTCTGCTCAGTGACTAAC
ACAAGCAGGGTTTCCTCATTCCCCGTGGTGTAGGGTGAGTATTTAAATGAAGCACTGTTTTCAGTGGCTTCGGAACCATAGAAGTCTCCTTCCC
AGGCTCTGTGAAGCCCTCTGAGGACCTCTTAGCATTTGCTTTGACTAACATGGGATGGAGTTGGGACTGTTGCTGTCAGGCAGAACACCCTGGG
TTGGGAATGGTTCTCAAATGAGTCCACCATCCGGTAATGTGTCCAGGGACTCAAACACGCGCATCAGCAGAGCACGTGACCTGGAATATAACTC
TGGGTTGTATATTGATTGCATGGACTTCCCAAGATCCTTGCCGGTTTGTGGTAGGTACGGCCAGGGGTGCAGCACTACCGCATTCGCTAGACAG
ACAGACTGAGCACACTCACGGCCCTGGAGATGGCCACAGACGTGCTTCTCTCCCTGTTCTCTAGATTCAGTGTACCATCAAACCCCATGCAATC **Exon 31**
ATTATCCTCCCCAACACGGATGGAATGGAGCTTCTGGTGTGCTACGAAGATGAAGGGGTTTATGTGAACACGTACGGAAGAATCACCAAGGACG
TGGTTCTGCAGTGGGGAGAGATGCCAACATCTGTAGGTATGGTGCGCTGTGACGAGGCGTCCGTGAGAGGAGCCGCCGAGACCAGTCTGTGTGT
TTACTAATGTTGCATGTTGCTCGCACCGTCTGGCTATTCGGTGGTTACCAGCAGATGGGAGGAGGTTAGAGCAAATTCCCGGTAACCCTTGGGC
ACTTTAAATCTGCGCAAATGCTGCCTCTCCTTGGACTCTTAGGTTACCCAAAGGGCAGCCTTTGGGGTGTTTGGTTTTTTGGTTTTTGTTTTTG
ACCAGTATGCATGGCTGAAGAAAATCACAGAAAGAAATAGTGCTCTTGGGTGGCAGGGCCCTAGTCACCGACAGGCCCTGAGTGGTCTTCTGCT
TCGTGTTCAGGGACCGAGGCTGCTGCTTGACGTTGTATGGCTGTGCTTGTGTCCCCGATATGTCTGCTCACCCGTCTTCCTCCCTTCTCTCATG
GCTGTTTCTAGAAGTGGCATGAAAGACCTGAGGGAAGTTGGCTTGCTCAGTTAACTGACGCATGCCTAGAGTGACAGATCAGGCATACAGCACA
CTGCCCCCTTCTGGTCACTGCTGTAAATTGAACCCAGGGCCTCGAGAACGCGGGGAGGTGGGTTTTCAGAGATGTGCCCAGTTCTGAGGCAGAA
CTCCTGGAAAACCAAAGTCCTCACAGGCTAGGCACCCAGCTGGGCGTGAACACGAAGCATCTTGTCAAGATTCCTCCTGGTTAATGCTTTGTTT
CTCCCTCTCTAAAAGCATACATCCGGTCGAATCAGACAATGGGCTGGGGAGAGAAGGCAATAGAGATACGCTCTGTGGAAACTGGTCATTTGGA **Exon 32**
CGGTGTGTTTATGCACAAAAGGGCTCAGAGACTAAAGTTCCTGTGTGAACGTAATGACAAGGTAAGGCTTCCTCATGGCGTCTGTGCACTTGTG
GCTGCTGTTTATGGTGGCTTGTTGTGCATGGGGCAGTGACGGTGAATTCCTGGCCGTTCTGGTAGATTTCTAAAATAAATCCAGGAGACCTCTG
AATGCACACACTTGGGGTCGGGCGCCCGGAGGTGGTGAGTGGCTGCTGACTGTCCTCTCCGGTTGTGTTGCAGGTGTTCTTTGCTTCCGTCCGC
TCTGGCGGCAGCAGCCAGGTCTACTTCATGACCTTAGGCAGGACTTCCCTTCTGAGCTGGTAGAGGCAGTGTGAGCCGGGATCACTGACCTCTG **Exon 33**
GAGTCGGCAAGACCCCAAGAACTTGGAATTCCTTGCAACTGGAGCTCAGAGCTGTGCCGGTGCAGCCATGACAGCTGTCTATGCAGGCCTCTTG
TGGCGTGGCGTGTCGGGTCGGGTCCTCTCCCCTCCCTCCTACACTTTCTGCCAGTTTATTCCCATTCTCTCCTCCTCCTTTCTCAAAAA***AATAA***
AC***CA***AGGCTGCATTGCAGCTGGTGCTGTTCAGACTCTACCATCGGTGCTAGGAATGCTTGGGGATGAACACATCAGACCGGGAAGAGCGCCTTT
CCTACAGCTCCAGGATTCCTCGTCTCCGAGCCACTGTCCCGTGGGCGTCTGCTGGTTGTGACGATTGCGCCACCCGACGTTGGCGGCAGGCACA
GAGAGGTGAAAAGTTGGTGTAAGAAGCCGCTTGCTGAAGCAGAGAACAGAGTTAATATAGTAACATTAACAATGTATTTAATTGACATTTCTCT
CTTTTTGTAATGTGACAGTATGTGGACAAAGAAGAAGGTGCAGGTCTAAGAGGTTAATATTTATAAAATGTGAAAGACACAGTTACTAGGATAA
CTTTTTTTGTGGGTGGGGCTTGGGGTGGGGCAGGGCGGGTTAAAGGGTCCCATTTTGTTTCTTTGAAAGCTTTTTGGAGAGGGTTGTCCTGGCC
AAGAACTCAGTCATTTTTCTGTGTACCAAGTTTTGCCTAAATTGTGCAGATGGTTCTTAAAAAAGAAAGGAAAGGAAAAGGAAATGTGTGCGTC
TTGTTGGAGGGCCAGAAGTTTTGTTGTGTAACAGTATTAGCAAGGCCTGGGCTAAGGGTTTAATGTCCGTTAAGCCTAAAGTGCGGCAGCAGCT
CCACCACGGTCTGCATGCACCATAGGAAAGGCCAGCTTTCCTGTGGGTTCTCATTTCGTTTCGTTTTGTTTTTTGCCTACCTCATTGTTCTTAA
TGCATTGAGAGGTGACTTAGCTTAATGGTTTTGGCAGAAAACATTTAATGTTTAATTTAACCTTAATACCAAAACTATCTGATTAAAGTTTGAC
TGACTTTGTCACAAGTCTAATCAGGTACAAAAAAAAAAGGAAAAGAAAAGAGAAAGAAAGAAAGAAAGCTGTCTATGATGGTGGGAGCAGCCTC
AGCACTCAGACGCTCTGCTCTCACCAAGTTCACCATTGGAGGGTGACAGGCAGCGGAGTCACCTGTCCTGACAAGAGATCTCACTGTTCGCAGT
TACAGGCTTTGGAAGCAGGCAGGGGTCACACTAGGAGTCACACAGACTATTCATTCATGTTGCAGGAACTTTTTCTTGGGAGCGAGGGTGTGTC
CCTTTTCTTAAAAAAAAACAAAAAGCAAAAAAAACAAAAAAAACCCCGCAATGCACTAAAACTATTTTAAGAATGTAGTTACTCCTACTTCTTC
ATAGACGGCACCTCCCTGCCTGTGTTTCAGGTGTAAGACTGACGCTCTGGCTTTTCCTGTTTCTCACGTGCTCTCTTGTTTGTTCTCTCTCTCT
TTTTTTTAAAACTAATTTTTCTTTATGAATATGTCTGTGACATTTGTAATAAATGTCTTGTAATAATTTGC

Supplemental Figure S3. Alignment of MAP4K4 cloned from neonatal rat cardiomyocyte cDNA (blue type) with the predicted sequence for transcript variant X22 for rat Map4k4 (XM_008767015.2; red type). Start and stop codons are highlighted in yellow.

GGGCGACGCCCGGGGGGTGGGTGGGGGCCGAGGGAGGGCCGCCGCGCCATGGTGTGAGCCCCGTGGCCCGCGCTC
GCCGCGCTCCGCTCAGACCAGACGACACGCCGCGCCGGCGGCGGCCTGCTTGTGCGGCCCGAGCCCCGGCGCCGC
CGAGCCCCTGCCCGTCGCTCGCTCGCTCGCCTGCTCTTGGGGCGGCGACCCGGGGAAGATGGCGAACGACTCTCC
--ATGGCGAACGACTCTCC

GGCTAAAAGCTTGGTGGACATCGACCTGTCGTCCCTGCGGGACCCTGCTGGGATTTTTGAGCTGGTGGAAGTGGT
GGCTAAAAGCTTGGTGGACATCGACCTGTCGTCCCTGCGGGACCCTGCTGGGATTTTTGAGCTGGTGGAAGTGGT

TGGAAATGGCACCTATGGACAAGTCTATAAGGGTCGACATGTTAAAACGGGTCAGCTGGCGGCCATCAAGGTTAT
TGGAAATGGCACCTATGGACAAGTCTATAAGGGTCGACATGTTAAAACGGGTCAGCTGGCGGCCATCAAGGTTAT

GGATGTCACCGAGGATGAAGAGGAAGAAATCAAACTGGAGATAAATATGCTGAAGAAATATTCTCATCATAGAAA
GGATGTCACCGAGGATGAAGAGGAAGAAATCAAACTGGAGATAAATATGCTGAAGAAATATTCTCATCATAGAAA

TATTGCAACATACTATGGTGCTTTCATTAAGAAGAGCCCTCCAGGACACGATGACCAGCTCTGGCTCGTTATGGA
TATTGCAACATACTATGGTGCTTTCATTAAGAAGAGCCCTCCAGGACACGATGACCAGCTCTGGCTCGTTATGGA

GTTTTGCGGGGCTGGTTCCATCACAGACCTTGTGAAGAACACCAAAGGGAACACGCTCAAAGAAGACTGGATTGC
GTTTTGCGGGGCTGGTTCCATCACAGACCTTGTGAAGAACACCAAAGGGAACACGCTCAAAGAAGACTGGATTGC

TTACATCTCCCGGGAAATCCTCAGGGGGTTGGCCCATCTCCACATTCACCATGTCATTCACCGAGACATCAAGGG
TTACATCTCCCGGGAAATCCTCAGGGGGTTGGCCCATCTCCACATTCACCATGTCATTCACCGAGACATCAAGGG

GCAGAACGTGCTGCTGACTGAGAACGCGGAGGTGAAACTTGTTGATTTTGGTGTAAGCGCTCAGCTGGACAGGAC
GCAGAACGTGCTGCTGACTGAGAACGCGGAGGTGAAACTTGTTGATTTTGGTGTAAGCGCTCAGCTGGACAGGAC

GGTTGGACGGAGAAATACATTCATAGGCACACCATACTGGATGGCTCCGGAGGTCATCGCCTGCGACGAGAATCC
GGTTGGACGGAGAAATACATTCATAGGCACACCATACTGGATGGCTCCGGAGGTCATCGCCTGCGACGAGAATCC

AGATGCCACTTACGACTACAGAAGTGACCTCTGGTCTTGTGGCATCACGGCCATCGAGATGGCAGAAGGAGCCCC
AGATGCCACTTACGACTACAGAAGTGACCTCTGGTCTTGTGGCATCACGGCCATCGAGATGGCAGAAGGAGCCCC

CCCTCTCTGTGACATGCATCCAATGAGAGCGCTGTTTCTCATCCCCAGAAACCCTCCTCCCAGGCTGAAGTCAAA
CCCTCTCTGTGACATGCATCCAATGAGAGCGCTGTTTCTCATCCCCAGAAACCCTCCTCCCAGGCTGAAGTCAAA

AAAATGGTCAAAGAAATTTTTCAGCTTCATAGAAGGCTGCCTGGTGAAGAATTACATGCAGCGGCCCTCTACAGA
AAAATGGTCAAAGAAATTTTTCAGCTTCATAGAAGGCTGCCTGGTGAAGAATTACATGCAGCGGCCCTCTACAGA

GCAGCTTTTAAAACACCCTTTCATAAGGGATCAGCCCAATGAAAGGCAGGTTCGAATCCAGCTTAAGGATCACAT
GCAGCTTTTAAAACACCCTTTCATAAGGGATCAGCCCAATGAAAGGCAGGTTCGAATCCAGCTTAAGGATCACAT

AGACCGGACCAGGAAGAAGAGAGGCGAGAAAGATGAGACGGAGTACGAGTACAGTGGCAGTGAGGAGGAGGAGGA
AGACCGGACCAGGAAGAAGAGAGGCGAGAAAGATGAGACGGAGTACGAGTACAGTGGCAGTGAGGAGGAGGAGGA

GGAAGTGCCTGAGCAGGAGGGAGAGCCAAGTTCCATCGTCAATGTGCCTGGAGAGTCGACTCTGCGACGTGATTT
GGAAGTGCCTGAGCAGGAGGGAGAGCCAAGTTCCATCGTCAATGTGCCTGGAGAGTCGACTCTGCGACGTGATTT

CCTGAGGCTGCAGCAAGAGAACAAGGAACGGTCTGAGGCTCTGCGGAGACAGCAGCTTCTGCAGGAGCAGCAGCT
CCTGAGGCTGCAGCAAGAGAACAAGGAACGGTCTGAGGCTCTGCGGAGACAGCAGCTTCTGCAGGAGCAGCAGCT

CCGGGAGCAGGAGGAGTATAAGAGGCAGCTGCTGGCTGAGAGGCAGAAGCGGATTGAACAGCAGAAAGAACAGAG
CCGGGAGCAGGAGGAGTATAAGAGGCAGCTGCTGGCTGAGAGGCAGAAGCGGATTGAACAGCAGAAAGAACAGAG

GAGGCGGCTGGAAGAGCAACAAAGGAGAGAGCGTGAAGCCAGGAGGCAGCAGGAACGTGAGCAGCGGCGGCGAGA
GAGGCGGCTGGAAGAGCAACAAAGGAGAGAGCGTGAAGCCAGGAGGCAGCAGGAACGTGAGCAGCGGCGGCGAGA

ACAGGAGGAGAAGAGGCGTCTGGAGGAACTGGAAAGGCGGCGTAAGGAAGAGGAAGAGAGGAGACGGGCAGAAGA
ACAGGAGGAGAAGAGGCGTCTGGAGGAACTGGAAAGGCGGCGTAAGGAAGAGGAAGAGAGGAGACGGGCAGAAGA

CGAGAAGAGGCGAGTGGAGCGGGAGCAGGAGTACATCAGGCGGCAGCTAGAGGAAGAGCAGCGGCACCTGGAGAT
CGAGAAGAGGCGAGTGGAGCGGGAGCAGGAGTACATCAGGCGGCAGCTAGAGGAAGAGCAGCGGCACCTGGAGAT
CCTGCAGCAGCAGCTGCTCCAGGAGCAGGCTATGTTACTGCACGACCACAGGAGGCCACACGCACAACAGCCGCC
CCTGCAGCAGCAGCTGCTCCAGGAGCAGGCTATGTTACTGCACGACCACAGGAGGCCACACGCACAACAGCCGCC

ACCCCCGCAGCAGCAGGACAGGAGCAAACCCAGCTATCACGCTCCAGAGCCCAAGCCTCACTACGACCCTGCTGA
ACCCCCGCAGCAGCAGGACAGGAGCAAACCCAGCTATCACGCTCCAGAGCCCAAGCCTCACTACGACCCTGCTGA

CAGAGCTCGGGAGGTACAGTGGTCCCACCTGGCATCTCTCAAGAACAATGTCTCCCCTGTCTCGAGATCCCATTC
CAGAGCTCGGGAGGTACAGTGGTCCCACCTGGCATCTCTCAAGAACAATGTCTCCCCTGTCTCGAGATCCCATTC

CTTCAGTGACCCTTCTCCCAAATTCGCACACCACCATCTCCGCTCTCAGGACCCATGTCCACCTTCCCGCAGTGA
CTTCAGTGACCCTTCTCCCAAATTCGCACACCACCATCTCCGCTCTCAGGACCCATGTCCACCTTCCCGCAGTGA

GGGGCTCAGTCAGAGCTCTGACTCTAAGTCGGAGGTACCTGAGCCCACCCAAAAGGCTTGGTCTAGATCAGACAG
GGGGCTCAGTCAGAGCTCTGACTCTAAGTCGGAGGTACCTGAGCCCACCCAAAAGGCTTGGTCTAGATCAGACAG

TGACGAGGTGCCTCCAAGGGTTCCTGTGAGAACGACATCTCGTTCCCCTGTCCTGTCCCGTCGAGATTCTCCACT
TGACGAGGTGCCTCCAAGGGTTCCTGTGAGAACGACATCTCGTTCCCCTGTCCTGTCCCGTCGAGATTCTCCACT

GCAGGGCAGTGGGCAGCAAAATAGCCAAGCAGGACAGAGAAATTCCACCAGCAGTATTGAGCCCCGGCTTCTATG
GCAGGGCAGTGGGCAGCAAAATAGCCAAGCAGGACAGAGAAATTCCACCAGCAGTATTGAGCCCCGGCTTCTATG

GGAGAGAGTGGAAAAGCTGGTCCCCAGGCCAGGCAGTGGCAGCTCTTCAGGGTCCAGCAACTCAGGATCCCAGCC
GGAGAGAGTGGAAAAGCTGGTCCCCAGGCCAGGCAGTGGCAGCTCTTCAGGGTCCAGCAACTCAGGATCCCAGCC

TGGCTCCCATCCTGGGTCTCAGAGTGGCTCTGGTGAACGCTTCAGAGTGAGATCATCATCCAAGTCTGAAGGTTC
TGGCTCCCATCCTGGGTCTCAGAGTGGCTCTGGTGAACGCTTCAGAGTGAGATCATCATCCAAGTCTGAAGGTTC

TCCATCTCAGCGCCTTGAAAATGCAGCCAAAAAACCTGAAGATAAGAAAGAAGTATTTCGACCTCTCAAACCTGC
TCCATCTCAGCGCCTTGAAAATGCAGCCAAAAAACCTGAAGATAAGAAAGAAGTATTTCGACCTCTCAAACCTGC

TGGAGAAGTGGACTTGACTGCGTTGGCCAAAGAGCTTCGAGCAGTGGAAGATGTGCGGCCACCTCACAAAGTGAC
TGGAGAAGTGGACTTGACTGCGTTGGCCAAAGAGCTTCGAGCAGTGGAAGATGTGCGGCCACCTCACAAAGTGAC

AGACTATTCCTCCTCCAGTGAGGAGTCTGGGACCACGGATGAGGAGGAGGAGGATGTGGAACAGGAGGGGGCTGA
AGACTATTCCTCCTCCAGTGAGGAGTCTGGGACCACGGATGAGGAGGAGGAGGATGTGGAACAGGAGGGGGCTGA

TGATTCCACCTCGGGGCCAGAGGACACCAGAGCAGCGTCGTCTCTGAATCTGAGCAACGGTGAGACAGAATCTGT
TGATTCCACCTCGGGGCCAGAGGACACCAGAGCAGCGTCGTCTCTGAATCTGAGCAACGGTGAGACAGAATCTGT

GAAAACAATGATTGTTCATGATGACGTAGAAAGTGAACCAGCCATGACCCCATCCAAGGAGGGCACCCTAATCGT
GAAAACAATGATTGTTCATGATGACGTAGAAAGTGAACCAGCCATGACCCCATCCAAGGAGGGCACCCTAATCGT

CCGCCAGACTCAGTCCGCTAGTAGCACACTCCAGAAACACAAATCTTCCTCCTCCTTTACACCTTTTATAGACCC
CCGCCAGACTCAGTCCGCTAGTAGCACACTCCAGAAACACAAATCTTCCTCCTCCTTTACACCTTTTATAGACCC

CAGGTTACTACAGATCTCTCCATCTAGTGGAACAACAGTTACTTCCGTTGTGGGATTTTCCTGTGATGGGCTGAG
CAGGTTACTACAGATCTCTCCATCTAGTGGAACAACAGTTACTTCCGTTGTGGGATTTTCCTGTGATGGGCTGAG

ACCAGAAGCCATAAGGCAAGATCCTACTCGGAAAGGCTCAGTGGTCAATGTGAACCCCACGAACACTAGGCCACA
ACCAGAAGCCATAAGGCAAGATCCTACTCGGAAAGGCTCAGTGGTCAATGTGAACCCCACGAACACTAGGCCACA

GAGTGATACCCCCGAGATTCGTAAATACAAGAAGAGATTTAACTCAGAGATCCTGTGTGCTGCCTTATGGGGAGT
GAGTGATACCCCCGAGATTCGTAAATACAAGAAGAGATTTAACTCAGAGATCCTGTGTGCTGCCTTATGGGGAGT

GAATTTGCTAGTTGGCACAGAGAGTGGCCTGATGCTCTTGGACAGAAGTGGCCAAGGGAAAGTGTACCCTCTGAT
GAATTTGCTAGTTGGCACAGAGAGTGGCCTGATGCTCTTGGACAGAAGTGGCCAAGGGAAAGTGTACCCTCTGAT

CAGCCGGAGACGGTTCCAGCAAATGGATGTACTTGAGGGCCTGAATGTCCTGGTGACAATATCTGGCAAAAAGGA
CAGCCGGAGACGGTTCCAGCAAATGGATGTACTTGAGGGCCTGAATGTCCTGGTGACAATATCTGGCAAAAAGGA

TAAGTTACGCGTCTACTATTTATCCTGGTTAAGAAATAAAATACTTCATAATGATCCGGAAGTTGAGAAGAAGCA
TAAGTTACGCGTCTACTATTTATCCTGGTTAAGAAATAAAATACTTCATAATGATCCGGAAGTTGAGAAGAAGCA

GGGGTGGACCACCGTGGGCGACCTGGAAGGGTGCGTGCACTATAAAGTCGTAAAATATGAAAGAATCAAGTTTCT
GGGGTGGACCACCGTGGGCGACCTGGAAGGGTGCGTGCACTATAAAGTCGTAAAATATGAAAGAATCAAGTTTCT

GGTAATCGCTTTGAAGAGTTCTGTGGAAGTCTATGCATGGGCACCGAAGCCATATCACAAATTTATGGCCTTTAA
GGTAATCGCTTTGAAGAGTTCTGTGGAAGTCTATGCATGGGCACCGAAGCCATATCACAAATTTATGGCCTTTAA

GTCATTTGGAGAACTGGTACATAAGCCATTACTGGTGGATCTCACTGTCGAGGAAGGCCAGAGGTTGAAAGTGAT
GTCATTTGGAGAACTGGTACATAAGCCATTACTGGTGGATCTCACTGTCGAGGAAGGCCAGAGGTTGAAAGTGAT

CTATGGATCCTGTGCTGGATTCCATGCTGTTGATGTGGATTCCGGCTCAGTCTATGACATTTATCTACCAACACA
CTATGGATCCTGTGCTGGATTCCATGCTGTTGATGTGGATTCCGGCTCAGTCTATGACATTTATCTACCAACACA

CATTCAGTGTACCATCAAACCCCATGCAATCATTATCCTCCCCAACACGGATGGAATGGAGCTTCTGGTGTGCTA
CATTCAGTGTACCATCAAACCCCATGCAATCATTATCCTCCCCAACACGGATGGAATGGAGCTTCTGGTGTGCTA

CGAAGATGAAGGGGTTTATGTGAACACGTACGGAAGAATCACCAAGGACGTGGTTCTGCAGTGGGGAGAGATGCC
CGAAGATGAAGGGGTTTATGTGAACACGTACGGAAGAATCACCAAGGACGTGGTTCTGCAGTGGGGAGAGATGCC

AACATCTGTAGCATACATCCGGTCGAATCAGACAATGGGCTGGGGAGAGAAGGCAATAGAGATACGCTCTGTGGA
AACATCTGTAGCATACATCCGGTCGAATCAGACAATGGGCTGGGGAGAGAAGGCAATAGAGATACGCTCTGTGGA

AACTGGTCATTTGGACGGTGTGTTTATGCACAAAAGGGCTCAGAGACTAAAGTTCCTGTGTGAACGTAATGACAA
AACTGGTCATTTGGACGGTGTGTTTATGCACAAAAGGGCTCAGAGACTAAAGTTCCTGTGTGAACGTAATGACAA

GGTGTTCTTTGCTTCCGTCCGCTCTGGCGGCAGCAGCCAGGTCTACTTCATGACCTTAGGCAGGACTTCCCTTCT
GGTGTTCTTTGCTTCCGTCCGCTCTGGCGGCAGCAGCCAGGTCTACTTCATGACCTTAGGCAGGACTTCCCTTCT

GAGCTGGTAGAGGCAGTGTGAGCCGGGATCACTGACCTCTGGAGTCGGCAAGACCCCAAGAACTTGGAATTCCTT
GAGCTGGTAG---

GCAACTGGAGCTCAGAGCTGTGCCGGTGCAGCCATGACAGCTGTCTATGCAGGCCTCTTGTGGCGTGGCGTGTCG
GGTCGGGTCCTCTCCCCTCCCTCCTACACTTTCTGCCAGTTTATTCCCATTCTCTCCTCCTCCTTTCTCAAAAAA
ATAAACCAAGGCTGCATTGCAGCTGGTGCTGTTCAGACTCTACCATCGGTGCTAGGAATGCTTGGGGATGAACAC
ATCAGACCGGGAAGAGCGCCTTTCCTACAGCTCCAGGATTCCTCGTCTCCGAGCCACTGTCCCGTGGGCGTCTGC
TGGTTGTGACGATTGCGCCACCCGACGTTGGCGGCAGGCACAGAGAGGTGAAAAGTTGGTGTAAGAAGCCGCTTG
CTGAAGCAGAGAACAGAGTTAATATAGTAACATTAACAATGTATTTAATTGACATTTCTCTCTTTTTGTAATGTG
ACAGTATGTGGACAAAGAAGAAGGTGCAGGTCTAAGAGGTTAATATTTATAAAATGTGAAAGACACAGTTACTAG
GATAACTTTTTTTGTGGGTGGGGCTTGGGGTGGGGCAGGGCGGGTTAAAGGGTCCCATTTTGTTTCTTTGAAAGC
TTTTTGGAGAGGGTTGTCCTGGCCAAGAACTCAGTCATTTTTCTGTGTACCAAGTTTTGCCTAAATTGTGCAGAT
GGTTCTTAAAAAAGAAAGGAAAGGAAAAGGAAATGTGTGCGTCTTGTTGGAGGGCCAGAAGTTTTGTTGTGTAAC
AGTATTAGCAAGGCCTGGGCTAAGGGTTTAATGTCCGTTAAGCCTAAAGTGCGGCAGCAGCTCCACCACGGTCTG
CATGCACCATAGGAAAGGCCAGCTTTCCTGTGGGTTCTCATTTCGTTTCGTTTTGTTTTTTGCCTACCTCATTGT
TCTTAATGCATTGAGAGGTGACTTAGCTTAATGGTTTTGGCAGAAAACATTTAATGTTTAATTTAACCTTAATAC
CAAAACTATCTGATTAAAGTTTGACTGACTTTGTCACAAGTCTAATCAGGTACAAAAAAAAAAGGAAAAGAAAAG
AGAAAGAAAGAAAGAAAGCTGTCTATGATGGTGGGAGCAGCCTCAGCACTCAGACGCTCTGCTCTCACCAAGTTC
ACCATTGGAGGGTGACAGGCAGCGGAGTCACCTGTCCTGACAAGAGATCTCACTGTTCGCAGTTACAGGCTTTGG
AAGCAGGCAGGGGTCACACTAGGAGTCACACAGACTATTCATTCATGTTGCAGGAACTTTTTCTTGGGAGCGAGG
GTGTGTCCCTTTTCTTAAAAAAAAACAAAAAGCAAAAAAAACAAAAAAAACCCCGCAATGCACTAAAACTATTTT
AAGAATGTAGTTACTCCTACTTCTTCATAGACGGCACCTCCCTGCCTGTGTTTCAGGTGTAAGACTGACGCTCTG
GCTTTTCCTGTTTCTCACGTGCTCTCTTGTTTGTTCTCTCTCTTTTTTTTAAAACTAATTTTTCTTTATGAA
TATGTCTGTGACATTTGTAATAAATGTCTTGTAATAATTTGC

Supplemental Figure S4. Protein sequence of potential full-length rat Map4k4 containing all possible exons (upper rows with individual exons in yellow and green alternating) compared with the 1233 residue Map4k4 cloned from neonatal rat cardiomyocytes (lower rows, bold type). Map4k4 1233 is missing exons 16 and 24, plus 5' sequence from exon 15 and 3'sequence from exons 20 and 30 (missing extensions in italics). Residues highlighted in red are coded across an exon boundary. Exon numbers are provided above the potential full-length sequence.

1 2 3 4
MANDSPAKSLVDIDLSSLRDPAGIFELVEVVGNGTYGQVYKGRHVKTGQLAAIKVMDVTEDEEEEIKLEINMLKKYSHHR
MANDSPAKSLVDIDLSSLRDPAGIFELVEVVGNGTYGQVYKGRHVKTGQLAAIKVMDVTEDEEEEIKLEINMLKKYSHHR

5 6
NIATYYGAFIKKSPPGHDDQLWLVMEFCGAGSITDLVKNTKGNTLKEDWIAYISREILRGLAHLHIHHVIHRDIKGQNVL
NIATYYGAFIKKSPPGHDDQLWLVMEFCGAGSITDLVKNTKGNTLKEDWIAYISREILRGLAHLHIHHVIHRDIKGQNVL

7 8 9
LTENAEVKLVDFGVSAQLDRTVGRRNTFIGTPYWMAPEVIACDENPDATYDYRSDLWSCGITAIEMAEGAPPLCDMHPMR
LTENAEVKLVDFGVSAQLDRTVGRRNTFIGTPYWMAPEVIACDENPDATYDYRSDLWSCGITAIEMAEGAPPLCDMHPMR

10 11
ALFLIPRNPPPRLKSKKWSKKFFSFIEGCLVKNYMQRPSTEQLLKHPFIRDQPNERQVRIQLKDHIDRTRKKRGEKDETE
ALFLIPRNPPPRLKSKKWSKKFFSFIEGCLVKNYMQRPSTEQLLKHPFIRDQPNERQVRIQLKDHIDRTRKKRGEKDETE

12
YEYSGSEEEEEEVPEQEGEPSSIVNVPGESTLRRDFLRLQQENKERSEALRRQQLLQEQQLREQEEYKRQLLAERQKRIE
YEYSGSEEEEEEVPEQEGEPSSIVNVPGESTLRRDFLRLQQENKERSEALRRQQLLQEQQLREQEEYKRQLLAERQKRIE

13 14
QQKEQRRRLEEQQRREREARRQQEREQRRREQEEKRRLEELERRRKEEEERRRAEDEKRRVEREQEYIRRQLEEEQRHLE
QQKEQRRRLEEQQRREREARRQQEREQRRREQEEKRRLEELERRRKEEEERRRAEDEKRRVEREQEYIRRQLEEEQRHLE

15
ILQQQLLQEQAMLL*ECRWREMEEHRQAERLQRQLQQEQAYLLSLQ*HDHRRPHAQQPPPPQQQDRSKPSYHAPEPKPHYDP
ILQQQLLQEQAMLL-------------------------------HDHRRPHAQQPPPPQQQDRSKPSYHAPEPKPHYDP

16 17
ADRAREVEDRFRKTNHSSPEAQAKQTGRGLEPPVPSRSESFSNGNSESVHPALQRPAEPQVQWSHLASLKNNVSPVSRSH
ADRARE--VQWSHLASLKNNVSPVSRSH

18
SFSDPSPKFAHHHLRSQDPCPPSRSEGLSQSSDSKSEVPEPTQKAWSRSDSDEVPPRVPVRTTSRSPVLSRRDSPLQGSG
SFSDPSPKFAHHHLRSQDPCPPSRSEGLSQSSDSKSEVPEPTQKAWSRSDSDEVPPRVPVRTTSRSPVLSRRDSPLQGSG

19 20
QQNSQAGQRNSTSSIEPRLLWERVEKLVPRPGSGSSSGSSNSGSQPGSHPGSQSGSGERFRVRSSSKSEGSPSQRLENAA
QQNSQAGQRNSTSSIEPRLLWERVEKLVPRPGSGSSSGSSNSGSQPGSHPGSQSGSGERFRVRSSSKSEGSPSQRLENAA

21 22
KKPEDKKEVFRPLKPA*VRI*GEVDLTALAKELRAVEDVRPPHKVTDYSSSSEESGTTDEEEEDVEQEGADDSTSGPEDTRA
KKPEDKKEVFRPLKPA---GEVDLTALAKELRAVEDVRPPHKVTDYSSSSEESGTTDEEEEDVEQEGADDSTSGPEDTRA

23 24
ASSLNLSNGETESVKTMIVHDDVESEPAMTPSKEGTLIVRQSTVDQKRASHHESNGFAGRIHLLPDLLQQSHSSSTSSTS
ASSLNLSNGETESVKTMIVHDDVESEPAMTPSKEGTLIVRQ-----------------------------------

25 26
SSPSSSQPTPTMSPQTPQDKLTANETQSASSTLQKHKSSSSFTPFIDPRLLQISPSSGTTVTSVVGFSCDGLRPEAIRQD
-------------------------TQSASSTLQKHKSSSSFTPFIDPRLLQISPSSGTTVTSVVGFSCDGLRPEAIRQD

27
PTRKGSVVNVNPTNTRPQSDTPEIRKYKKRFNSEILCAALWGVNLLVGTESGLMLLDRSGQGKVYPLISRRRFQQMDVLE
PTRKGSVVNVNPTNTRPQSDTPEIRKYKKRFNSEILCAALWGVNLLVGTESGLMLLDRSGQGKVYPLISRRRFQQMDVLE

28 29
GLNVLVTISGKKDKLRVYYLSWLRNKILHNDPEVEKKQGWTTVGDLEGCVHYKVVKYERIKFLVIALKSSVEVYAWAPKP
GLNVLVTISGKKDKLRVYYLSWLRNKILHNDPEVEKKQGWTTVGDLEGCVHYKVVKYERIKFLVIALKSSVEVYAWAPKP

30 31
YHKFMAFKSFGELVHKPLLVDLTVEEGQRLKVIYGSCAGFHAVDVDSGSVYDIYLPTH*VRKNPHSM*IQCTIKPHAIIILP
YHKFMAFKSFGELVHKPLLVDLTVEEGQRLKVIYGSCAGFHAVDVDSGSVYDIYLPTH--------IQCTIKPHAIIILP

32
NTDGMELLVCYEDEGVYYVNTYGRITKDVVLQWGEMPTSVAYIRSNQTMGWGEKAIEIRSVETGHLDGVFMHKRAQRLKFL
NTDGMELLVCYEDEGVYYVNTYGRITKDVVLQWGEMPTSVAYIRSNQTMGWGEKAIEIRSVETGHLDGVFMKRAQRLKFLH

33
CERNDKVFFASVRSGGSSQVYFMTLGRTSLLSW
CERNDKVFFASVRSGGSSQVYFMTLGRTSLLSW

Supplemental Table S1. Primers used for MAP4K4 cloning and mutation.

Primer ID	Sequence (5' to 3')	Notes
F1	AACTCAGAGATCCTGTGTGC	
F2	CTGTCTCGAGATCCCATTCC	Includes an endogenous Xho1 site (underlined
F3	AGAGAGGCGAGAAAGATGAG	
F4	GACGACAAGGGTACCATGGCGAACGACTCTCCGGC	Includes a KpnI site adjacent to the ATG start codon
F5	GGTCTATATAAGCAGAGCTG	
F6	AGCTGGCGGCCATCA**G**GGTTATGGATGTCAC	mutant base to generate K54R underlined
F7	GTTGGACGGAGAAAT**G**CATTCATAGGCACAC	mutant base to generate T187A underlined
F8	ATCCAGCTTAAGGAGGAGGAGGAGGAGGAAGTGC	AflII site underlined
F9	TCTGGAGGAACTGGAAAGGC	
F10	CGCGGAGGTGAAACTTGTTG	
F11	CCATGCAATCATTATCCTC	
F12	GAGCTC**TCGA**GGGAGGTTCCTGTGAGAACGACATCTCG	Includes an additional four bases (TCGA, emboldened) to generate the Xho1 site (underlined)
F13	GTTGGACGGAGAAAT**GAC**TTCATAGGCACAC	mutant bases to generate T187D underlined
F14	CCCTGTCTCGAG**A**TGTGCTGCCTTATGGGGAGTGA	The additional A is needed to keep the correct reading frame.
F15	CGAGAAAGATGAGGCGGAGTACGAGTACGCTGGCGCT GAGGAGGAGGAGG	
F16	GATCACATAGACCGGGCCAGGAAGAAGAGAGGC	
R1	CTTATCTAGAAGCTTCTACCAGCTCAGAAGGGAAG	Includes a HindIII site adjacent to the stop codon TAG (CTA)
R2	TCACTCCCCATAAGGCAGC	
R3	TCACAGGAACCCTTGGAG	
R4	CTCGTACTCCGTCTCATCTTTC	
R5	GTGACATCCATAACC**C**TGATGGCCGCCAGCT	mutant base to generate K54R underlined
R6	CTCAGGAAATCACGTCGCAG	
R7	GTGTGCCTATGAATG**C**ATTTCTCCGTCCAAC	mutant base to generate T187A underlined
R8	CACACCAGAAGCTCCATTCC	
R9	CAGCTGCTGCTGCAGCATCT	
R10	CTCGAC**TCGA**GAGCTCTGTCAGCAGGGTCGTAG	Includes an additional four bases (TCGA, emboldened) to generate the Xho1 site (underlined)
R11	GTGGTATGGCTGATTATGATCAG	
R12	GTGTGCCTATGAA**GTC**ATTTCTCCGTCCAAC	mutant bases to generate T187D underlined
R13	GGATCGGATATCCTACAGGATCTCTGAGTTAAATCTC	
R14	CCTCCTCCTCCTCAGCGCCAGCGTACTCGTACTCCGCC TCATCTTTCTCG	
R15	GCCTCTCTTCTTCCTGGCCCGGTCTATGTGATC	

Supplemental Table S2. Mass-spectrometry of MAP4K4 expressed in cardiomyocytes. Rat neonatal cardiomyocytes were infected with adenoviruses for expression of FLAG-tagged MAP4K4 and treated without or with calyculin A (CalA; 100 nM, 10 min). MAP4K4 was immunoprecipitated using EZ-FLAG and proteins separated by SDS-PAGE. FLAG-MAP4K4 was extracted from gels stained with Coomassie blue (see **Figure 2A**) and digested with trypsin. Peptide fragments and post-translational modifications (PTMs) were identified by mass spectrometry. Full details of peptides and PTMs are shown in Supplemental Spreadsheet S1.

**Treatment**	**No. of peptides**	**% coverage**	**No. of PTMs**
Minus CalA	124	78.4	820
Plus CalA	121	82.4	227

Supplemental Table S3. Identification of proteins in Band 3. Rat neonatal cardiomyocytes were infected with adenoviruses for expression of FLAG-tagged MAP4K4 and treated without or with calyculin A (CalA; 100 nM, 10 min). The expressed proteins were immunoprecipitated using EZ-FLAG and separated by SDS-PAGE. Band 3 was extracted from gels stained with Coomassie blue (see **Figure 2A**) and analysed by mass spectrometry following digestion with trypsin for protein identification. Proteins identified with >20 peptides and >20% coverage are shown.

**Accession**	**Gene symbol**	**Gene name**	**% Coverage**	**No. of peptides**	**Score**
IPI00189809.2	Myh6	Myosin heavy chain 6	72.00	218	38996.92
IPI00189811.1	Myh7	Myosin heavy chain 7	69.87	214	37413.99
IPI00209113.3	Myh9	Myosin heavy chain 9	69.40	193	22081.04
IPI00391300.3	Myo10	Myosin X	60.31	140	11747.71
IPI00764167.1	Myh11	Myosin heavy chain 11	38.61	71	6025.76
IPI00957592.1	Myh11	Myosin heavy chain 11	38.39	71	5964.10
IPI01007580.1	Myh11	Myosin heavy chain 11	37.44	68	5724.38
IPI00366370.3	Eif5b	Eukaryotic translation initiation factor 5B	30.10	31	1264.12
IPI00367479.5	Myh14	Myosin 14	29.70	47	3273.00
IPI00764433.3	MAP4K4	Mitogen-activated protein kinase kinase kinase kinase 4	24.70	24	982.55
IPI00568786.4	MAP4K4	Mitogen-activated protein kinase kinase kinase kinase 4	23.53	24	983.65

Supplemental Table S4. MAP4K4 peptide fragments associated with Band 3.

Residues	Region	Peptides	% coverage
1-289	Kinase domain	SPPGHDDQLWLVMEFcGAGSITDLVK<br>GQNVLLTENAEVK<br>LVDFGVSAQLDR<br>ALFLIPR<br>FFSFIEGCLVK	14.1
290-492	Coiled-coil	RQQLLQEQQLR<br>RLEEQQR	8.9
493-919	Unstructured linker	SKPSYHAPEPK<br>EVQWSHLASLK<br>SEGLSQSSDSKSEVPEPTQK<br>RDSPLQGSGQQNSQAGQR<br>NSTSSIEPR<br>LVPRPGSGSSSGSSNSGSQPGSHPGsQSGSGERFR<br>AASSLNLSNGETESVK<br>QTQSASSTLQK	30.2
920-1233	Citron homology domain	FQQMDVLEGLNVLVTISGK<br>VYYLSWLR<br>ILHNDPEVEKK<br>FLVIALK<br>SSVEVYAWAPK<br>DVVLQWGEMPTSVAYIR	23.2
